# Water supply and runoff capture reliability curves for hypothetical rainwater harvesting systems for locations across the U.S. for historical and projected climate conditions

**DOI:** 10.1016/j.dib.2018.03.024

**Published:** 2018-03-11

**Authors:** Nasrin Alamdari, David J. Sample, Jia Liu, Andrew Ross

**Affiliations:** a Department of Biological System Engineering, Virginia Polytechnic Institute and State University, Blacksburg, VA 24061, United States; b Hampton Roads Agricultural Research and Extension Center, Virginia Polytechnic and State University, Virginia Beach, VA 23455, United States; c Water Resources Engineer, Charles P. Johnson & Associates, Inc., Washington D.C, United States; d Department of Meteorology, Pennsylvania State University, University Park, PA 16802, United States

## Abstract

The data presented in this article are related to the research article entitled “Assessing climate change impacts on the reliability of rainwater harvesting systems” (Alamdari et al., 2018) [1]. This article evaluated the water supply and runoff capture reliability of rainwater harvesting (RWH) systems for locations across the U.S. for historical and projected climate conditions. Hypothetical RWH systems with varying storage volumes, rooftop catchment areas, irrigated areas, and indoor wSater demand based upon population from selected locations were simulated for historical (1971–1998) and projected (2041–2068) periods, the latter dataset was developed using dynamic downscaling of North American Regional Climate Change (CC) Assessment Program (NARCCAP). A computational model, the Rainwater Analysis and Simulation Program (RASP), was used to compute RWH performance with respect to the reliability of water supply and runoff capture. The reliability of water supply was defined as the proportion of demands that are met; and the reliability of runoff capture was defined as the amount stored and reused, but not spilled. A series of contour plots using the four design variables and the reliability metrics were developed for historical and projected conditions. Frequency analysis was also used to characterize the long-term behavior of rainfall and dry duration at each location. The full data set is made publicly available to enable critical or extended analysis of this work.

**Specifications Table**

**Table:** 

Subject area	*Hydrology and Water Resources*
More specific subject area	*Climate Change and Rainwater Harvesting Systems*
Type of data	*Figures*
How data was acquired	*Modeling*
Data format	*Analyzed*
Experimental factors	*Historical and projected data from 17 stations listed in*Table 1*were downloaded from North American Regional CC assessment program (NARCCAP). The historical hourly observation rainfall data from 1971–1998 were obtained from National Climate Data Center (NCDC) (*https://www.ncdc.noaa.gov/*); this data was used for bias correction of NARCCAP historical and projected climate simulations for the same locations. NARCCAP provides model output at three-hourly intervals. A disaggregation method was then applied to convert precipitation data to hourly time step.*
Experimental features	*NA*
Data source location	*Country: United States of America*
Data accessibility	*The data are available with this article.*

**Value of the data**

• The data provide reliability contour plots for hypothetical RWH systems implemented across the U.S. for historical and projected climate conditions.
• The data are useful for identifying locations in which RWH systems that provide the greatest benefits, and for selecting climate resilient stormwater management strategies.
• The data allows other researchers to extend the analysis and make comparisons.

## 1 Data

Water supply reliability, *λ*_*WS*_, is a dimensionless number ranging from 0 to 1 that reflects the ability of RWH systems to meet nonpotable indoor uses and outdoor irrigation water demand. Similarly, runoff capture reliability, *λ*_*RC*_, is defined as the amount stored and reused, but not spilled. *λ*_*WS*_ and *λ*_*RC*_ were obtained from simulations of historical and projected climate conditions for the independent variables storage volume, or *TankV*, roof area, or *RoofA*, irrigated area, or *IrArea*, and indoor demand or *Pop* using an average per capita water demand of 22.7L/person/d. A series of simulations using the four design variables and the reliability metrics were developed for historical and projected conditions. Frequency analysis was also used to characterize the long-term behavior of rainfall and dry duration at each site.

## 2 Experimental design, materials, and methods

We selected 17 stations across the U.S. for evaluating hypothetical RWH systems. Projections for rainfall across the U.S. from the North American Regional CC assessment program (NARCCAP) [2] were used as input data for historical and projected conditions. The historical hourly observation rainfall data from 1971–1998 were obtained from National Climate Data Center (NCDC) (https://www.ncdc.noaa.gov/); this data was used for bias correction of NARCCAP historical and projected climate simulations for the same locations in a procedure described in [3]. NARCCAP provides model output at three-hourly intervals. A disaggregation method was then applied to convert precipitation data to hourly time step. The reader is referred to [1], a research paper associated with this data for disaggregation and bias correction methods. Table 1 presents the features of the 17 selected stations with their locations across the U.S.

**Table 1:** Selected weather stations across the U.S.

**City**	**Station**	**COOPID**	**Latitude/Longitude**
**Charleston**	Charleston International Airport, SC	381544	32.89 N/ 80.04W
**Chicago**	Chicago O’Hare International Airport, IL	111549	41.98 N/ 87.84 W
**Dallas**	Fort Worth WSFO, TX	413285	32.77 N**/** 97.31 W
**Denver**	Denver International Airport, CO	052211	39.85 N/ 104.67 W
**Houston**	Houston Intercontinental Airport, TX	414300	29.99 N/ 95.34 W
**Kansas City**	Kansas City International Airport, MO	234358	39.30 N**/** 94.71 W
**Los Angeles**	Los Angeles International Airport, CA	045114	33.94 N**/** 118.41 W
**Miami**	Miami International Airport, FL	085663	25.79 N**/** 80.28 W
**Memphis**	Memphis International Airport, TN	405954	35.04 N**/** 89.98 W
**New Orleans**	New Orleans International Airport, LA	166660	29.99 N**/** 90.26 W
**New York**	JFK International Airport, NY	305803	40.64 N**/** 73.78 W
**Norfolk**	Norfolk International Airport, VA	446139	36.90 N**/** 76.19 W
**Salt Lake City**	Salt Lake City International Airport, UT	427598	40.79 N**/** 111.98 W
**San Francisco**	San Francisco International Airport, CA	047769	37.62 N**/** 122.39 W
**Seattle**	Seattle Tacoma International Airport, WA	457473	47.44 N**/** 122.30 W
**Tampa**	Tampa International Airport, FL	088788	27.98 N**/** 82.54 W
**Washington**	Washington Reagan National Airport, VA	448906	38.85 N**/** 77.04 W

### 2.1 Frequency analysis

**Fig. 1:**
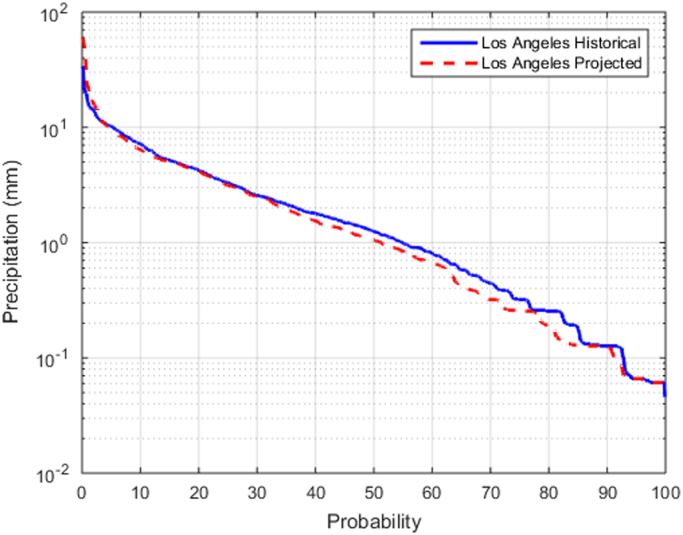
Frequency of rainfall events for Los Angeles for historical and projected conditions.

**Fig. 2:**
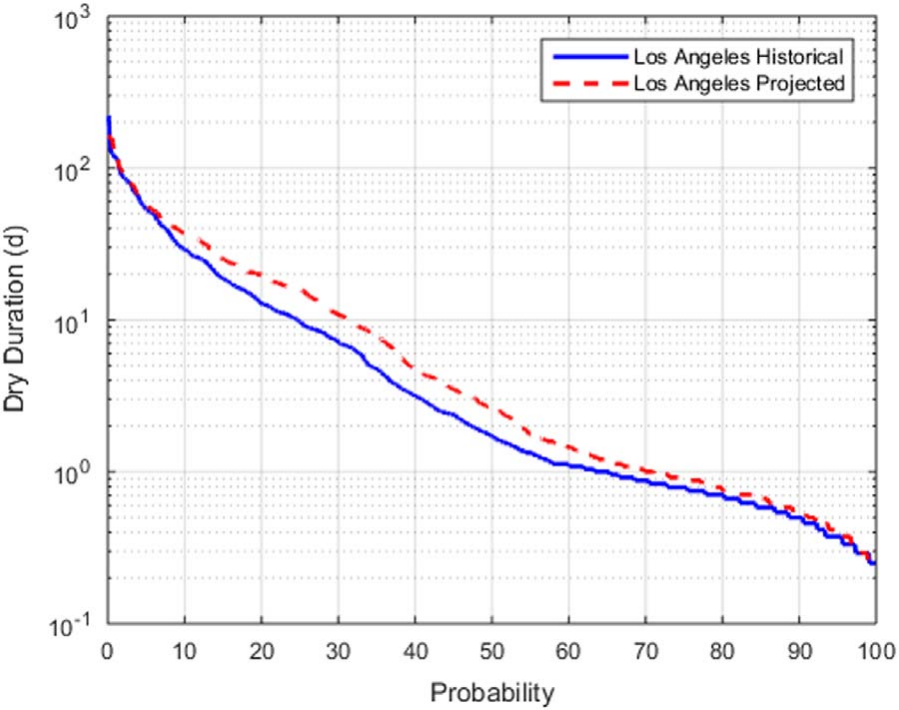
Frequency of dry duration for Los Angeles for historical and projected conditions.

Frequency analysis of rainfall events and dry durations based upon precipitation data for historical and projected periods across the U.S. was used to characterize long-term rainfall patterns. Historical and projected hourly data were processed into events using multiple inter-event times (dry period between events). Processed precipitation data was imported to the computational model for evaluating the frequency distribution of rainfall events [4]. A similar procedure using frequency analysis was applied to assess the exceedance probabilities of dry durations after filtering out smallest (≤2.54 mm) rainfall events. Dry duration between any two consecutive rainfall events was computed from the sorted ranks. The occurrence frequency for dry duration for historical and projected periods at each site were plotted and compared. The frequency of Rainfall and dry duration for Los Angeles with and without CC is provided in Fig. 1, Fig. 2 as an example. The remaining frequency plots have been placed in Appendix A and Appendix B for historical and projected CC conditions, respectively.

### 2.2 RWH model description

The Rainwater Analysis and Simulation Program (RASP), a program written in the MATLAB^®^ environment, [5], was used to simulate performance of various RWH s at the selected locations. The RASP code is available at no cost at https://github.com/RainwaterHarvesting/Rainwater-Analysis-and-Simulation-Program. The main components of an RWH are storage tank, a roof catchment area, a filtration device and pumping (if required). The storage tank is designed to reserve a minimum 10% of total storage to keep pumps primed. The program is able to run for multiple or single scenarios. The main design variables in this program are *TankV*, *RoofA*, *IrArea*, and *Pop*. The RASP model is based on the behavioral method which uses an algorithm to simulate the flow of water through the reservoir. The yield before storage (YBS) is calculated to achieve the storm water management goal. YBS algorithm is adapted from [6]. When the capacity of the storage is full, a spill occurs. Water from RWH typically is used either indoor nonpotable or outdoor irrigation. The reader is referred to [7] for details on the equations, variable definitions, and limitations of the RASP model. After *λ*_*WS*_ and *λ*_*RC*_ were calculated, a series of contour plots were then developed with the reliability metrics as dependent variables for historical and projected conditions. The reader is referred to [1], a research paper associated with this data for more details and analysis of results and conclusions.

### 2.3 RWH model results for historical and projected climate conditions

To predict future CC impacts on RWH functionality, projections for precipitation, across different sites are required. The period 1971–1998 was used as the historical baseline, and 2041–2068 for the future projection. We selected a medium-high greenhouse gas emissions scenario, A2 [8] for climate projections.

**Fig. 3:**
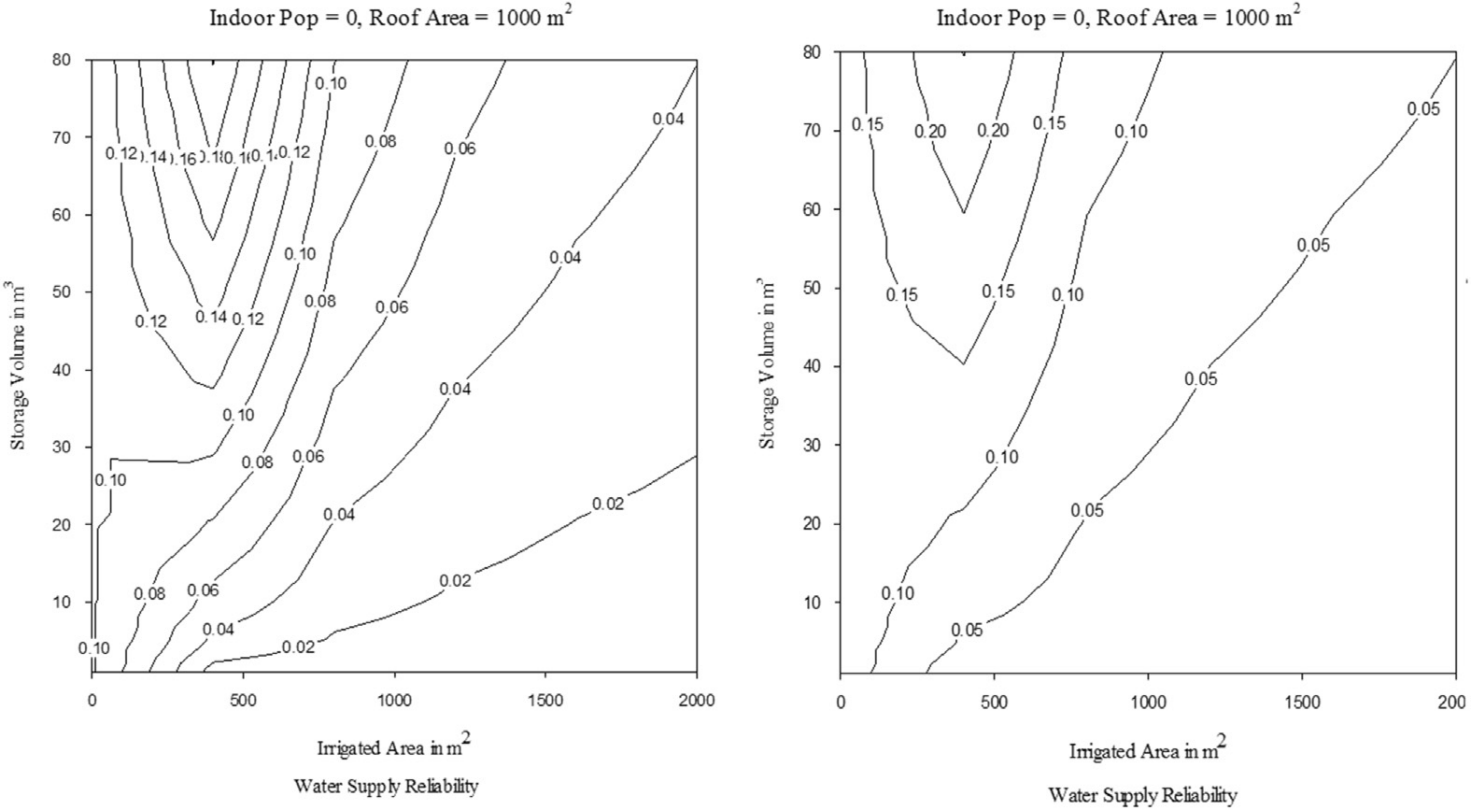
Water supply reliability for Los Angeles for historical and projected conditions, for *RoofA*=1000 m^2^ and *Pop*=0.

**Fig. 4:**
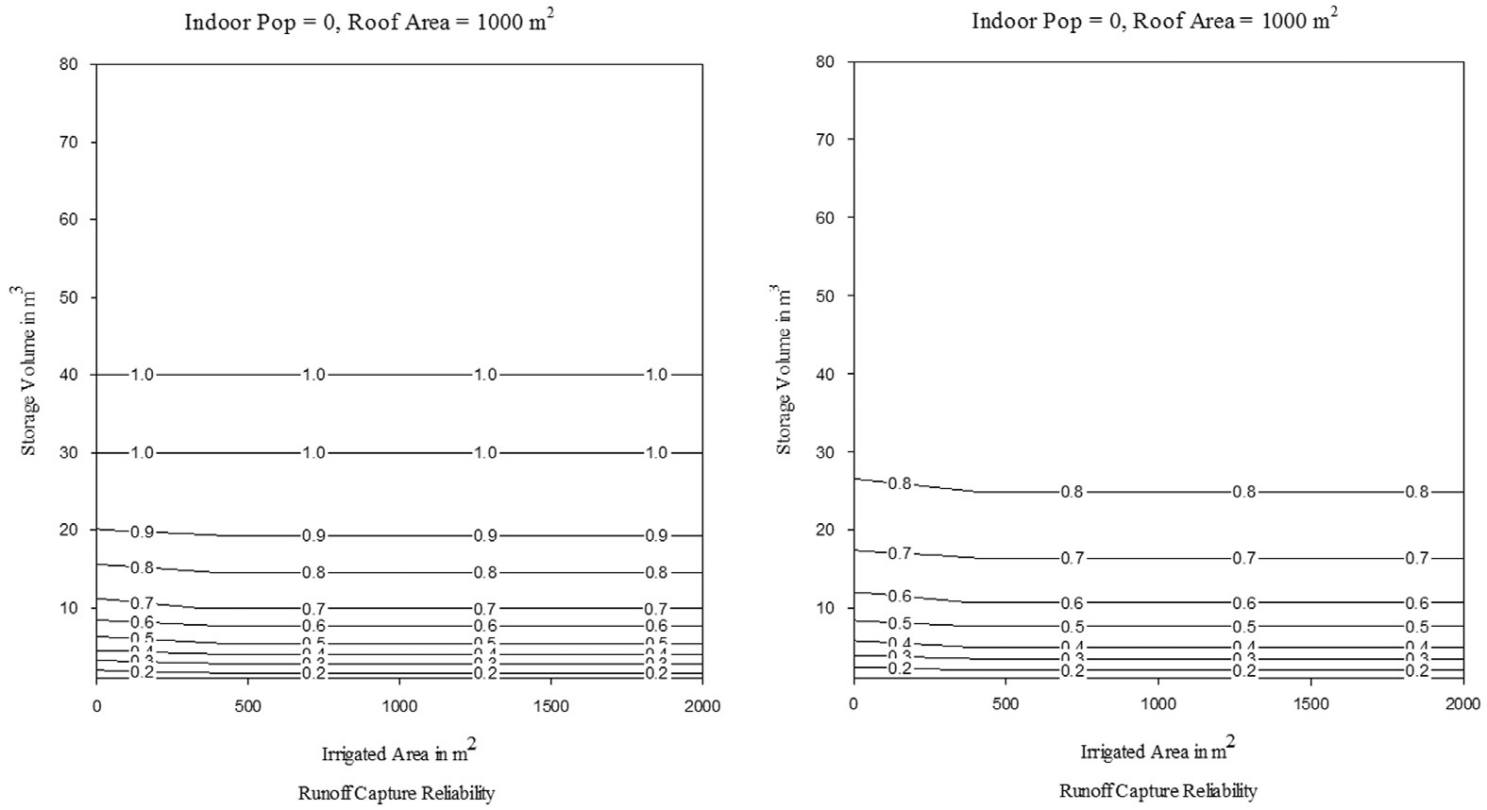
Runoff capture reliability for Los Angeles for historical and projected conditions, for *RoofA*=1000 m^2^ and *Pop*=0.

**Fig. 5:**
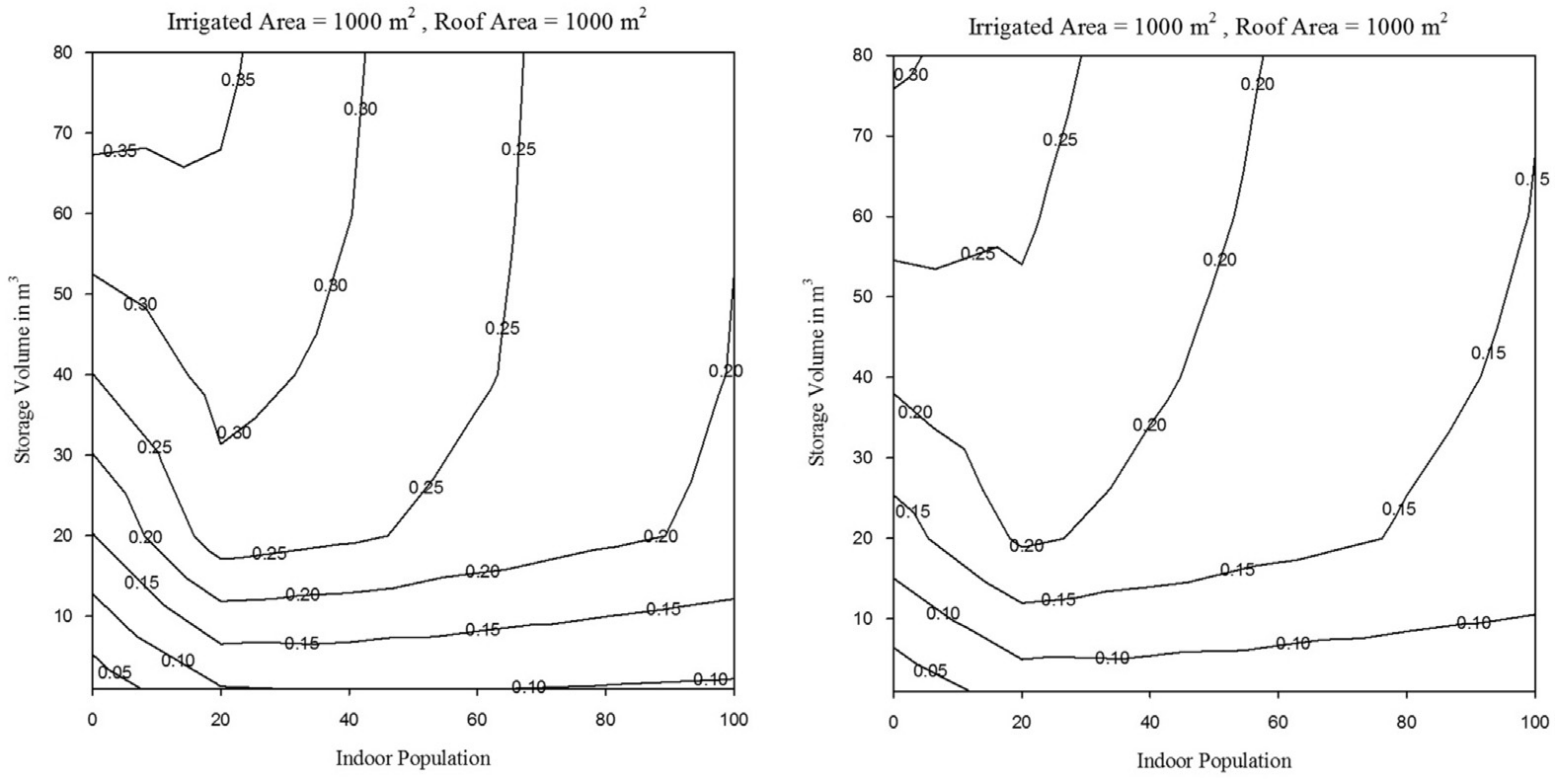
Water supply reliability for Los Angeles for historical and projected conditions, for *RoofA*=1000 m^2^ and *IrArea*=1000 m^2^.

**Fig. 6:**
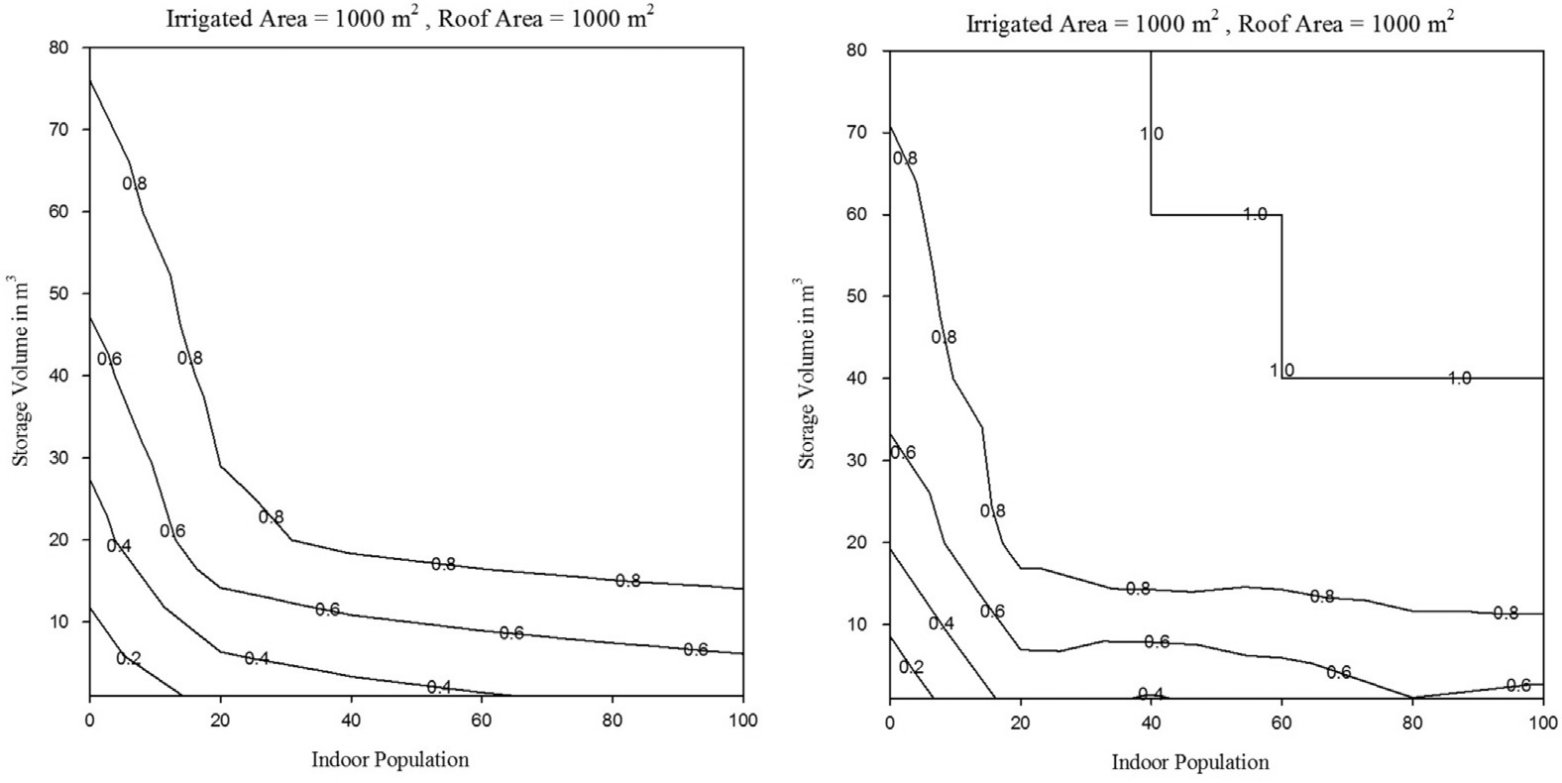
Runoff capture reliability for Los Angeles for historical and projected conditions, for *RoofA*=1000 m^2^ and *IrArea*=1000 m^2^.

The MM5I-CCSM was selected among nine NARCCAP models as the pilot scenario. Biases that could affect the hydrological model simulations were corrected using modified version of the equiratio cumulative distribution function matching method [9]. The reader is referred to [3] for more detail on the bias correction method. NARCCAP provides model output at three-hourly intervals, which is not sufficient temporal scale for precipitation. A disaggregation method was applied to convert precipitation data to hourly time step using a method developed by [10]. The A2 climate scenarios were input into the RASP model to facilitate comparison. In this study, the impacts of CC on *λ*_*WS*_ and *λ*_*RC*_ of RWH for historical and projected conditions was assessed. A series of contour plots were developed for historical and projected conditions to evaluate *λ*_*WS*_ and *λ*_*RC*_ for each of the selected locations; these are provided in Appendix C and Appendix D, respectively. As an example, water supply and runoff capture reliability for Los Angeles for a *RoofA*=1000 m^2^ and *Pop=0* are presented for *TankV* as a function of *IrArea* in Fig. 3, Fig. 4, respectively. Water supply and runoff capture reliability for Los Angeles for a *RoofA*=1000 m^2^ and *IrArea*=1000 m^2^ are presented for *TankV* as a function of *Pop* in Fig. 5, Fig. 6, respectively.
