# Supplementary material for: Water supply and runoff capture reliability curves for hypothetical rainwater harvesting systems for locations across the U.S. for historical and projected climate conditions
Source: Data Brief. 2018 Mar 11;18:441–7. doi: 10.1016/j.dib.2018.03.024 (PMC5996225; doi:10.1016/j.dib.2018.03.024)
Supplement: Supplementary file 1 — Supplementary material [file mmc1.pdf]

AUTHOR DECLARATION TEMPLATE

We wish to draw the attention of the Editor to the following facts which may be considered as potential conflicts of interest and to significant financial contributions to this work. The work was funded partially by the National Science Foundation project, Water Sustainability and Climate WSC-Category 1 Collaborative Project: Coupled Multi-Scale Economic, Hydrologic and Estuarine Modeling to Assess Impacts of Climate Change on Water Quality Management, Grant #23032, and the the Virginia Agricultural Experiment Station and the Hatch program of the National Institute of Food and Agriculture. To the best of our knowledge, this funding has not presented a conflict of interest.

We confirm that the manuscript has been read and approved by all named authors and that there are no other persons who satisfied the criteria for authorship but are not listed. We further confirm that the order of authors listed in the manuscript has been approved by all of us.

We confirm that we have given due consideration to the protection of intellectual property associated with this work and that there are no impediments to publication, including the timing of publication, with respect to intellectual property. In so doing we confirm that we have followed the regulations of our institutions concerning intellectual property.

We understand that the Corresponding Author is the sole contact for the Editorial process (including Editorial Manager and direct communications with the office). He/she is responsible for communicating with the other authors about progress, submissions of revisions and final approval of proofs. We confirm that we have provided a current, correct email address which is accessible by the Corresponding Author and which has been configured to accept email from dsample@vt.edu

Signed by all authors as follows:

Nasrin Alamdari
alamdari@vt.edu

David J. Sample
dsample@vt.edu

Jia Liu
liujia@vt.edu

Andrew Ross
acr5155@psu.edu
