# Supplementary figures and images for: Water supply and runoff capture reliability curves for hypothetical rainwater harvesting systems for locations across the U.S. for historical and projected climate conditions (part 1 of 2)
Source: Data Brief. 2018 Mar 11;18:441–7. doi: 10.1016/j.dib.2018.03.024 (PMC5996225; doi:10.1016/j.dib.2018.03.024)

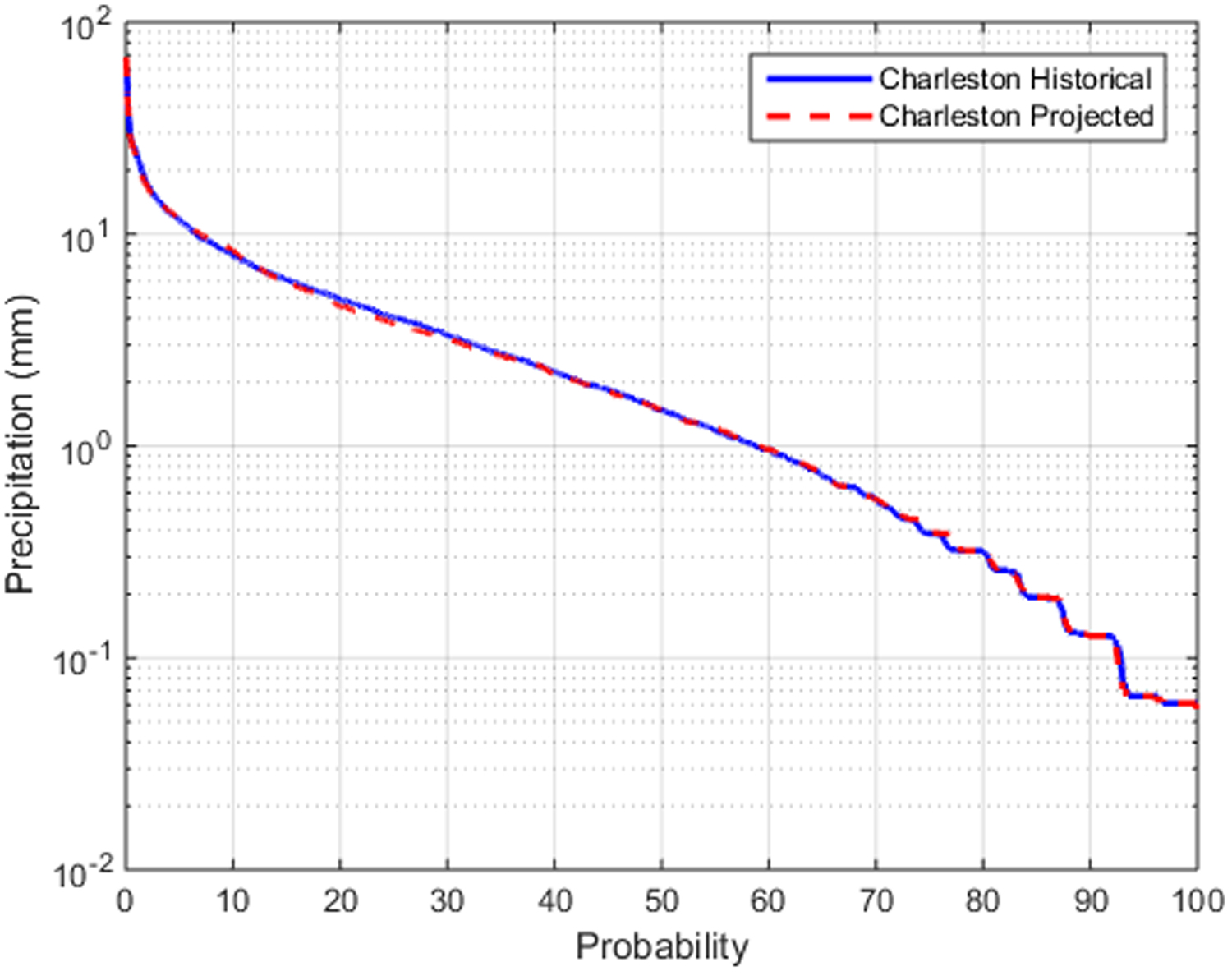

Supplement: Supplementary file 2 — Supplementary material [file mmc2.zip › A1.jpg]

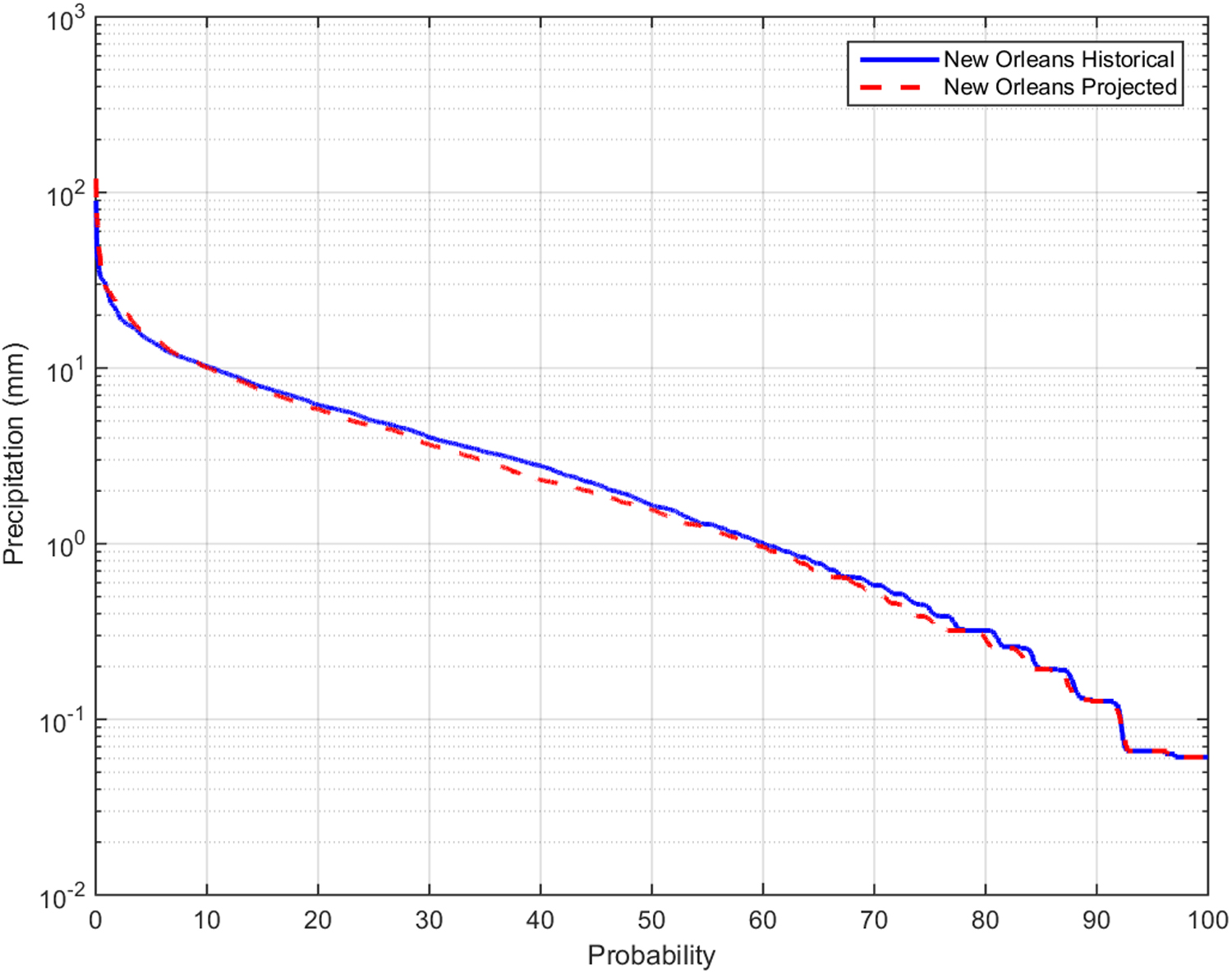

Supplement: Supplementary file 2 — Supplementary material [file mmc2.zip › A10.jpg]

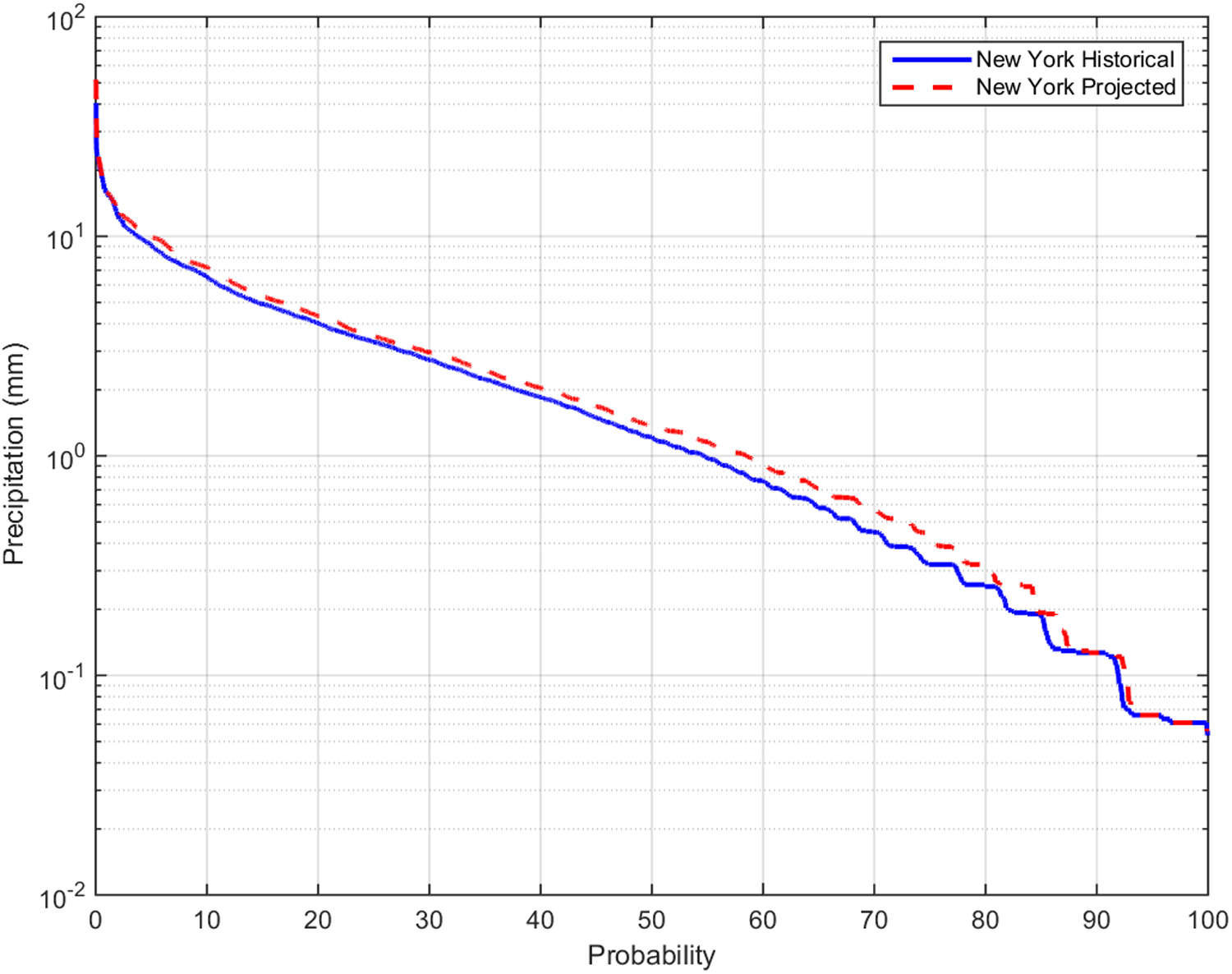

Supplement: Supplementary file 2 — Supplementary material [file mmc2.zip › A11.jpg]

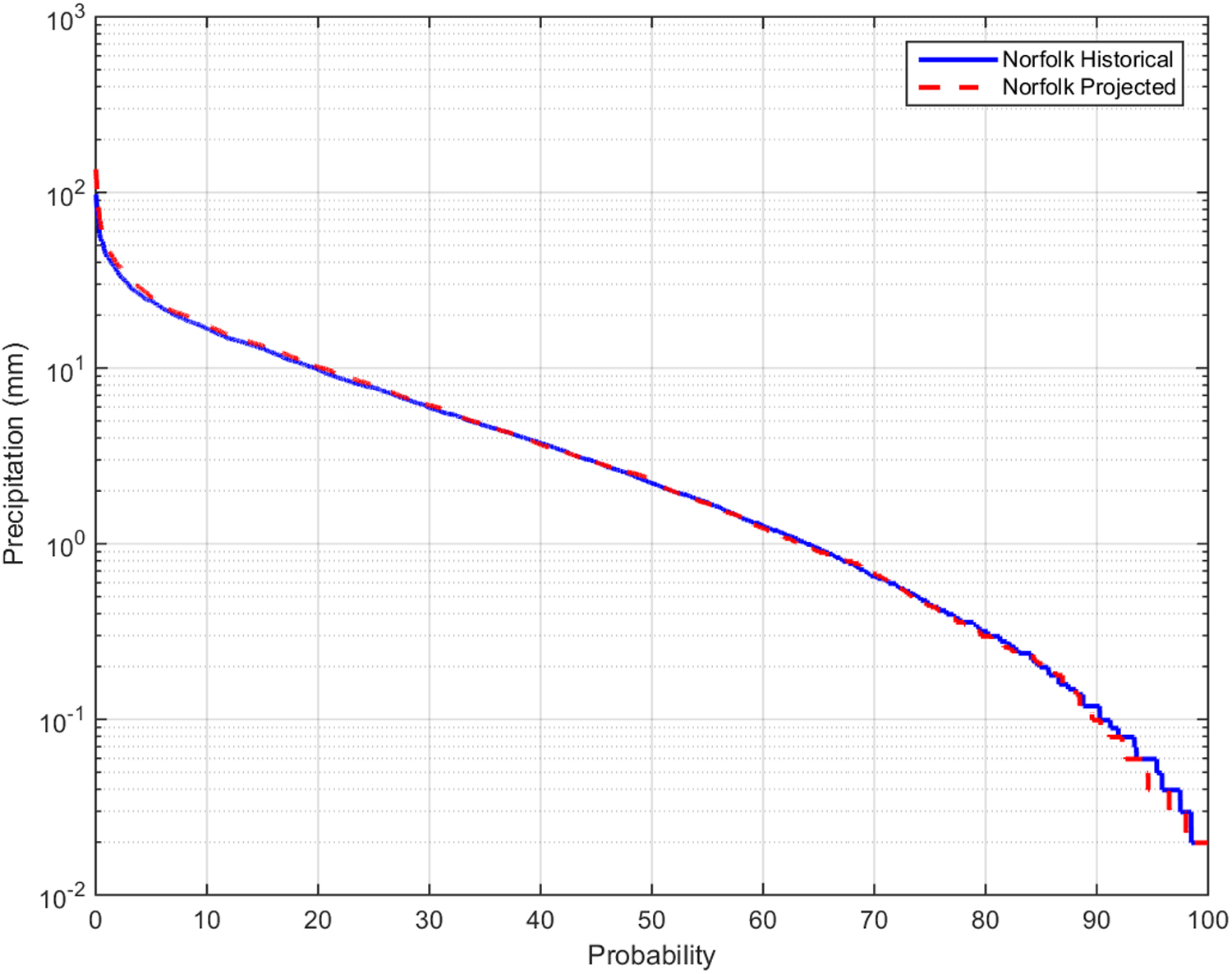

Supplement: Supplementary file 2 — Supplementary material [file mmc2.zip › A12.jpg]

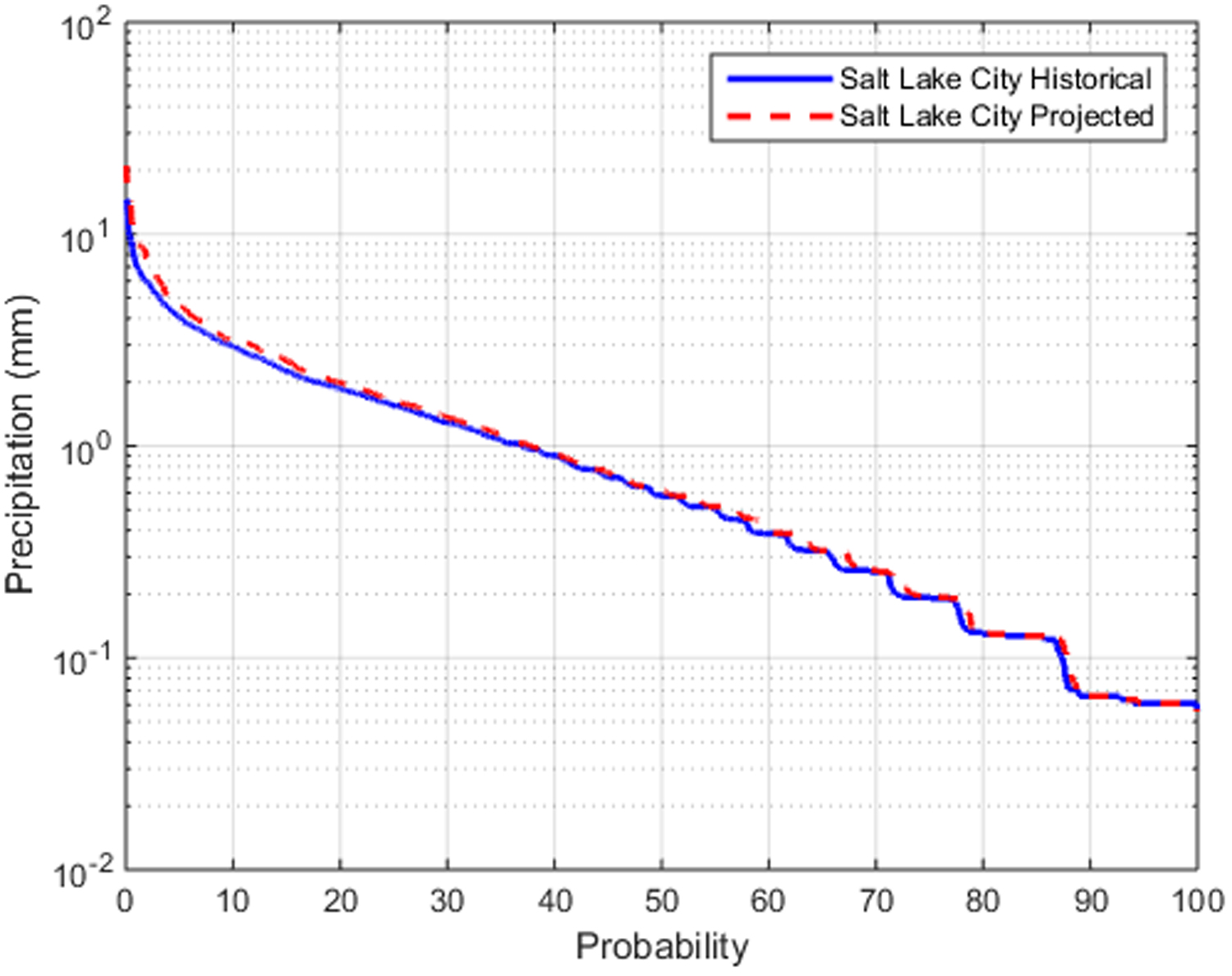

Supplement: Supplementary file 2 — Supplementary material [file mmc2.zip › A13.jpg]

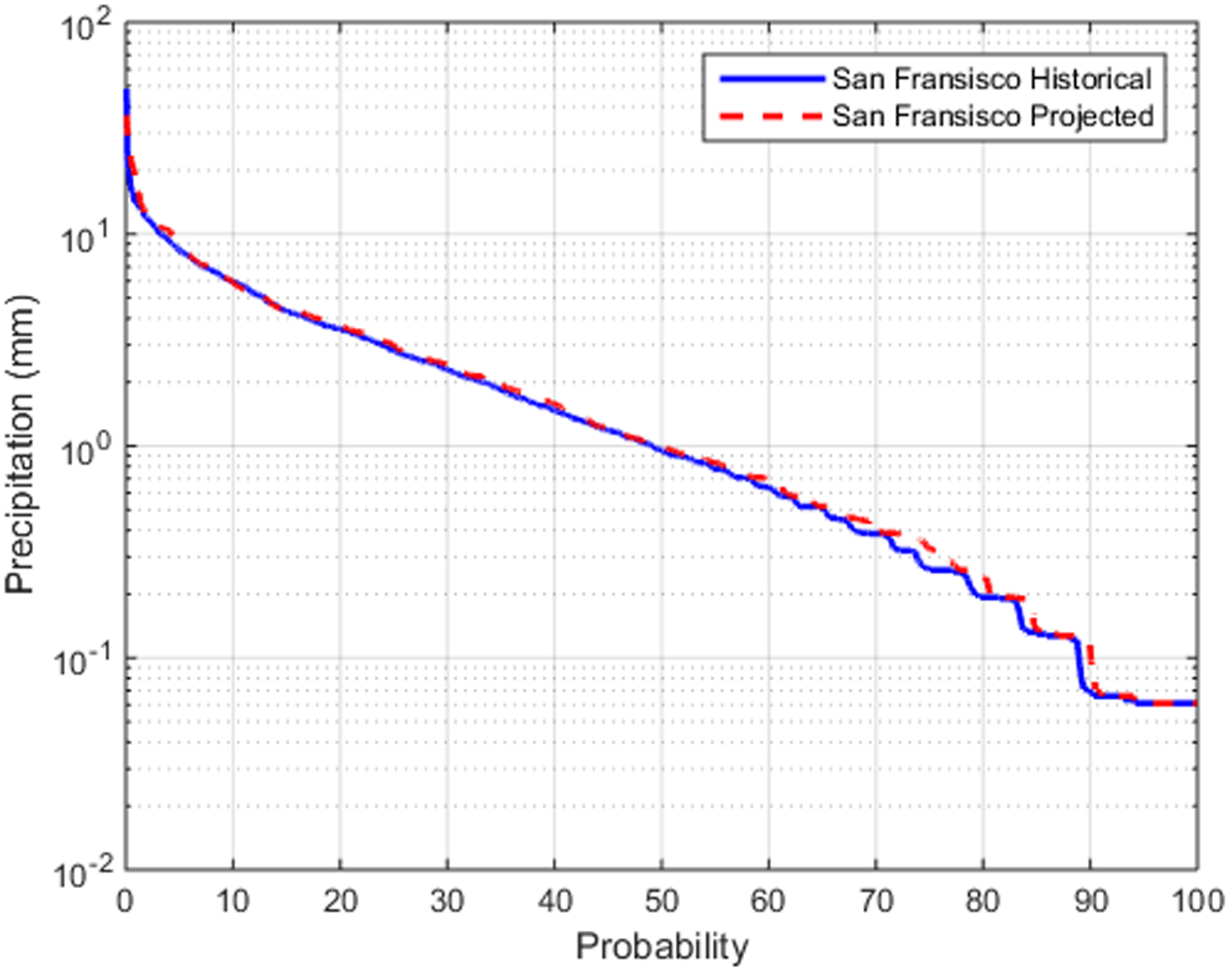

Supplement: Supplementary file 2 — Supplementary material [file mmc2.zip › A14.jpg]

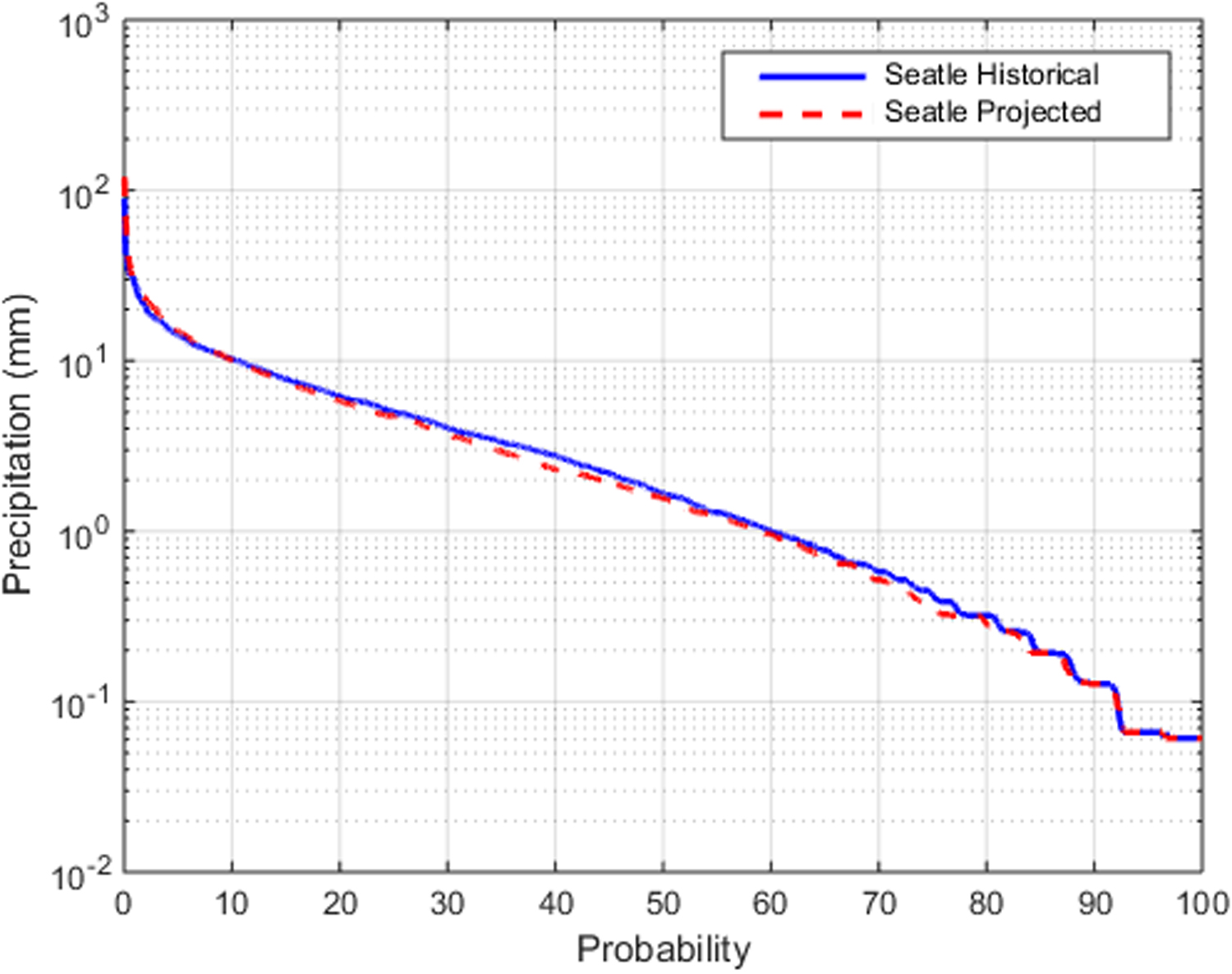

Supplement: Supplementary file 2 — Supplementary material [file mmc2.zip › A15.jpg]

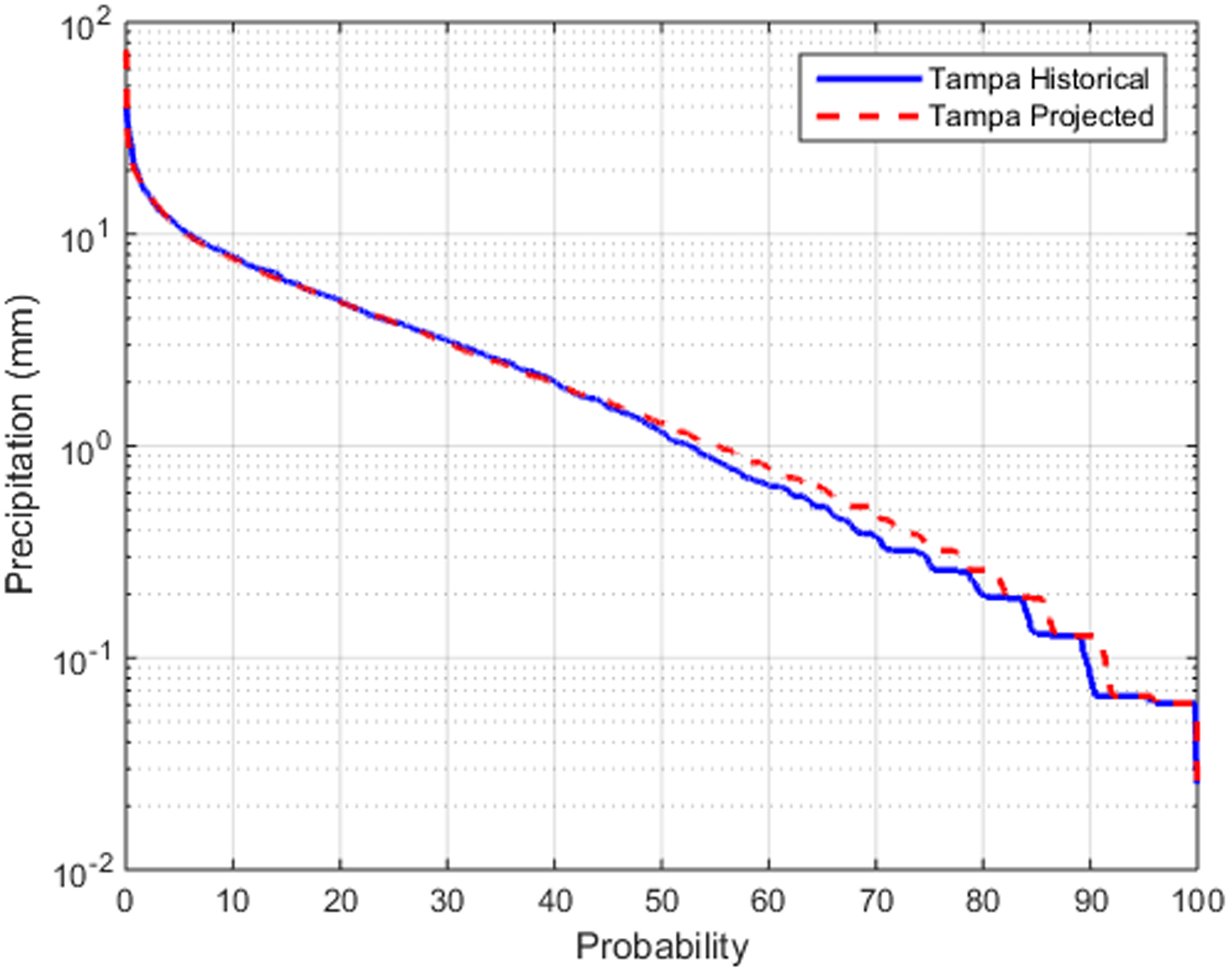

Supplement: Supplementary file 2 — Supplementary material [file mmc2.zip › A16.jpg]

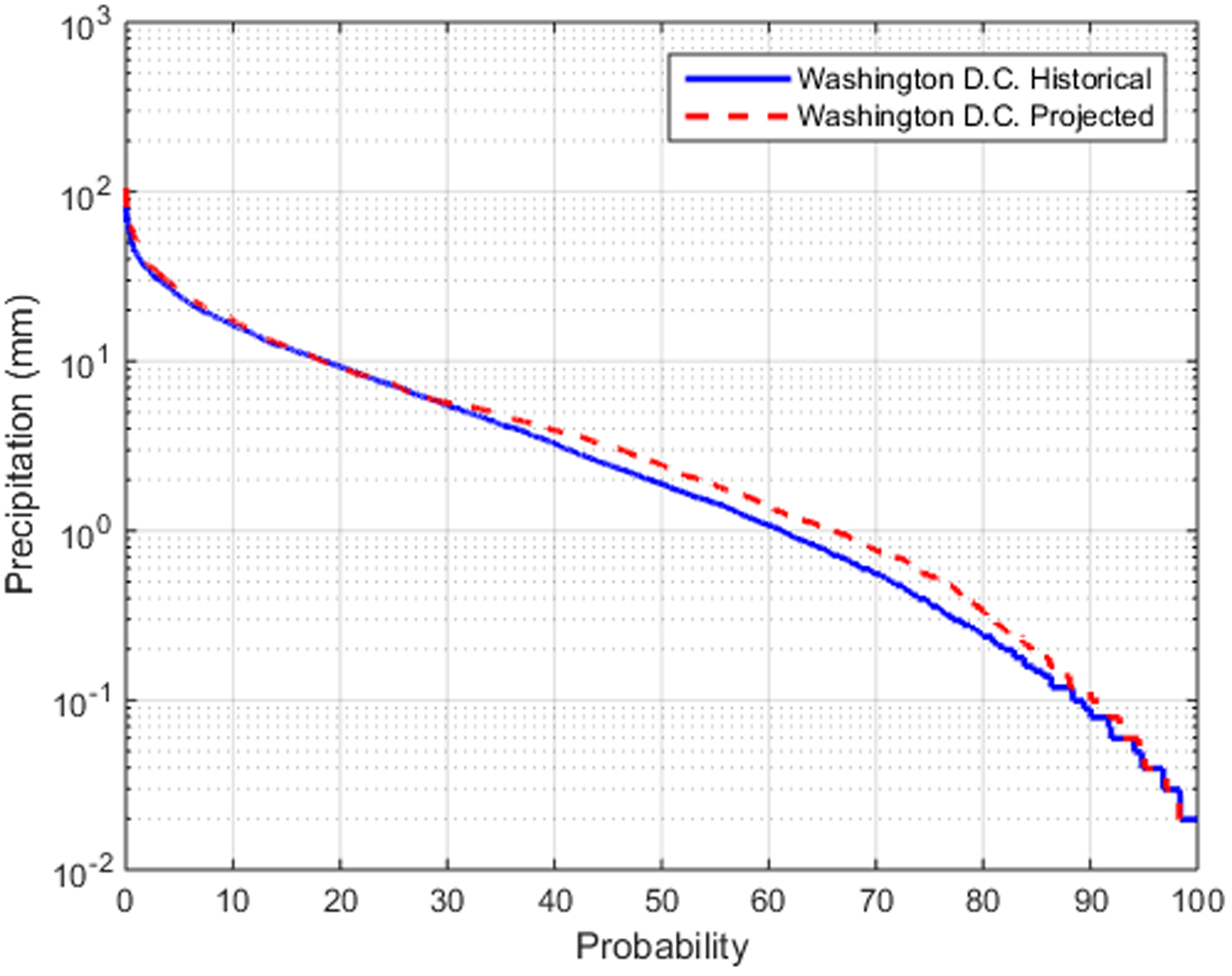

Supplement: Supplementary file 2 — Supplementary material [file mmc2.zip › A17.jpg]

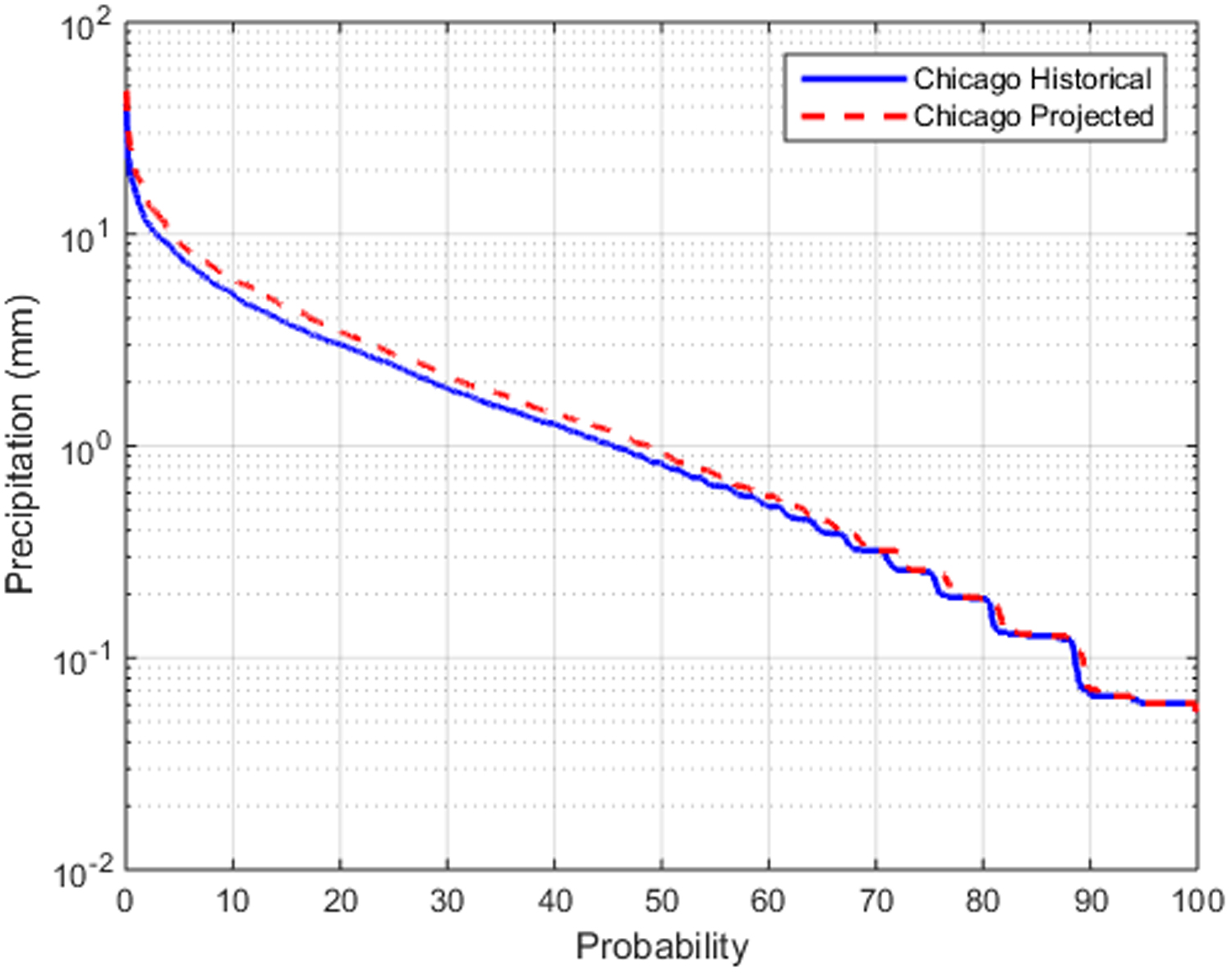

Supplement: Supplementary file 2 — Supplementary material [file mmc2.zip › A2.jpg]

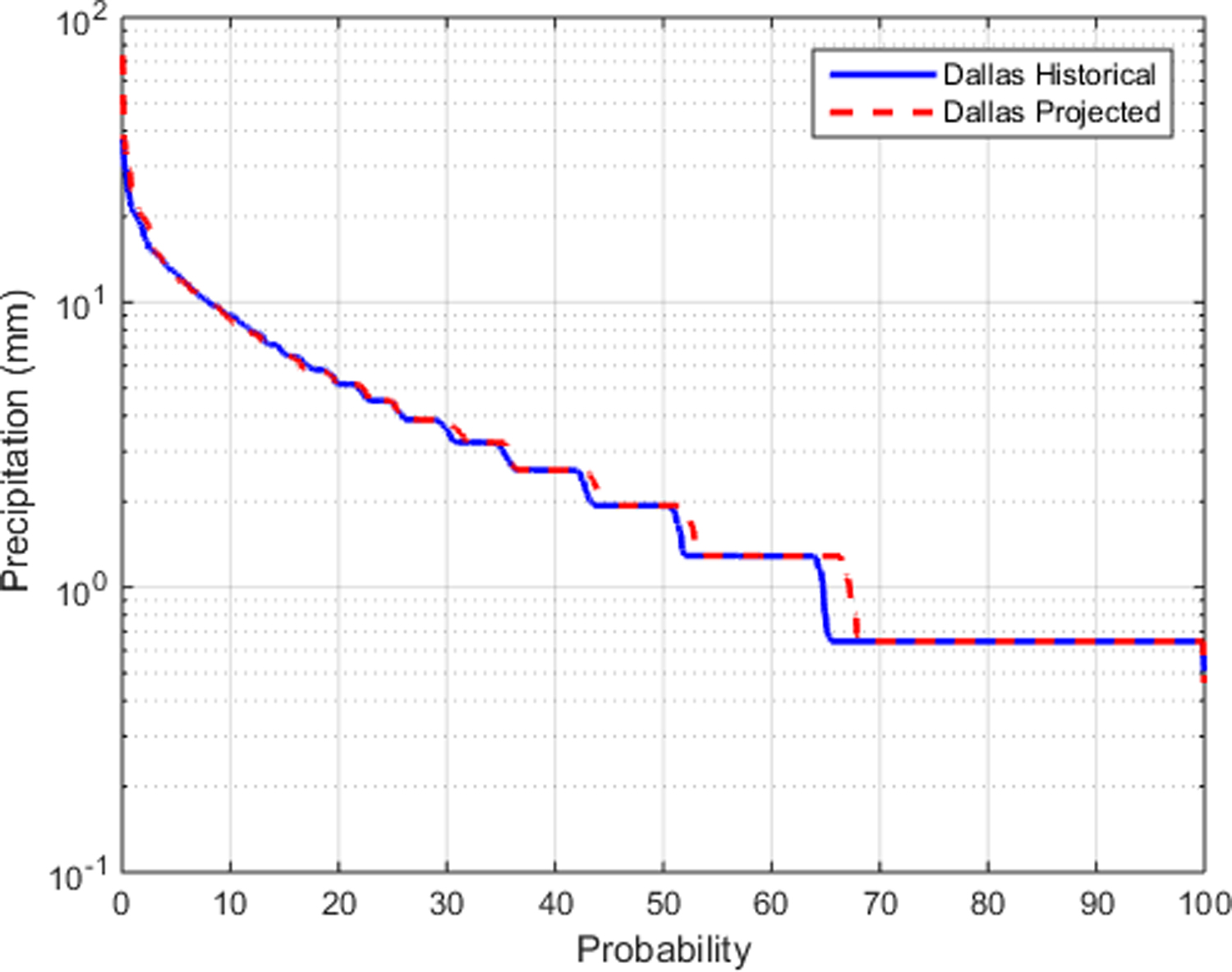

Supplement: Supplementary file 2 — Supplementary material [file mmc2.zip › A3.jpg]

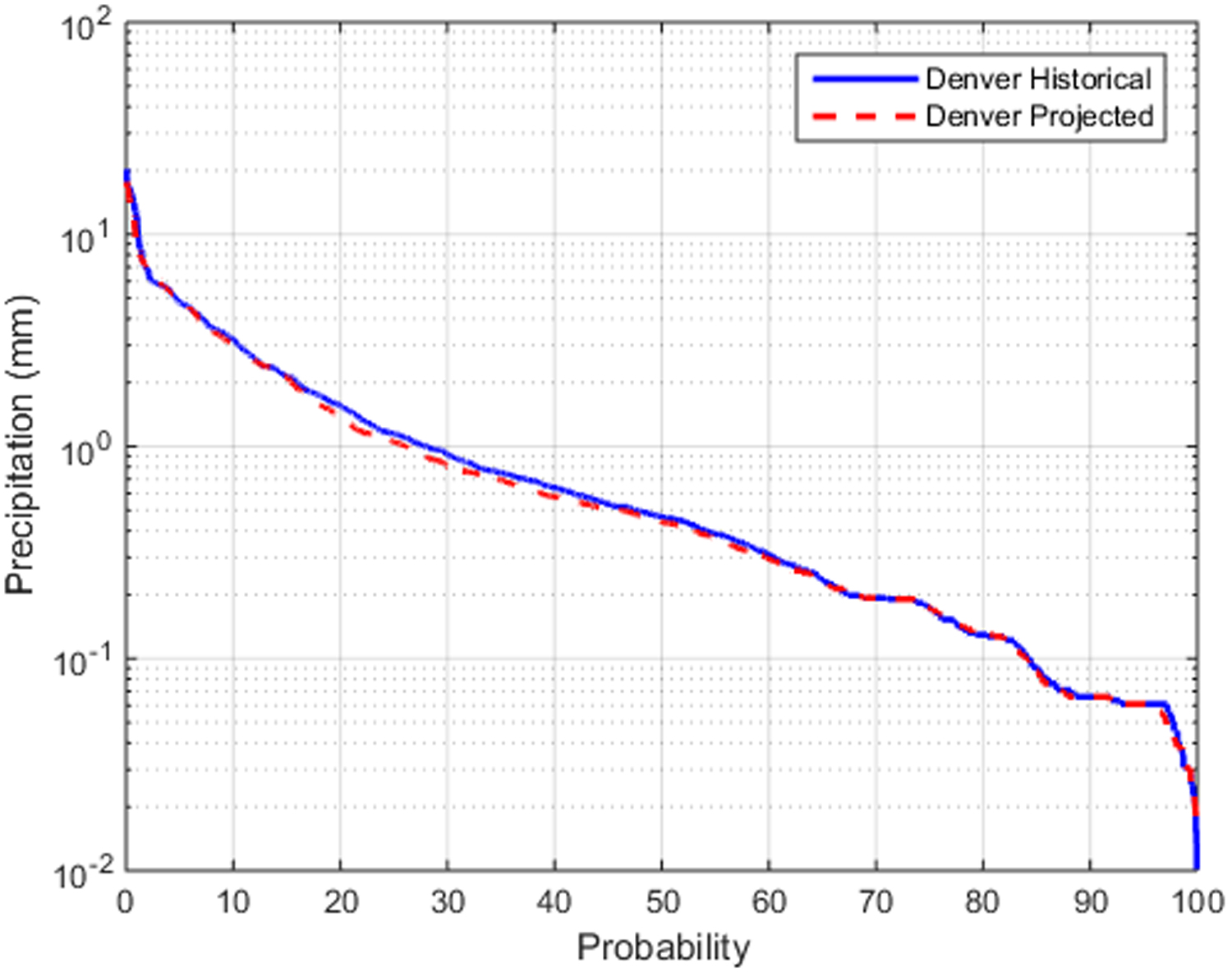

Supplement: Supplementary file 2 — Supplementary material [file mmc2.zip › A4.jpg]

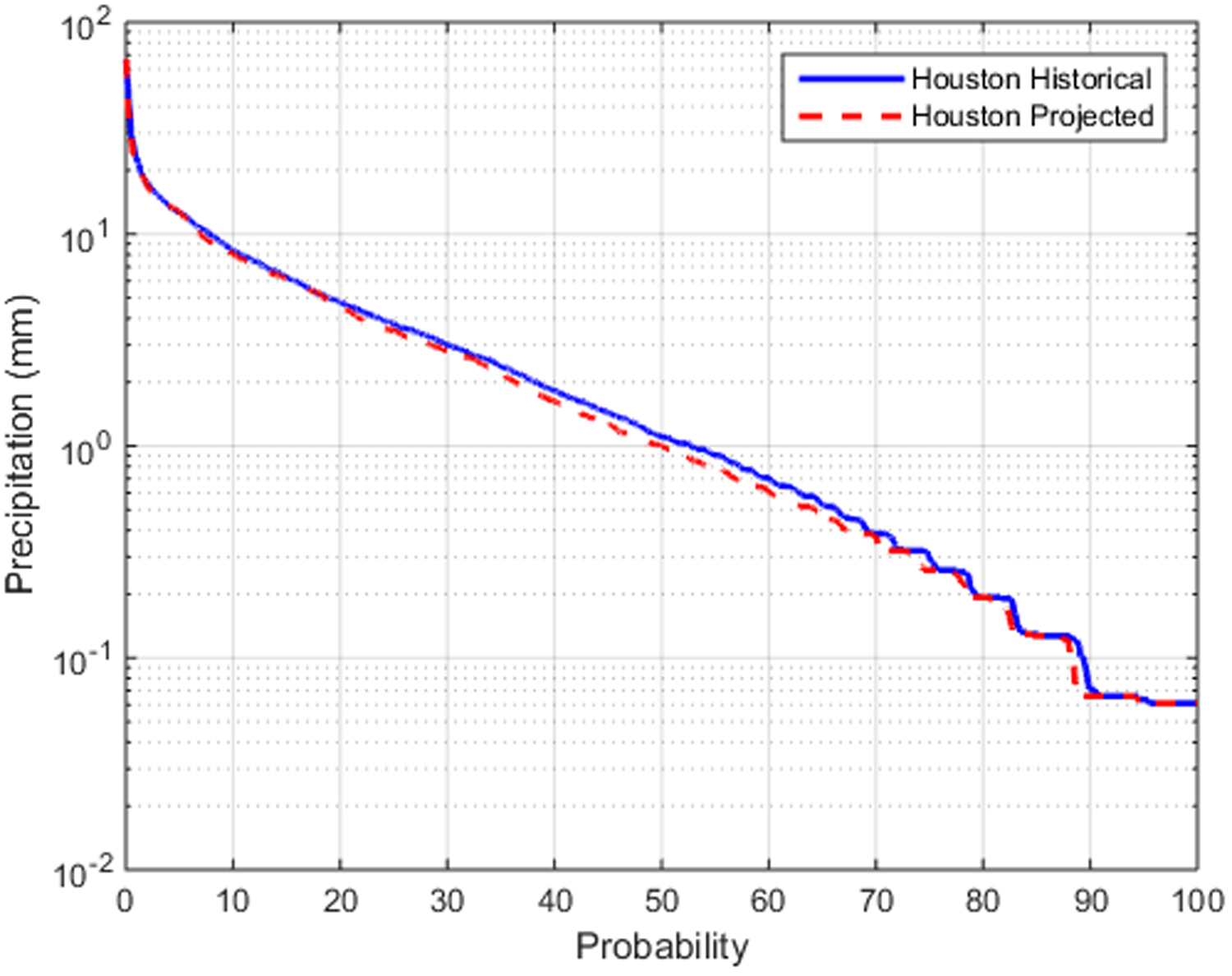

Supplement: Supplementary file 2 — Supplementary material [file mmc2.zip › A5.jpg]

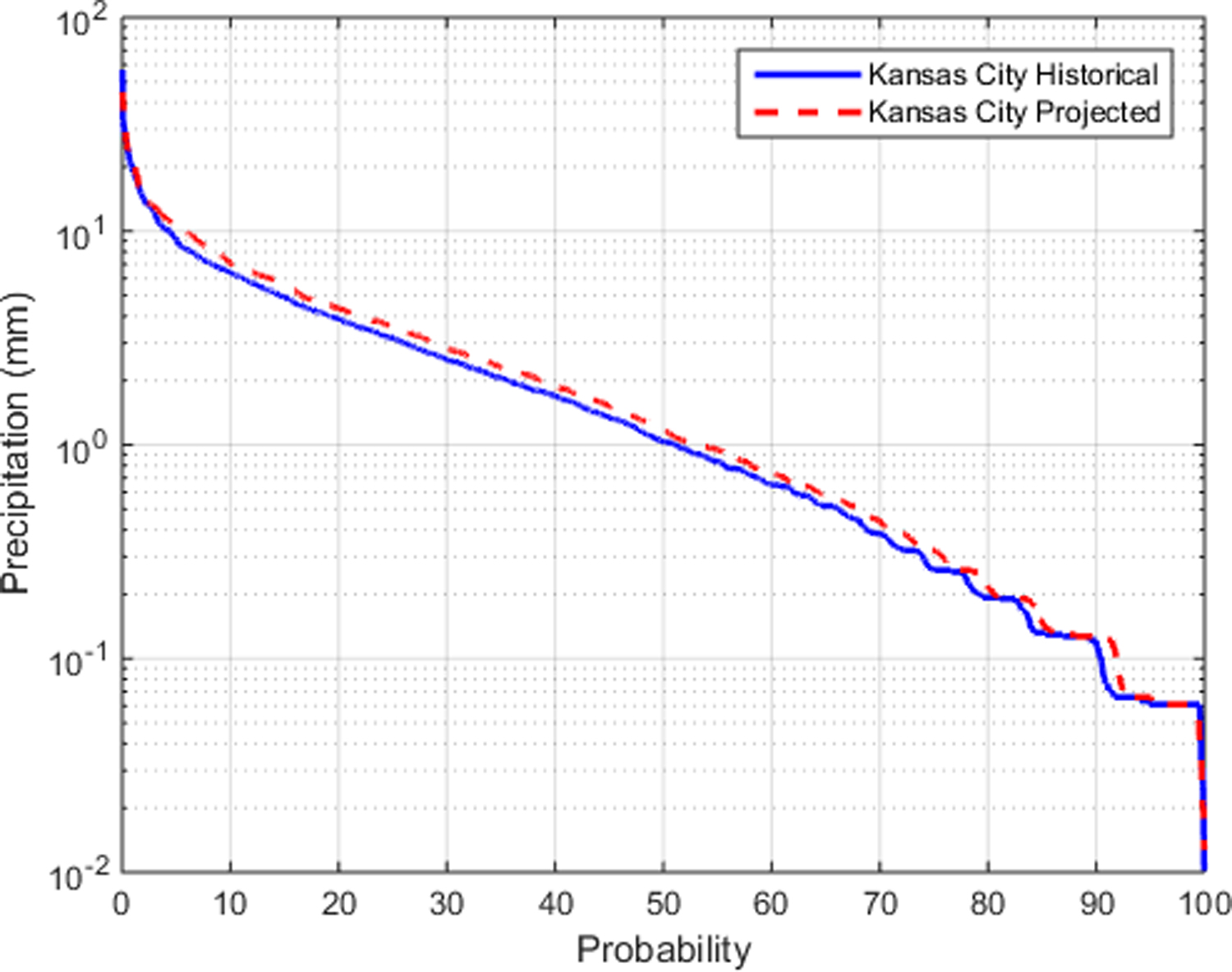

Supplement: Supplementary file 2 — Supplementary material [file mmc2.zip › A6.jpg]

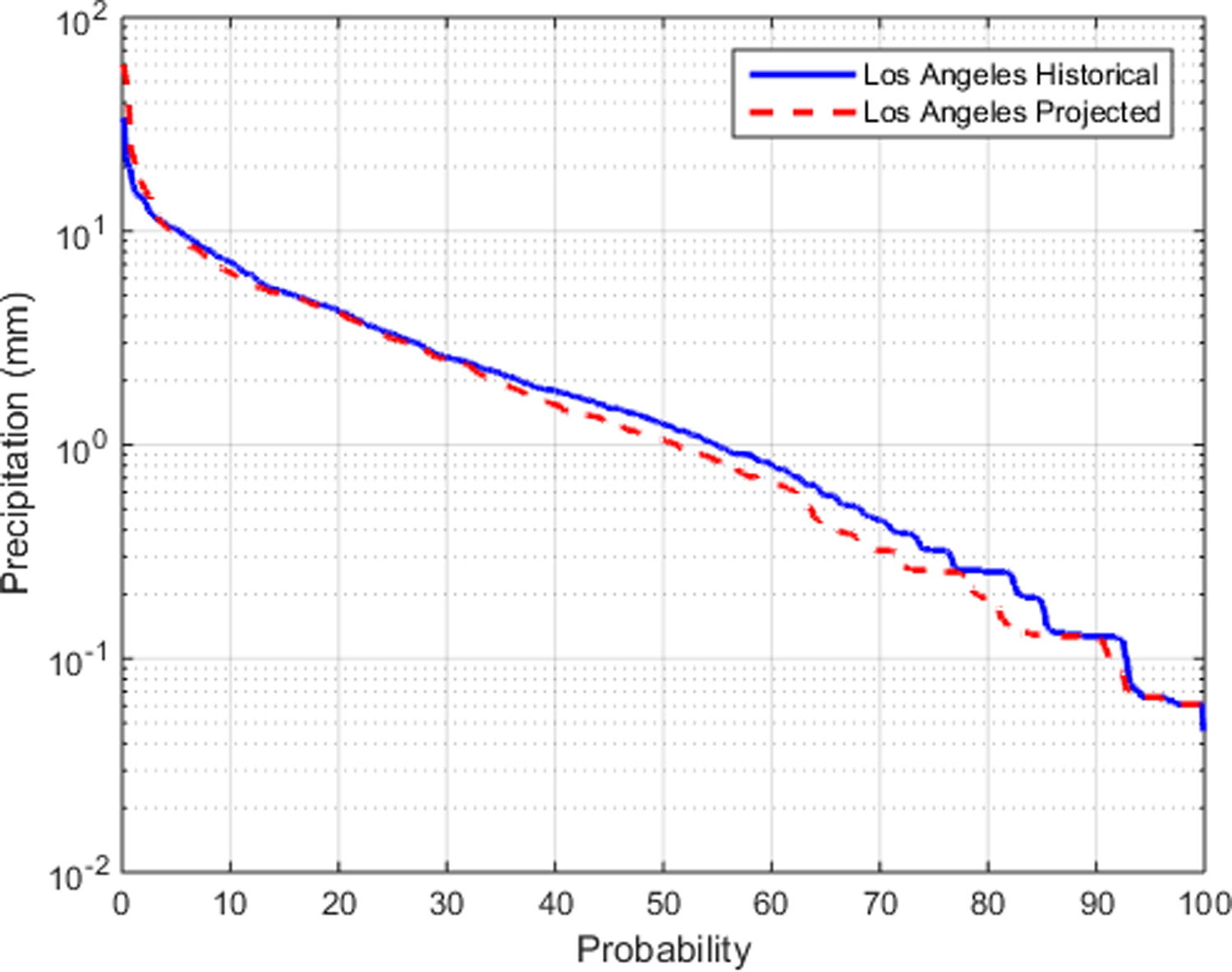

Supplement: Supplementary file 2 — Supplementary material [file mmc2.zip › A7.jpg]

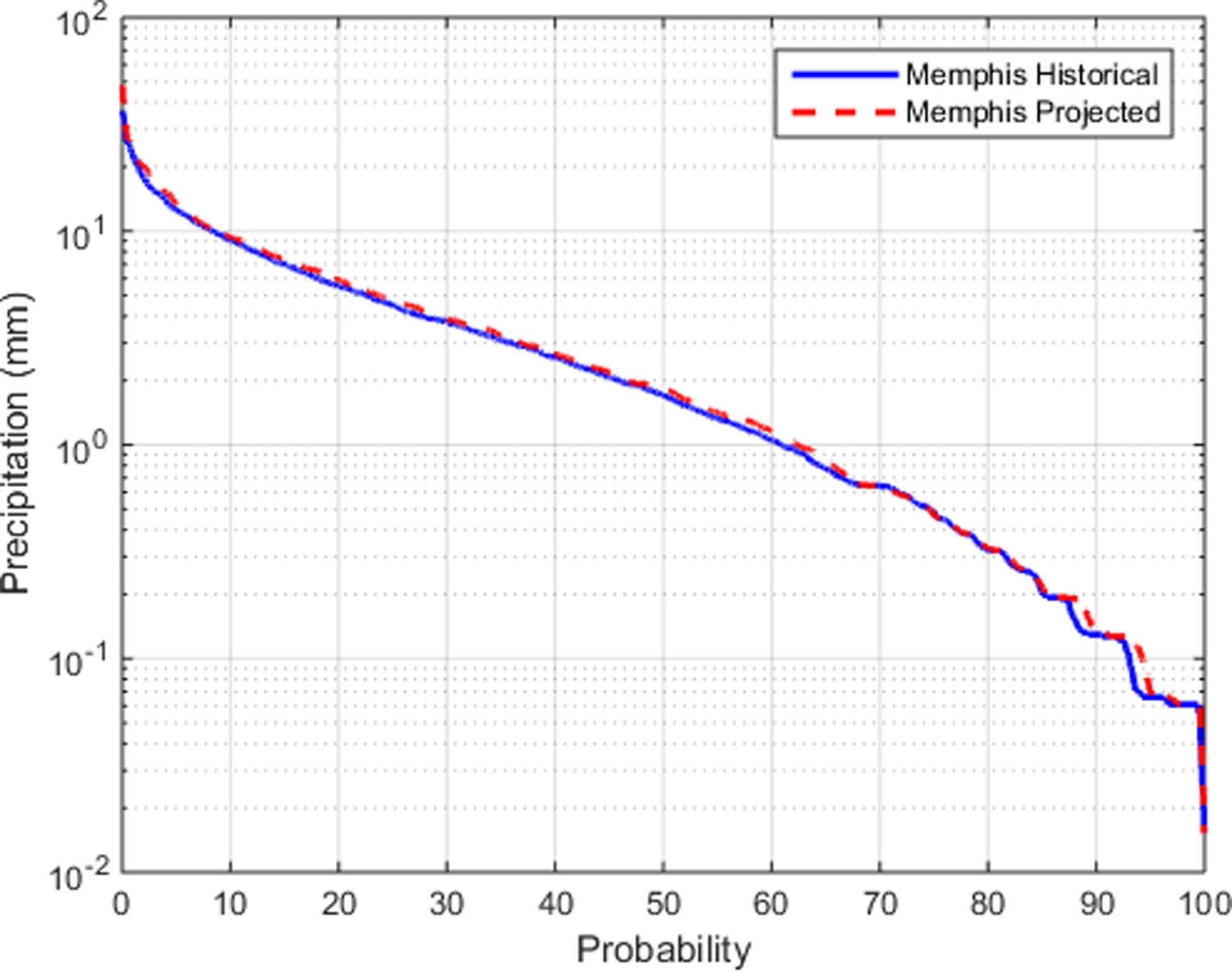

Supplement: Supplementary file 2 — Supplementary material [file mmc2.zip › A8.jpg]

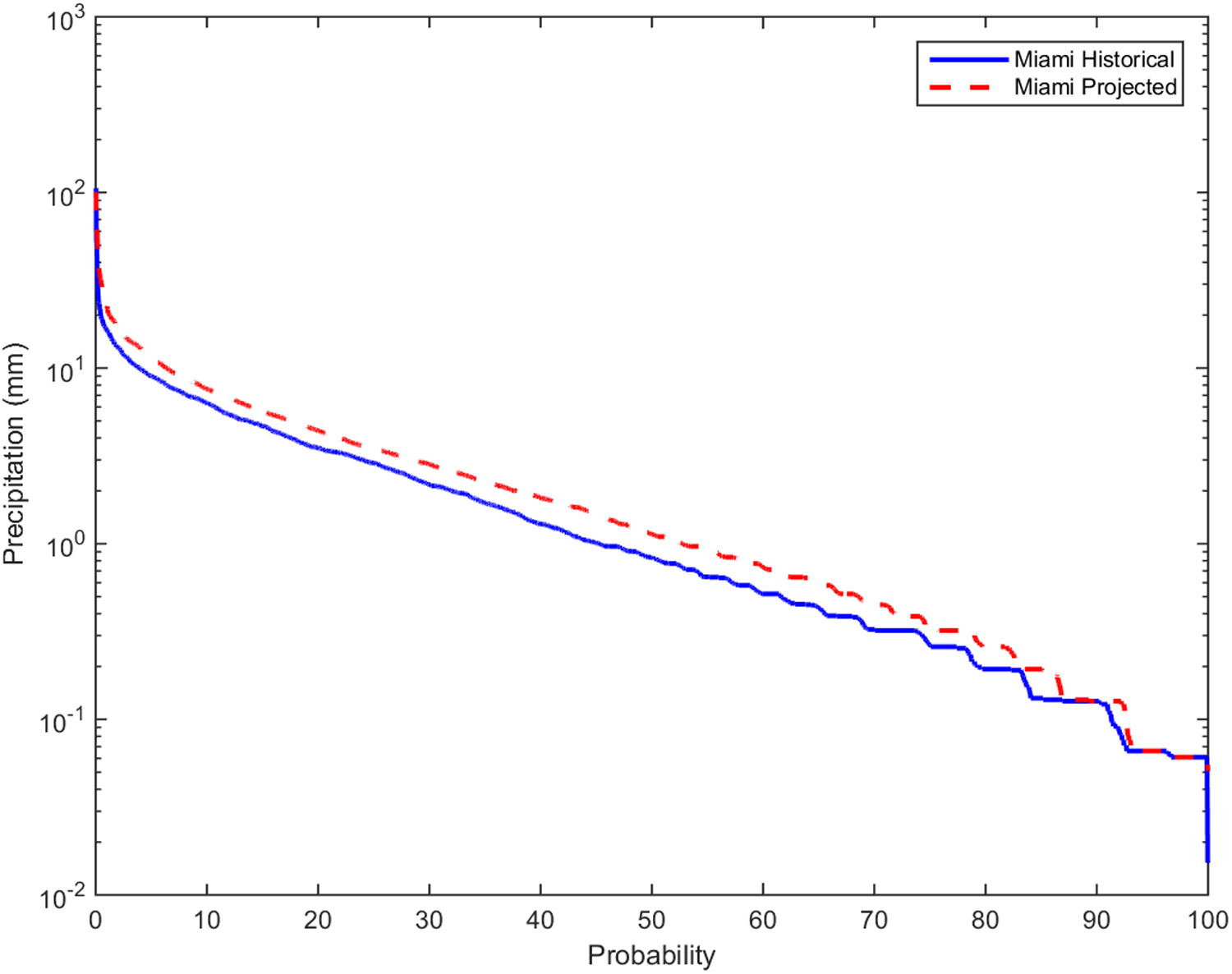

Supplement: Supplementary file 2 — Supplementary material [file mmc2.zip › A9.jpg]

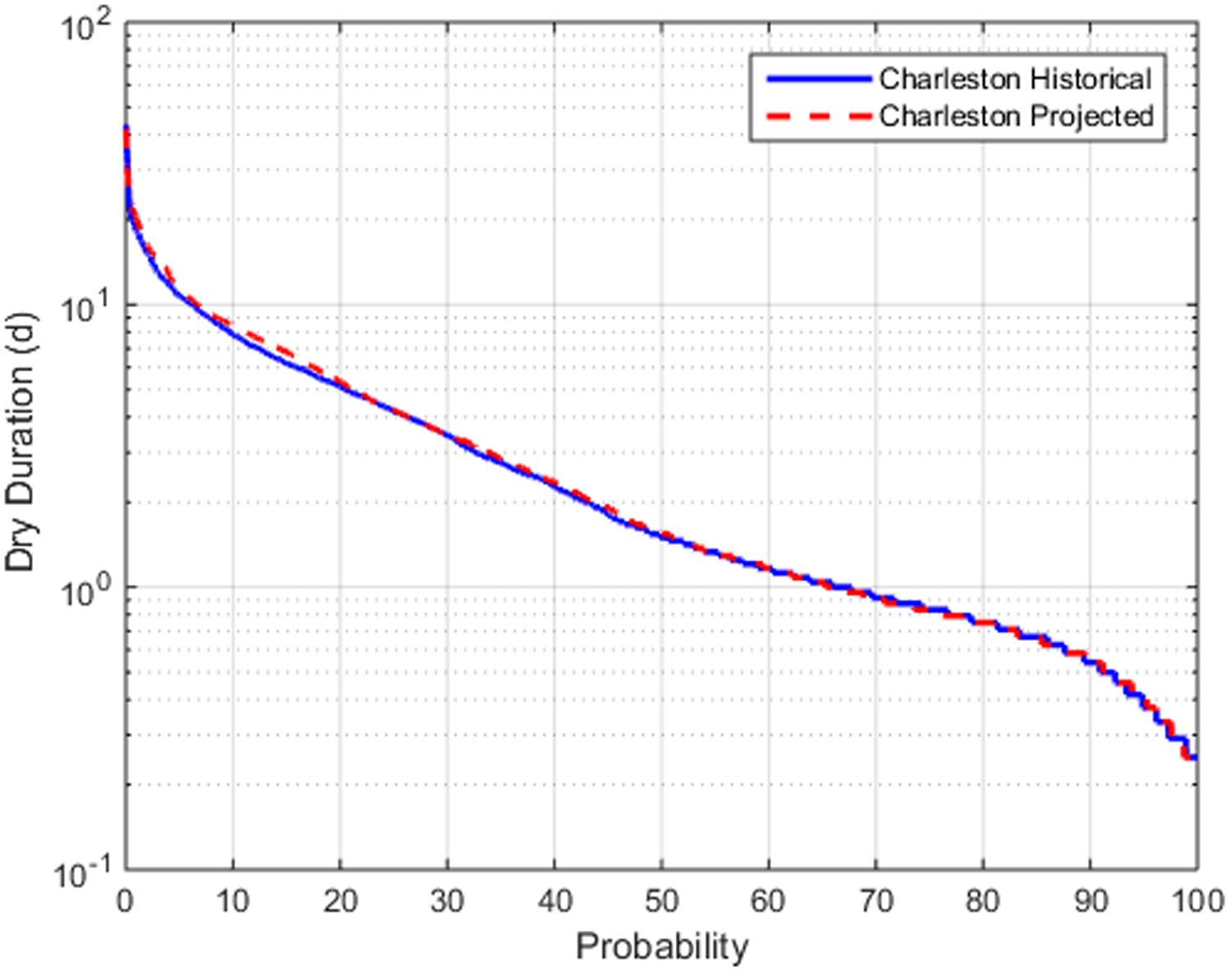

Supplement: Supplementary file 3 — Supplementary material [file mmc3.zip › B1.jpg]

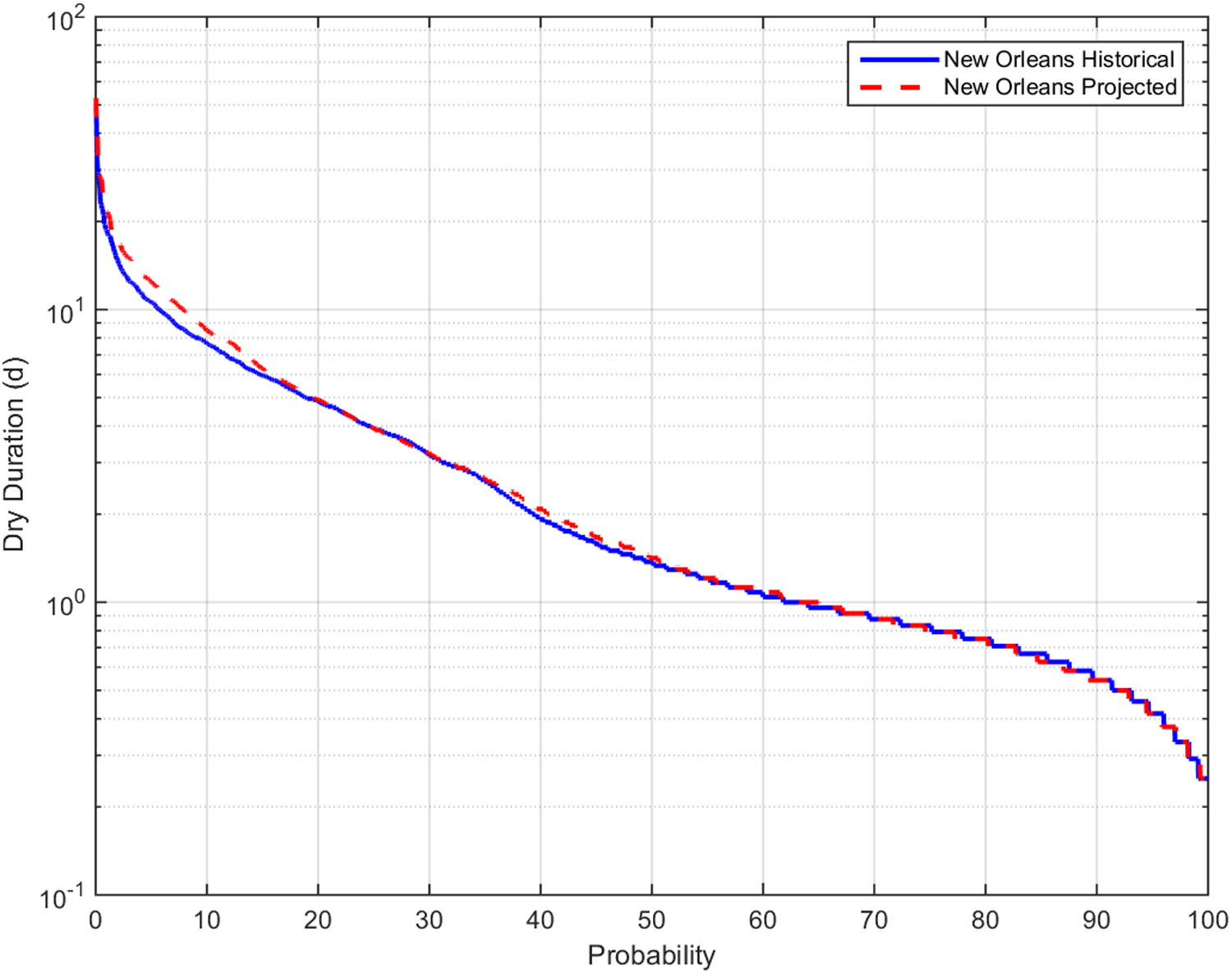

Supplement: Supplementary file 3 — Supplementary material [file mmc3.zip › B10.jpg]

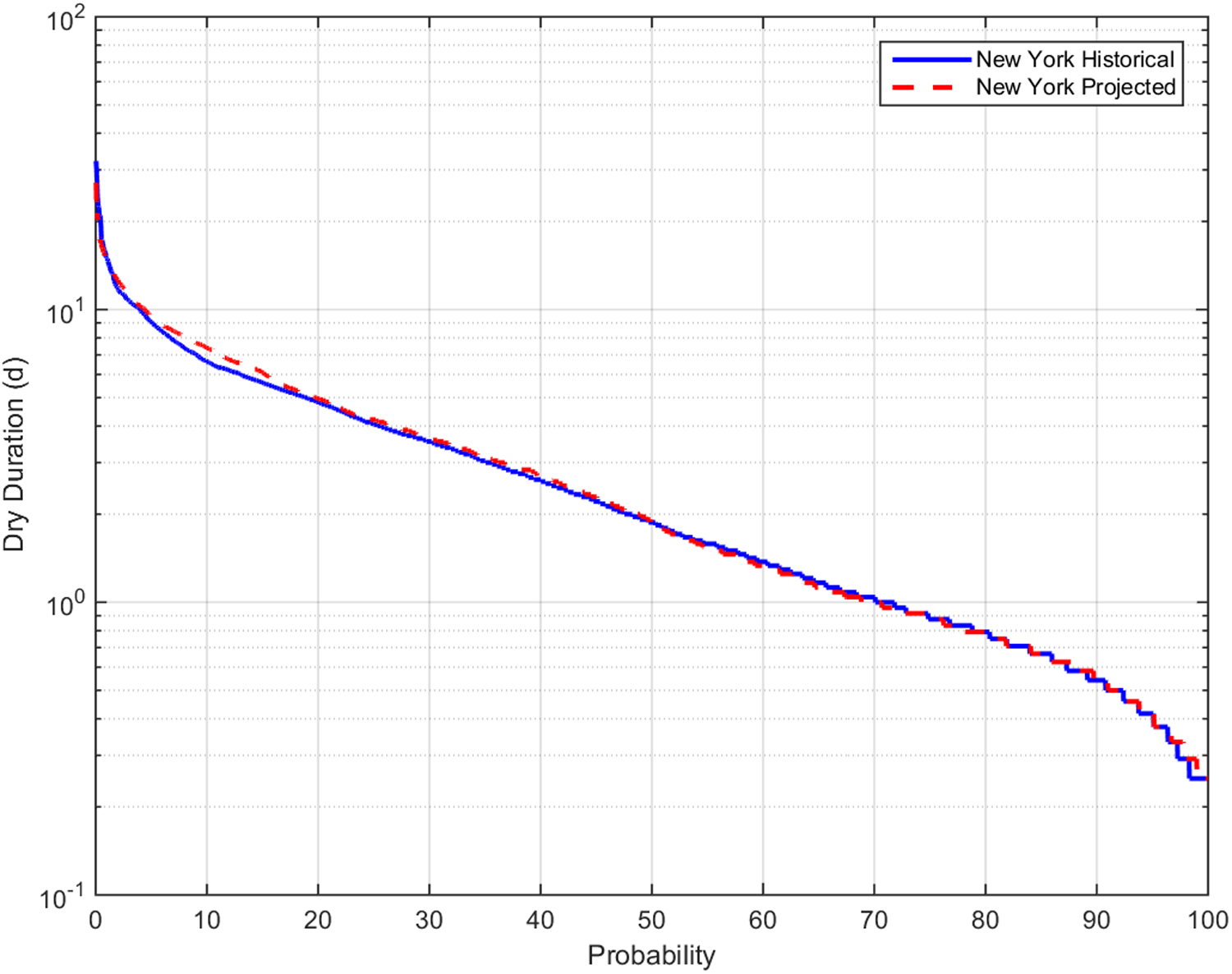

Supplement: Supplementary file 3 — Supplementary material [file mmc3.zip › B11.jpg]

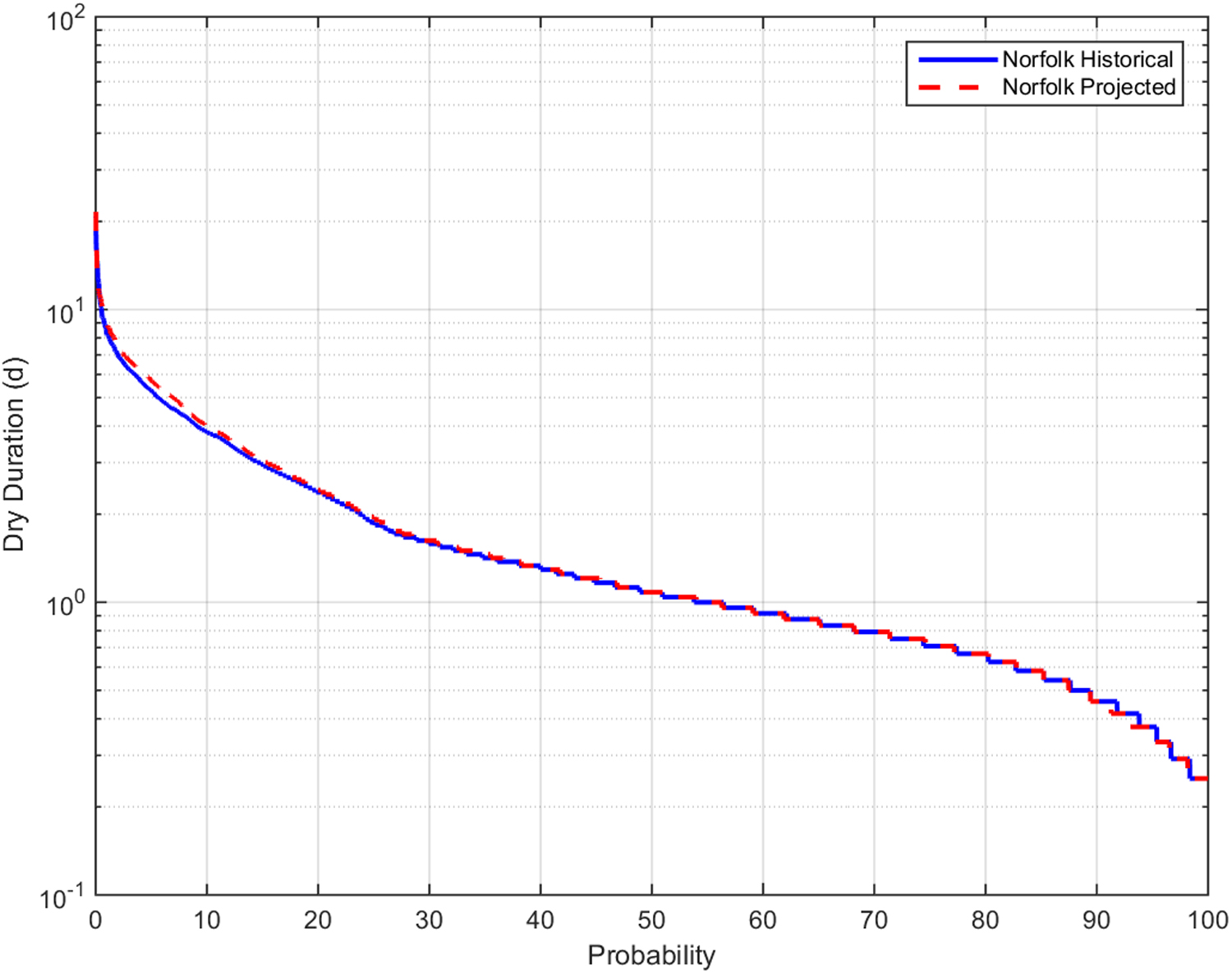

Supplement: Supplementary file 3 — Supplementary material [file mmc3.zip › B12.jpg]

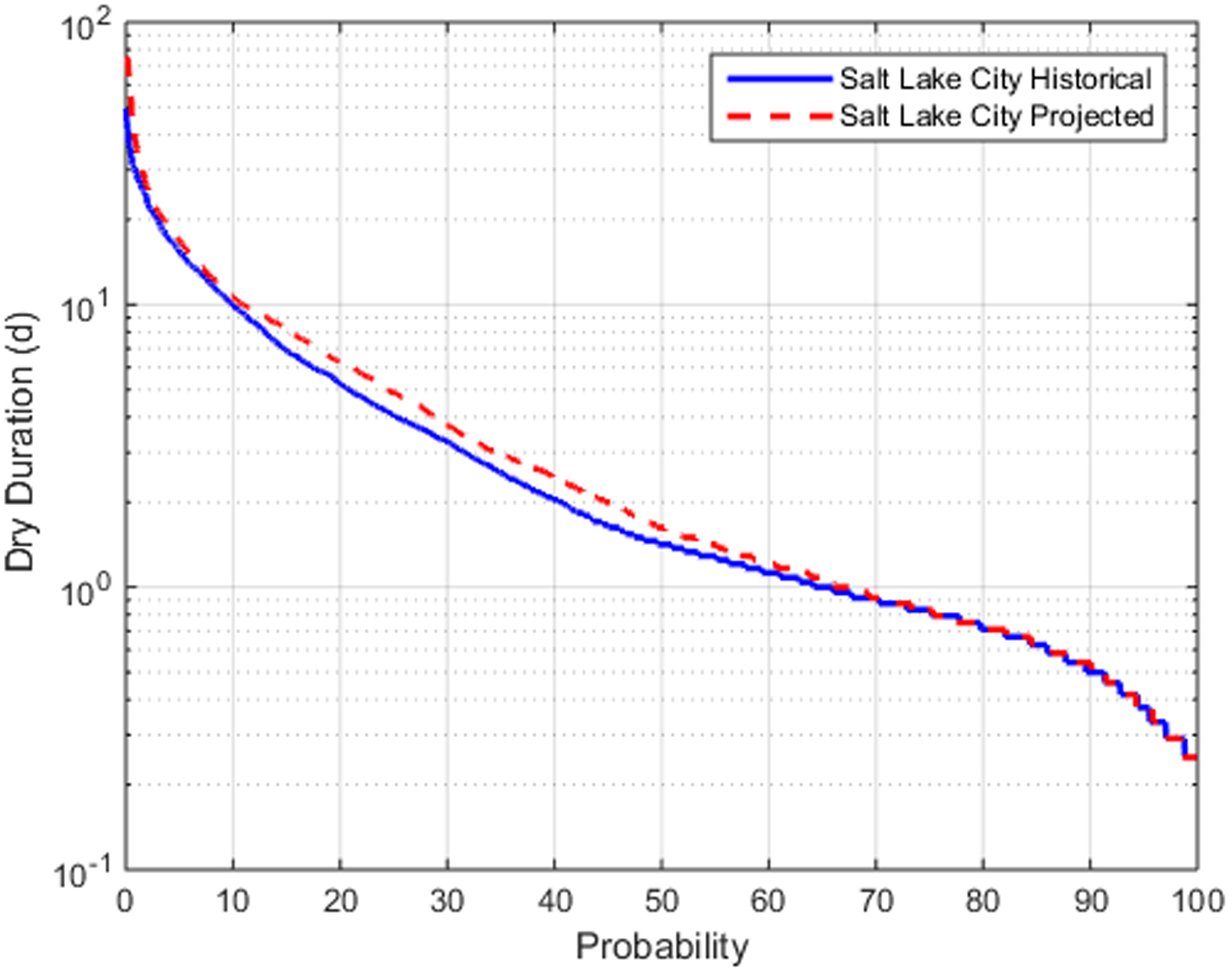

Supplement: Supplementary file 3 — Supplementary material [file mmc3.zip › B13.jpg]

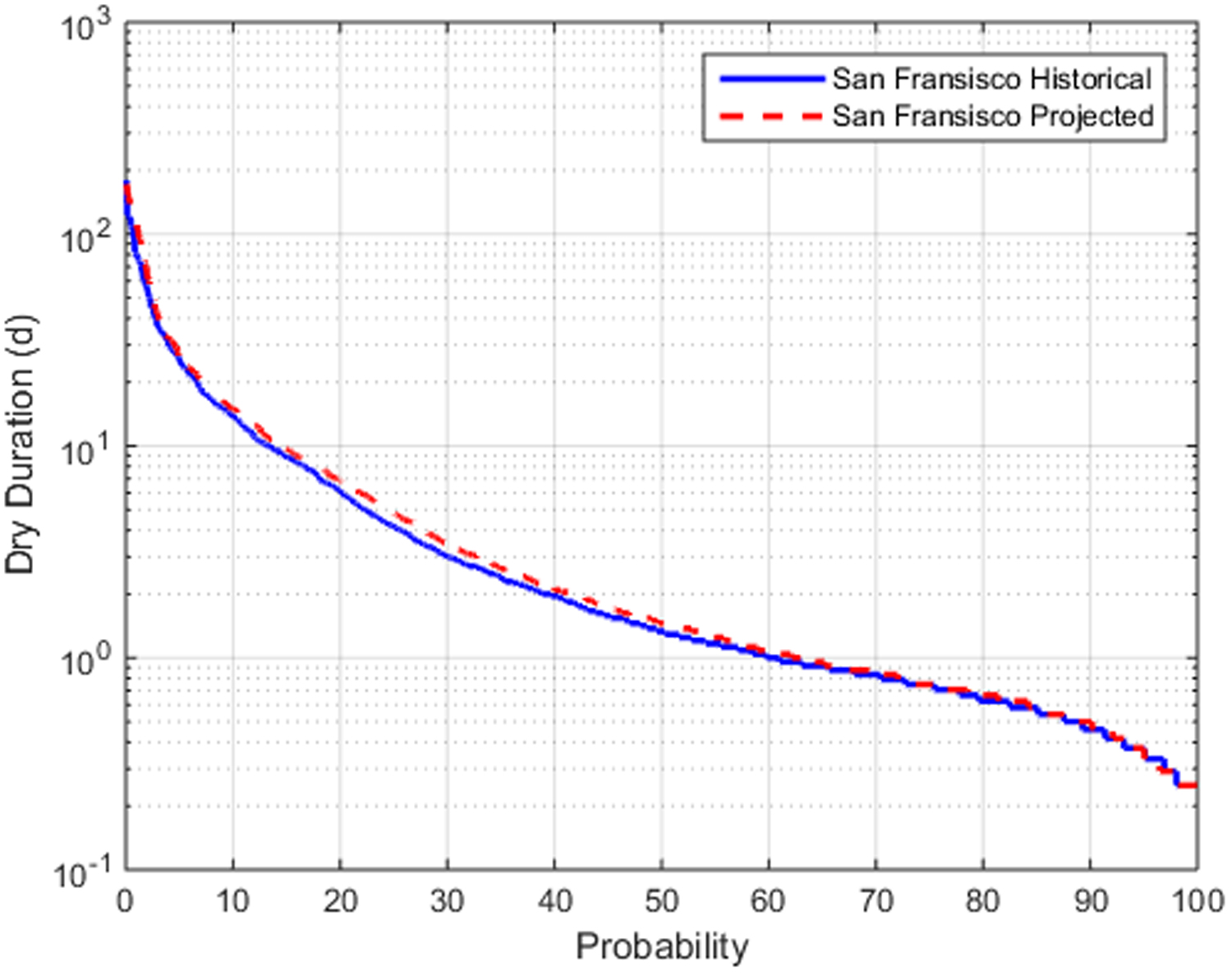

Supplement: Supplementary file 3 — Supplementary material [file mmc3.zip › B14.jpg]

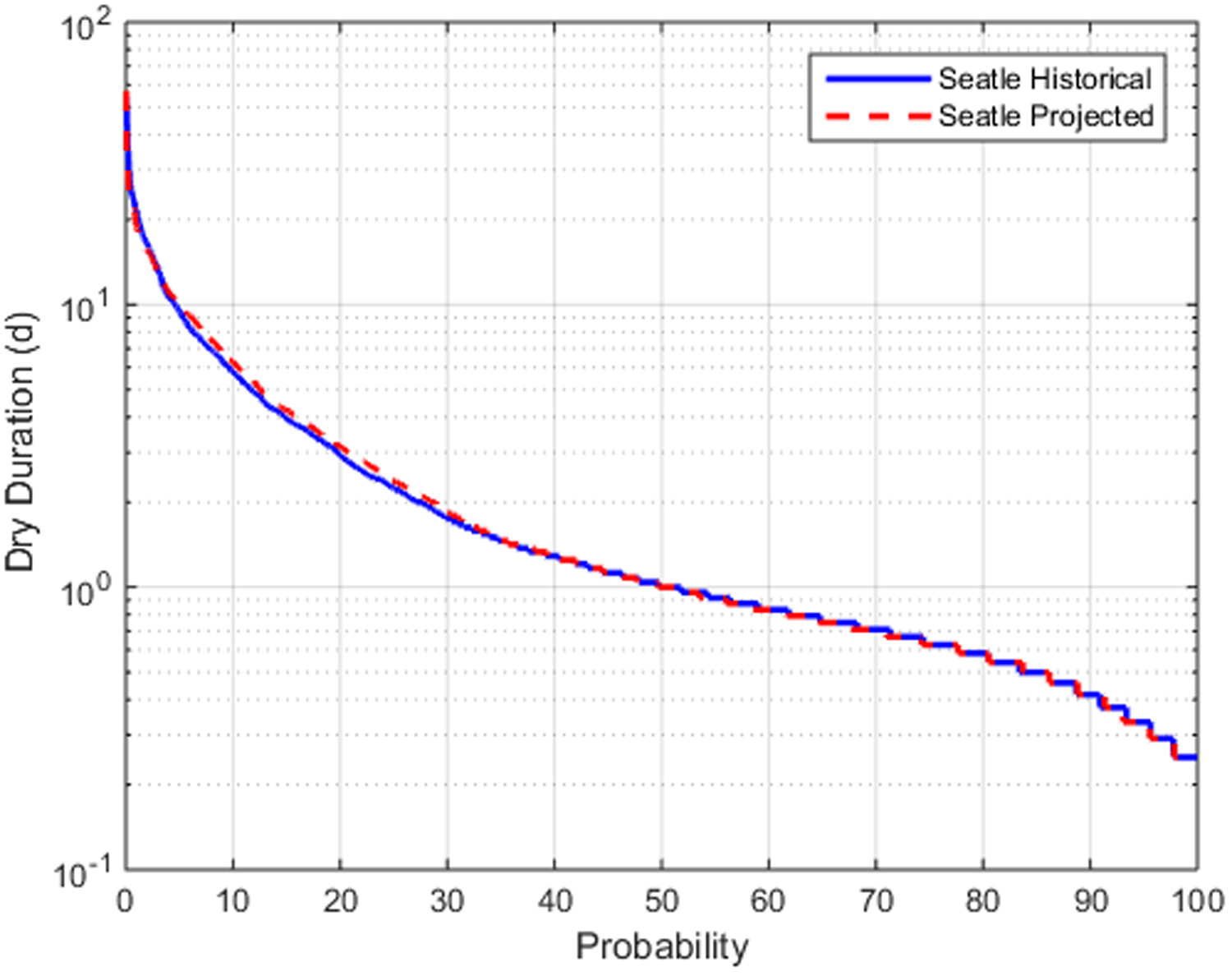

Supplement: Supplementary file 3 — Supplementary material [file mmc3.zip › B15.jpg]

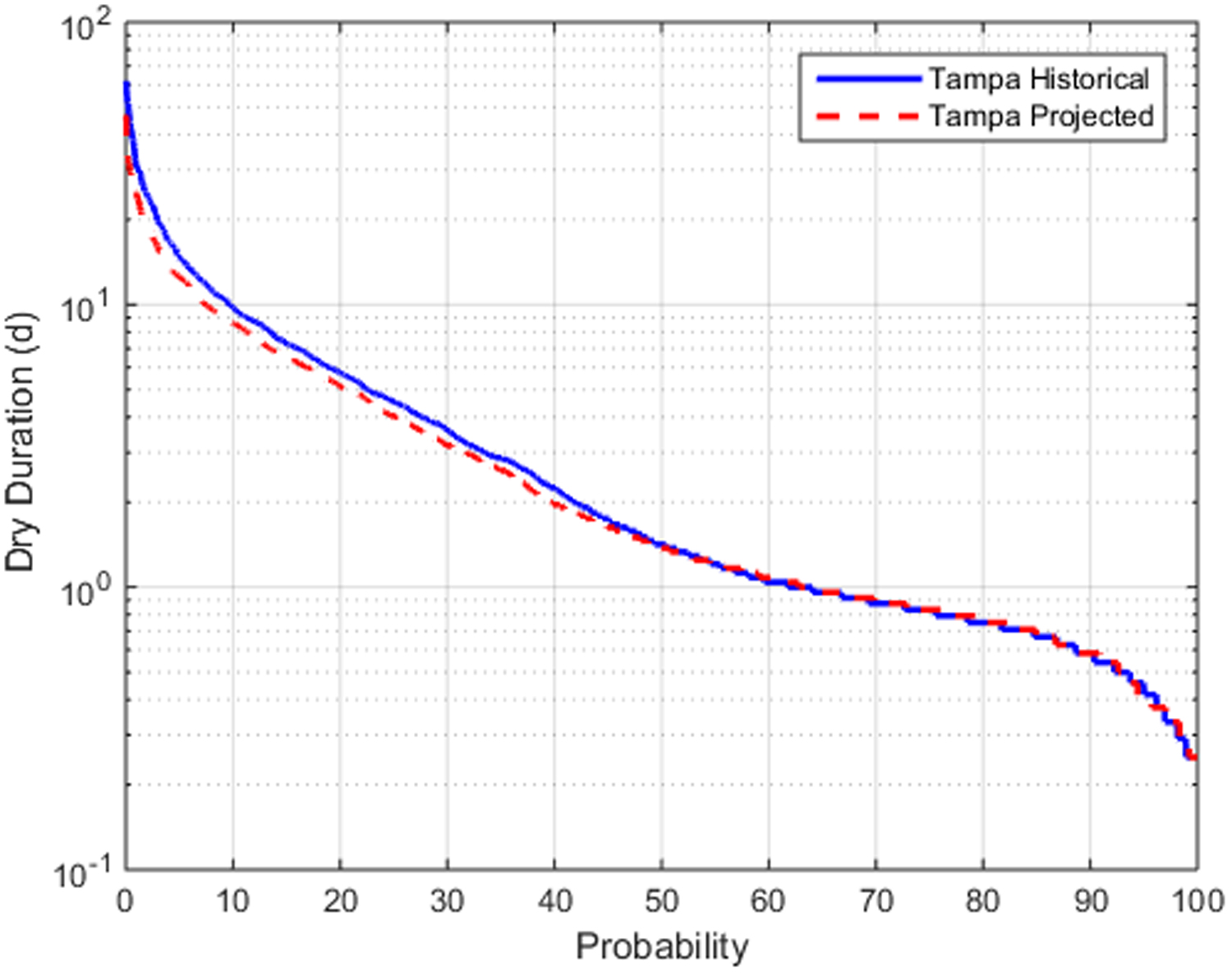

Supplement: Supplementary file 3 — Supplementary material [file mmc3.zip › B16.jpg]

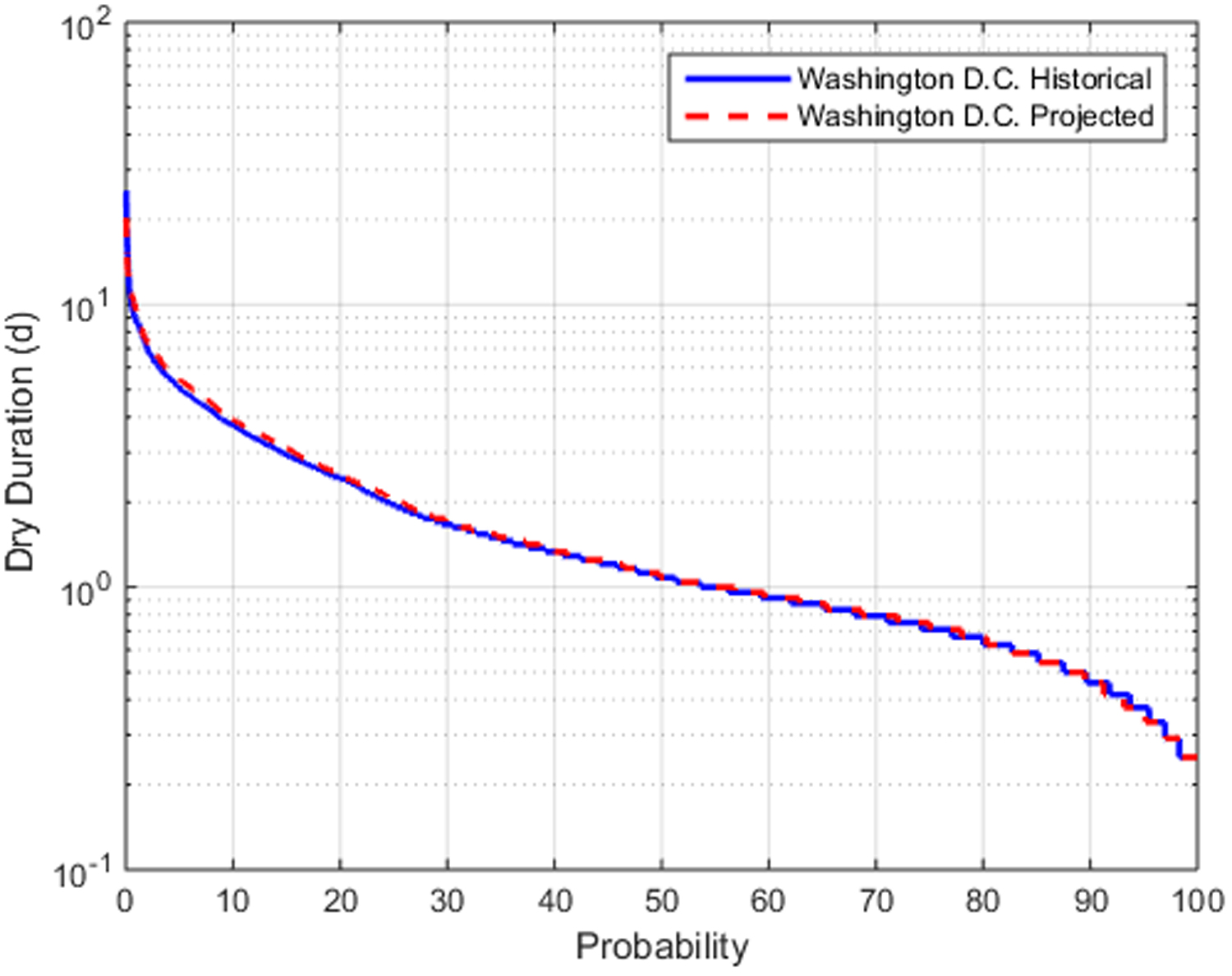

Supplement: Supplementary file 3 — Supplementary material [file mmc3.zip › B17.jpg]

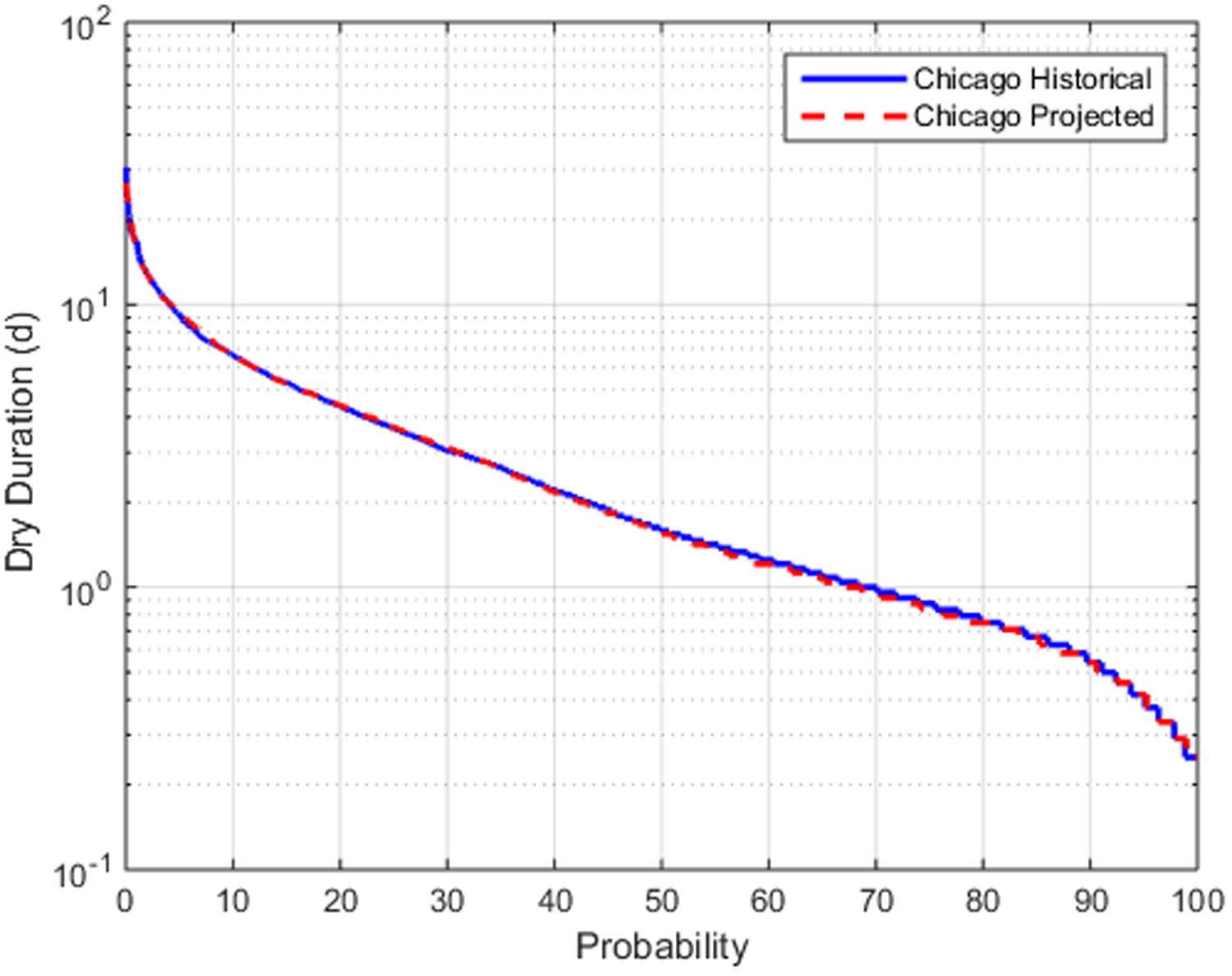

Supplement: Supplementary file 3 — Supplementary material [file mmc3.zip › B2.jpg]

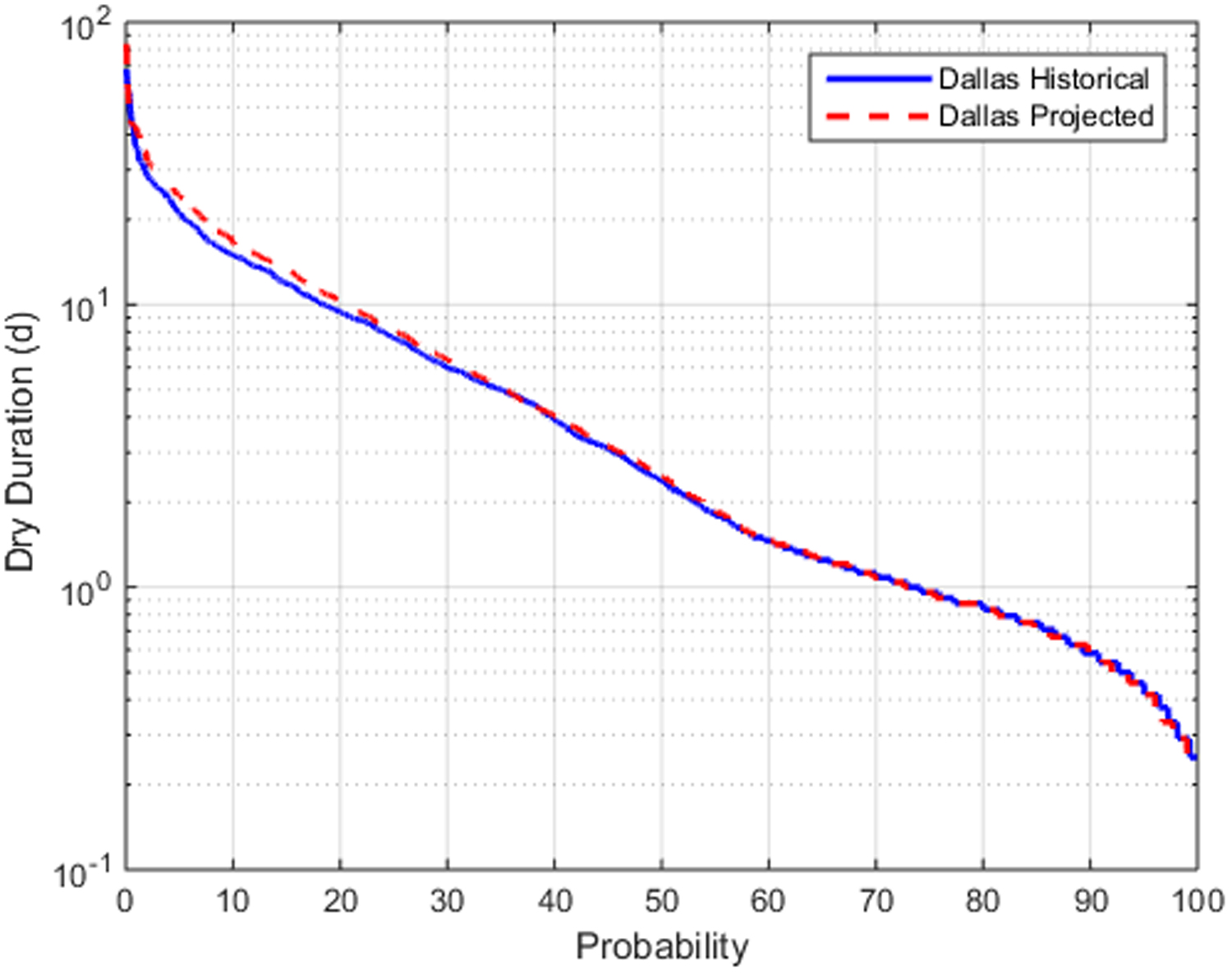

Supplement: Supplementary file 3 — Supplementary material [file mmc3.zip › B3.jpg]

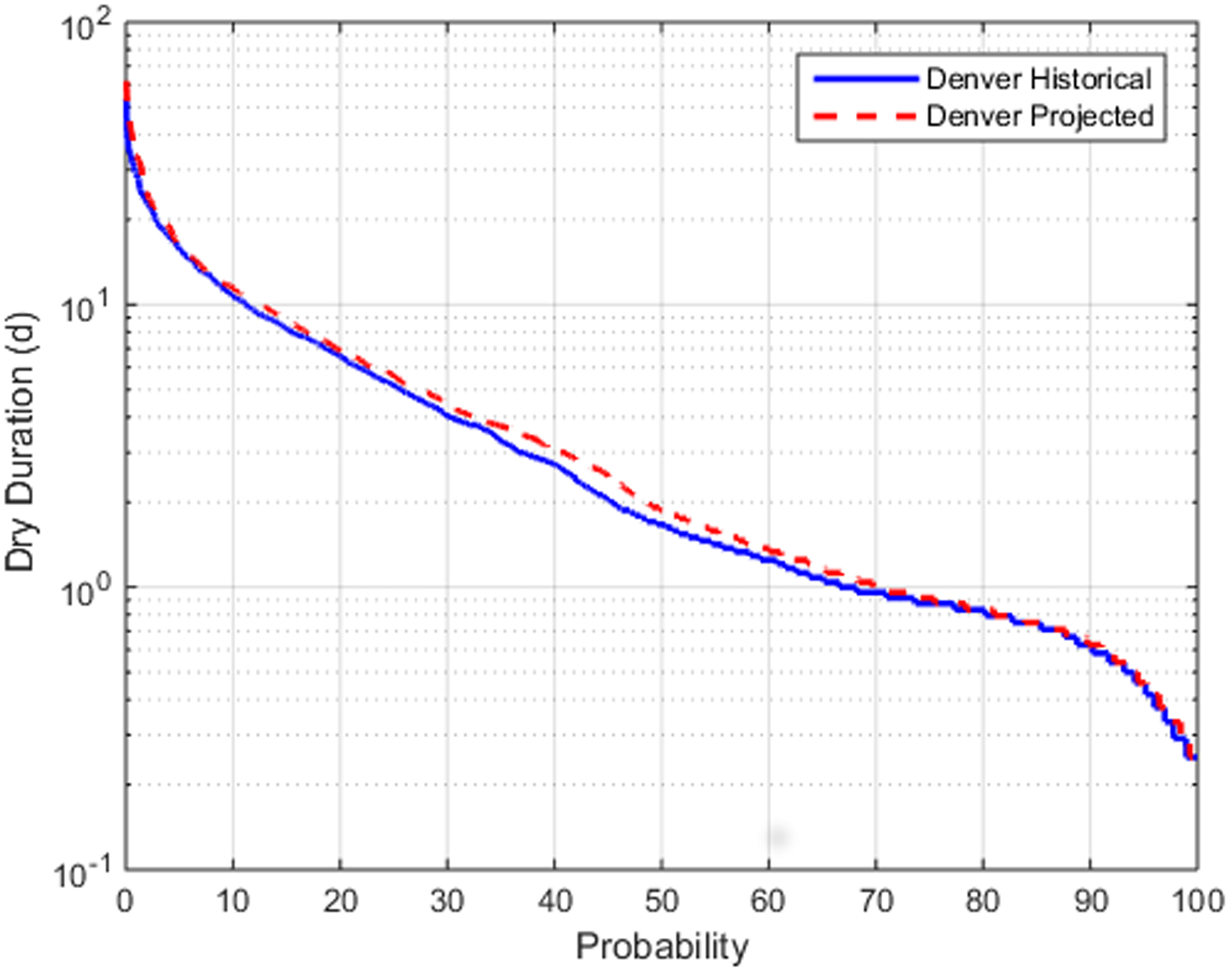

Supplement: Supplementary file 3 — Supplementary material [file mmc3.zip › B4.jpg]

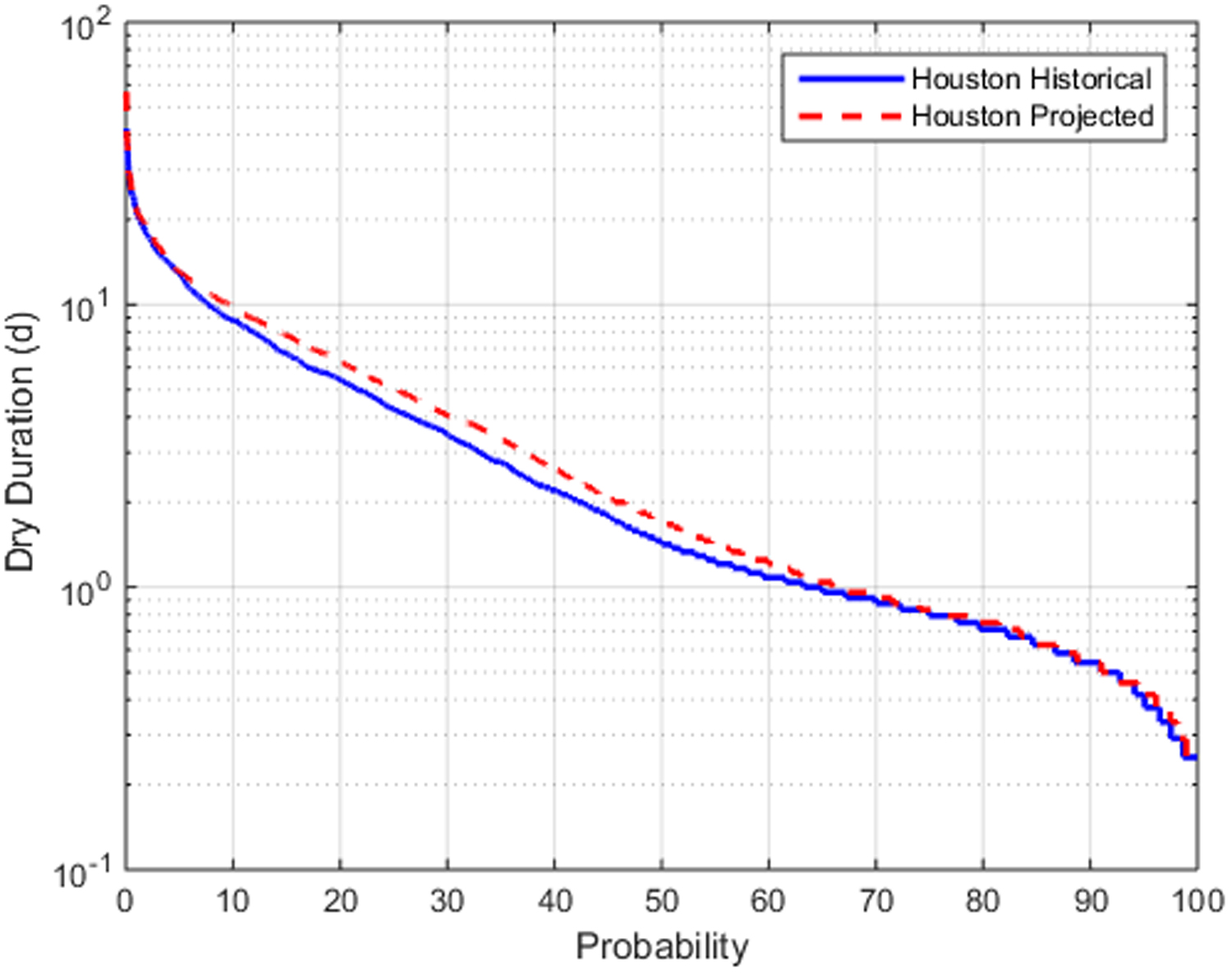

Supplement: Supplementary file 3 — Supplementary material [file mmc3.zip › B5.jpg]

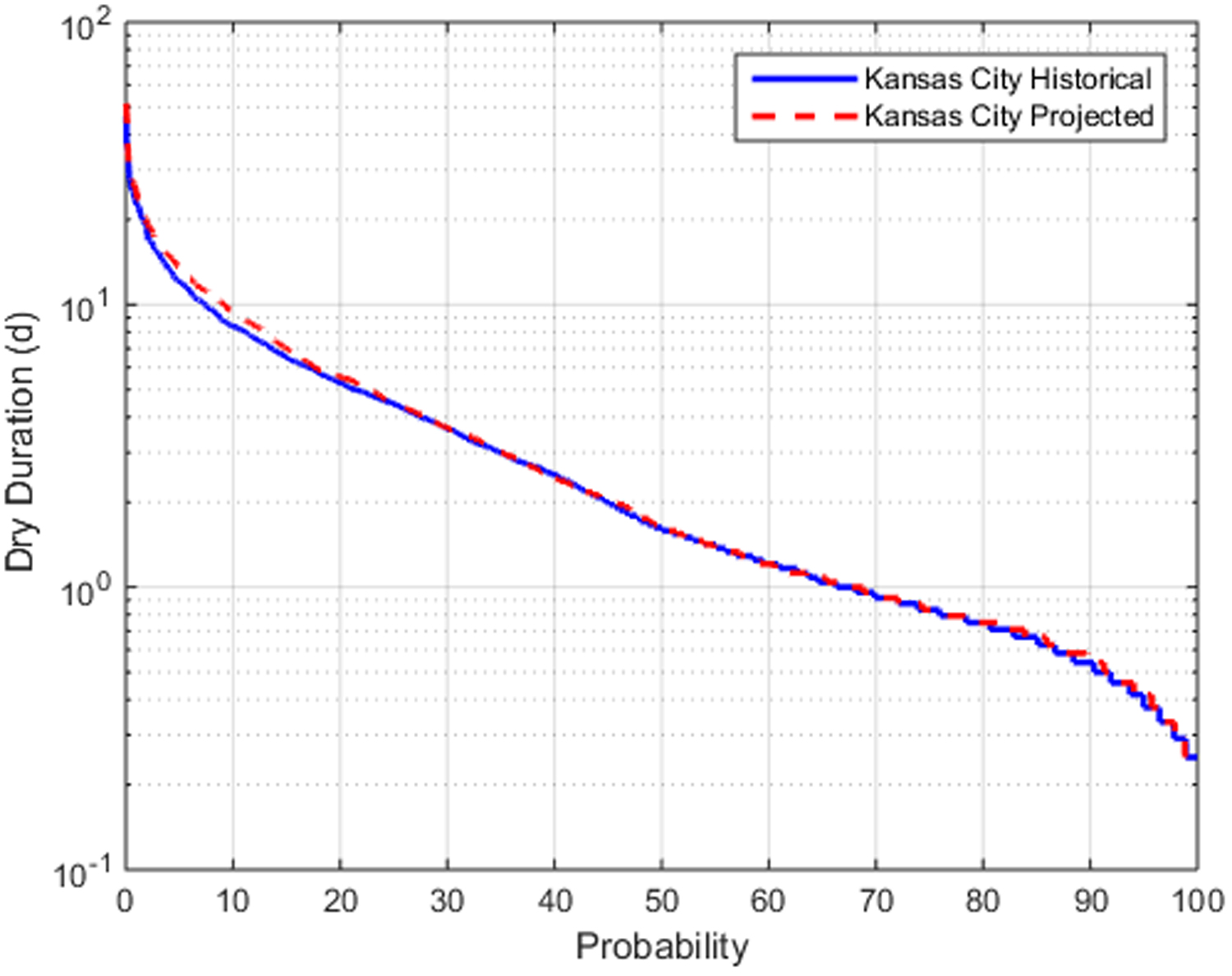

Supplement: Supplementary file 3 — Supplementary material [file mmc3.zip › B6.jpg]

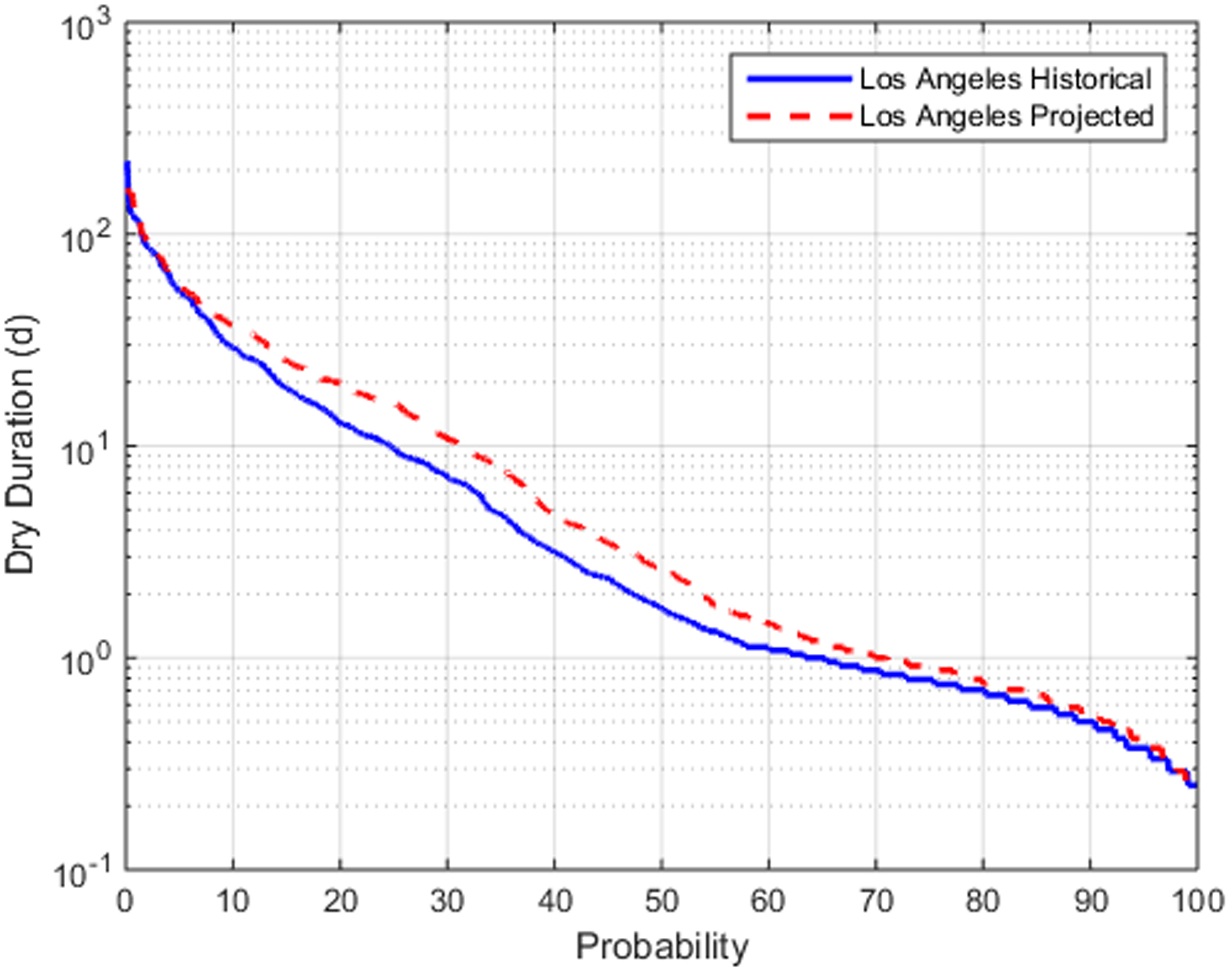

Supplement: Supplementary file 3 — Supplementary material [file mmc3.zip › B7.jpg]

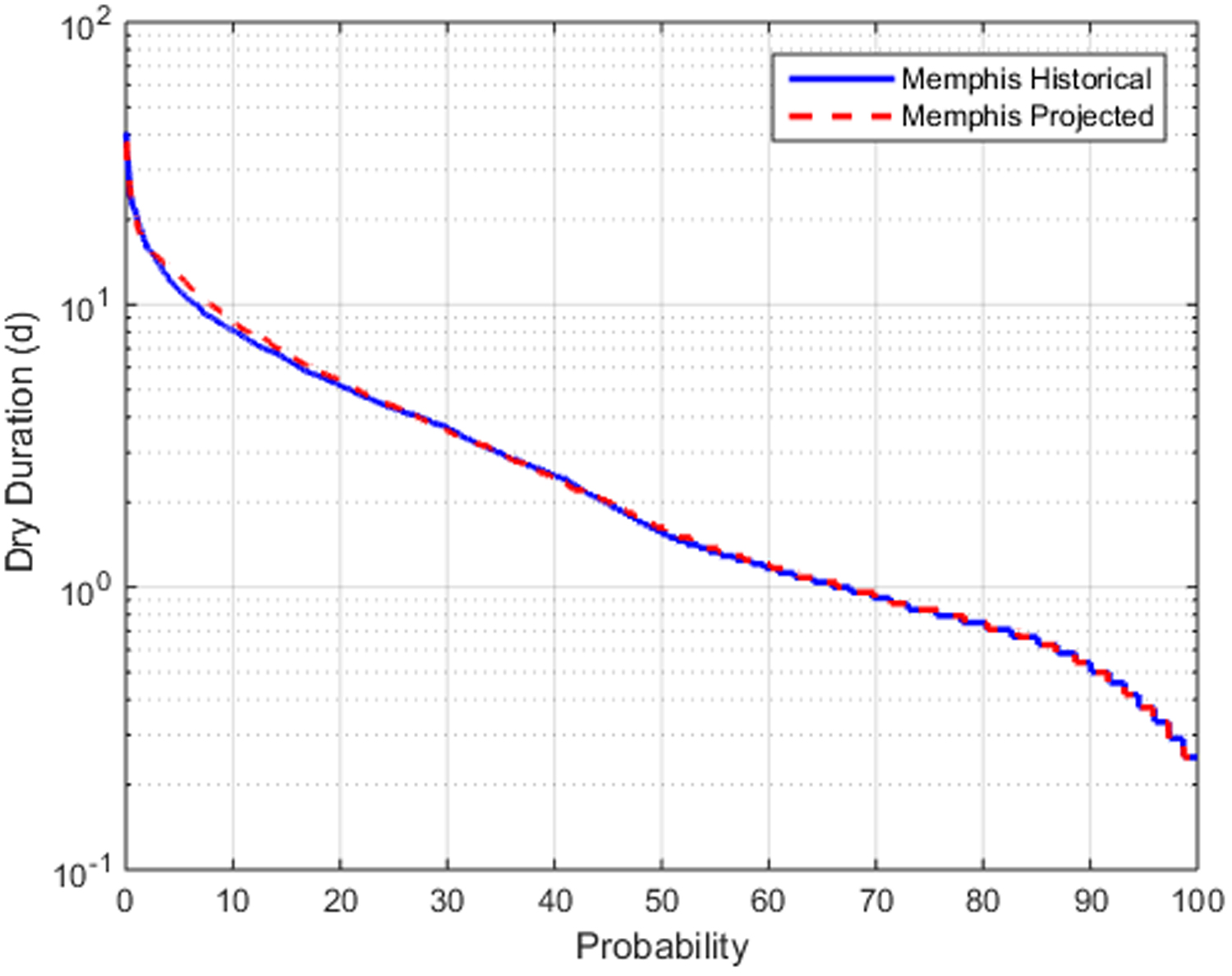

Supplement: Supplementary file 3 — Supplementary material [file mmc3.zip › B8.jpg]

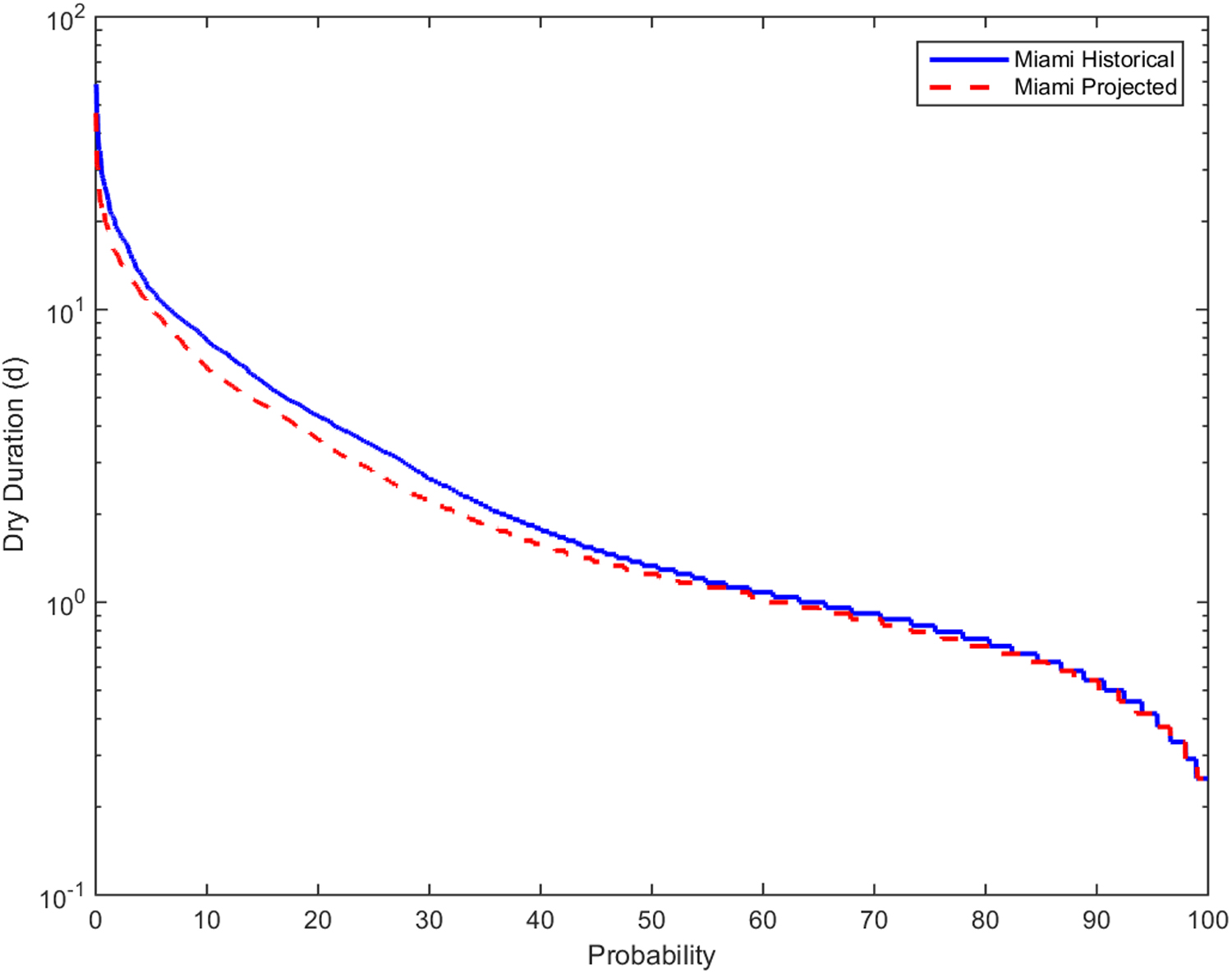

Supplement: Supplementary file 3 — Supplementary material [file mmc3.zip › B9.jpg]

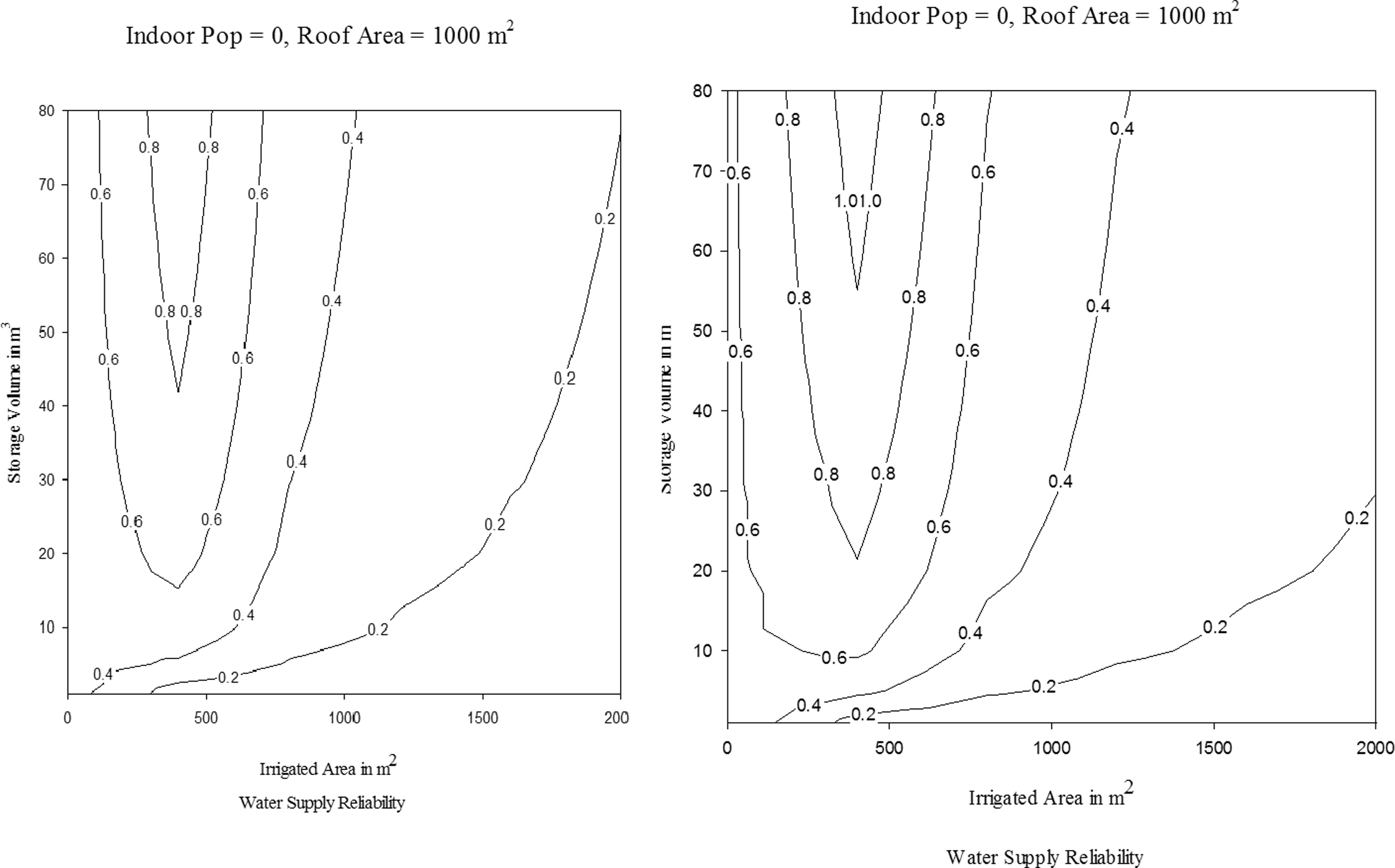

Supplement: Supplementary file 4 — Supplementary material [file mmc4.zip › C1.jpg]

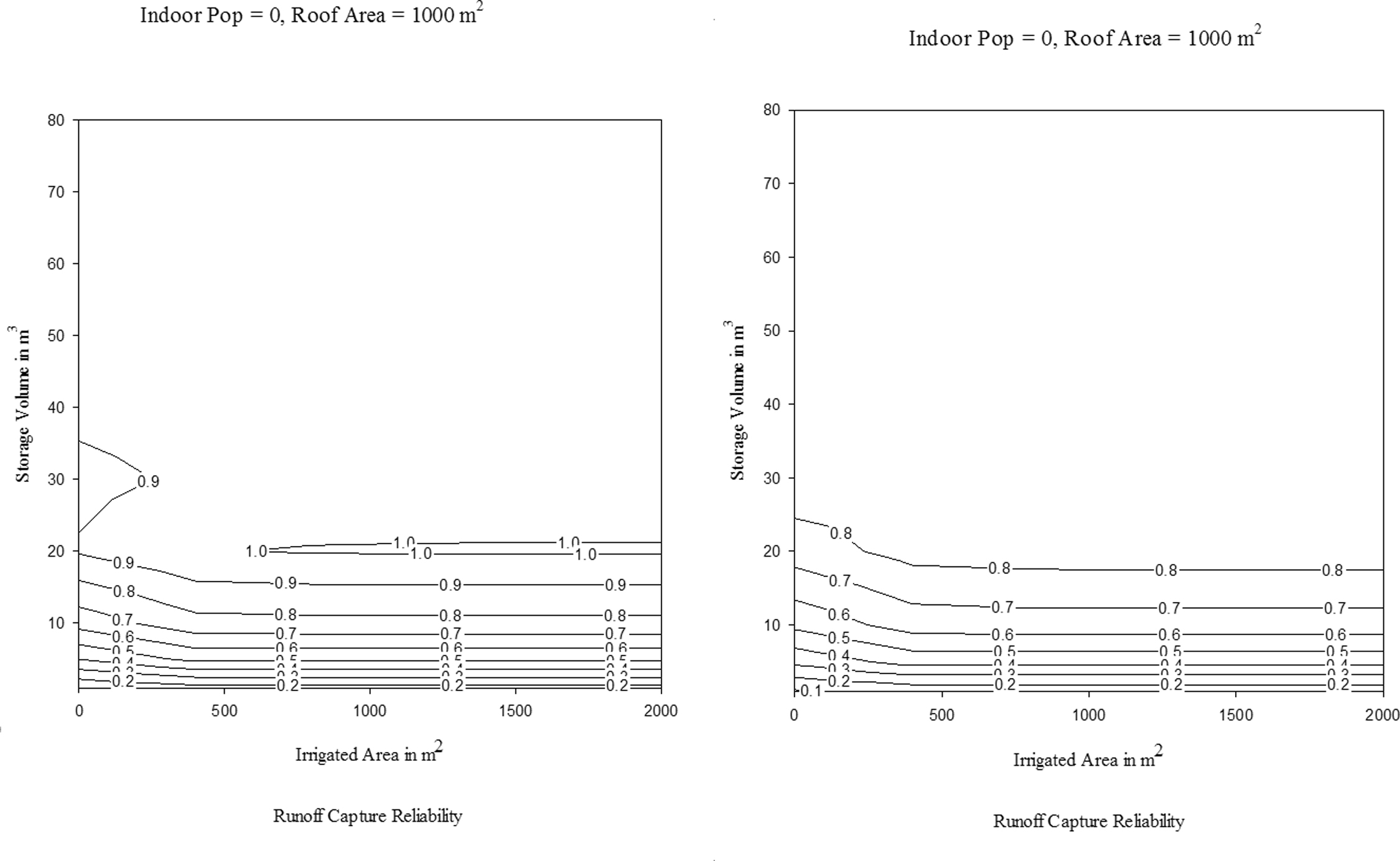

Supplement: Supplementary file 4 — Supplementary material [file mmc4.zip › C10.jpg]

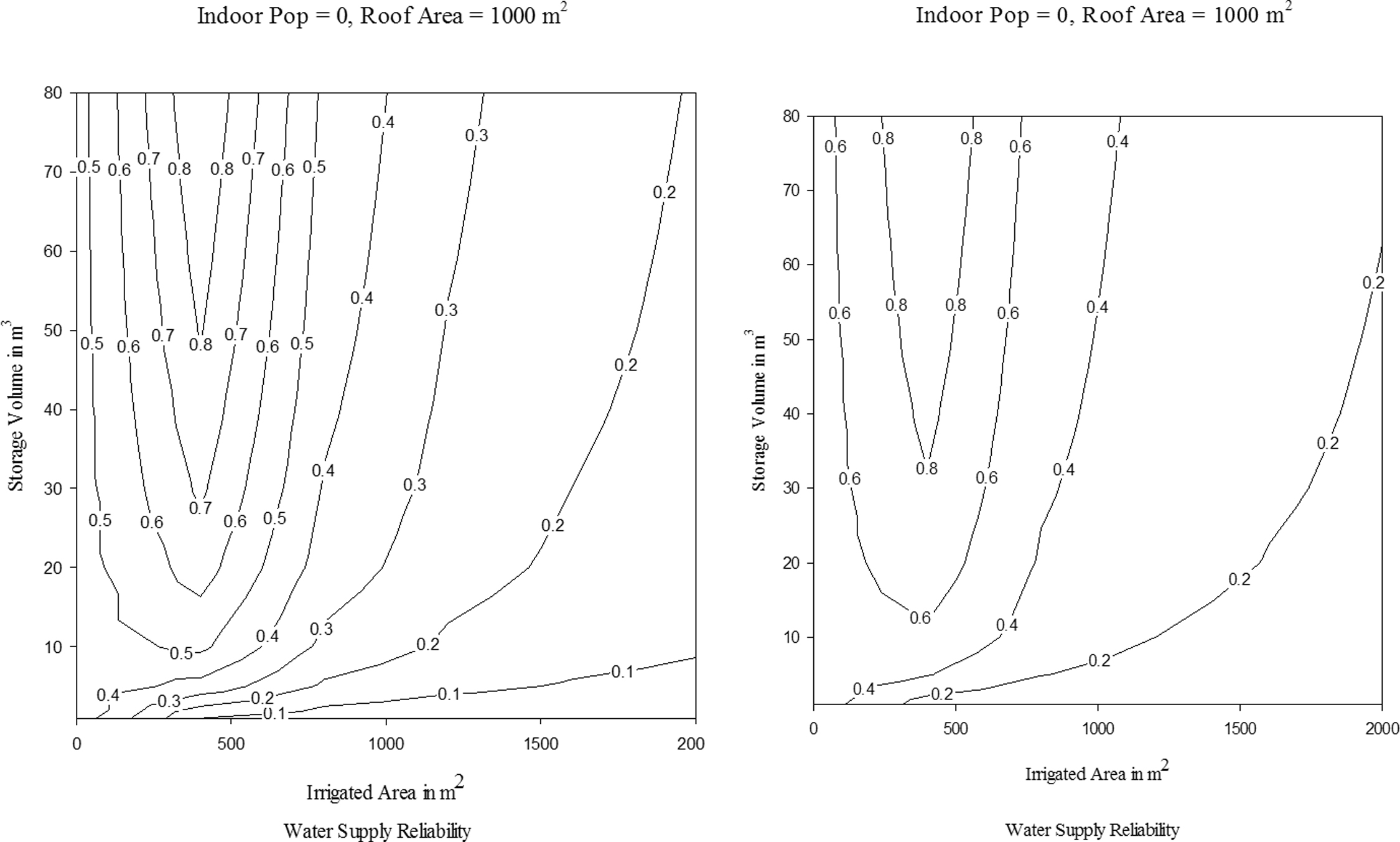

Supplement: Supplementary file 4 — Supplementary material [file mmc4.zip › C11.jpg]

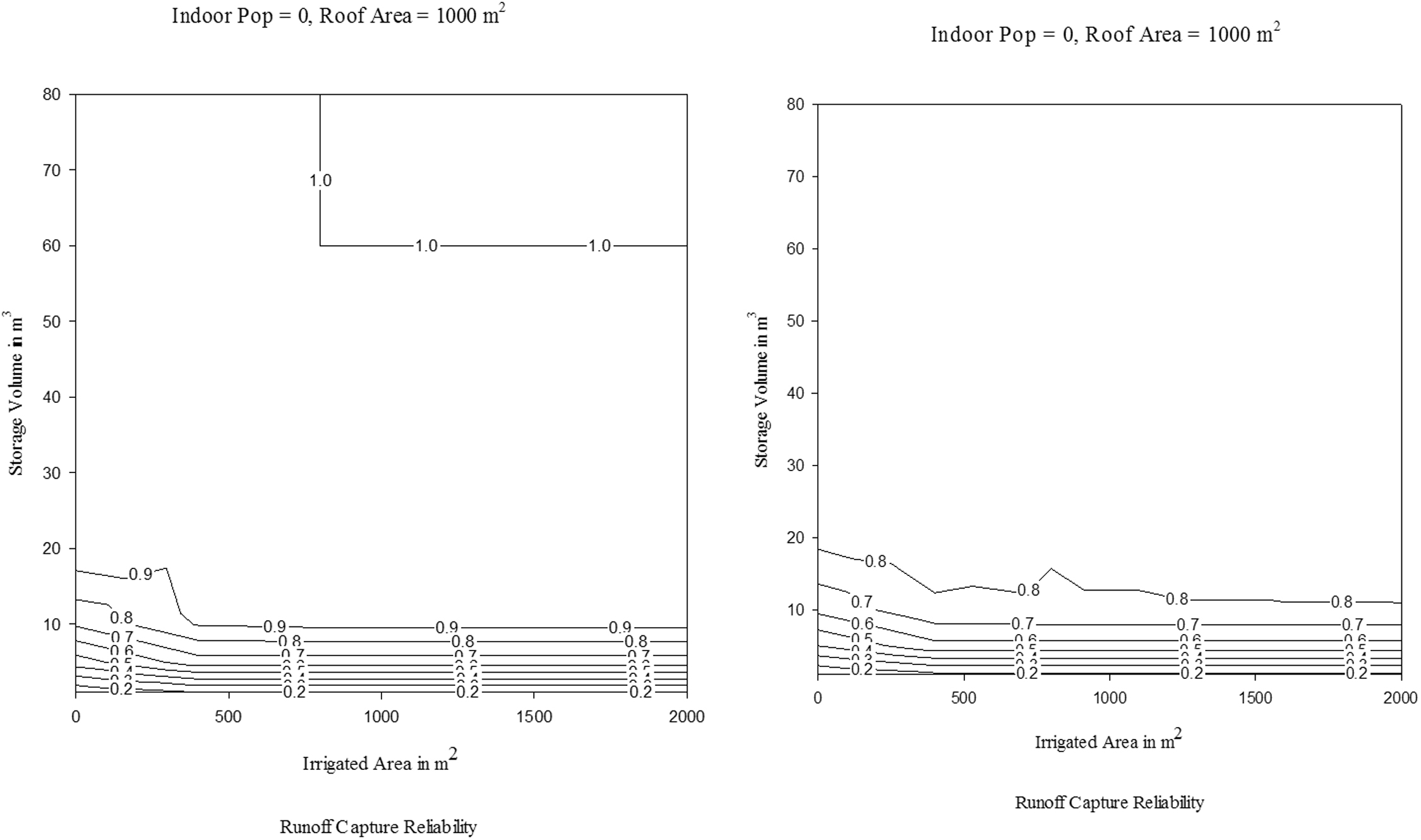

Supplement: Supplementary file 4 — Supplementary material [file mmc4.zip › C12.jpg]

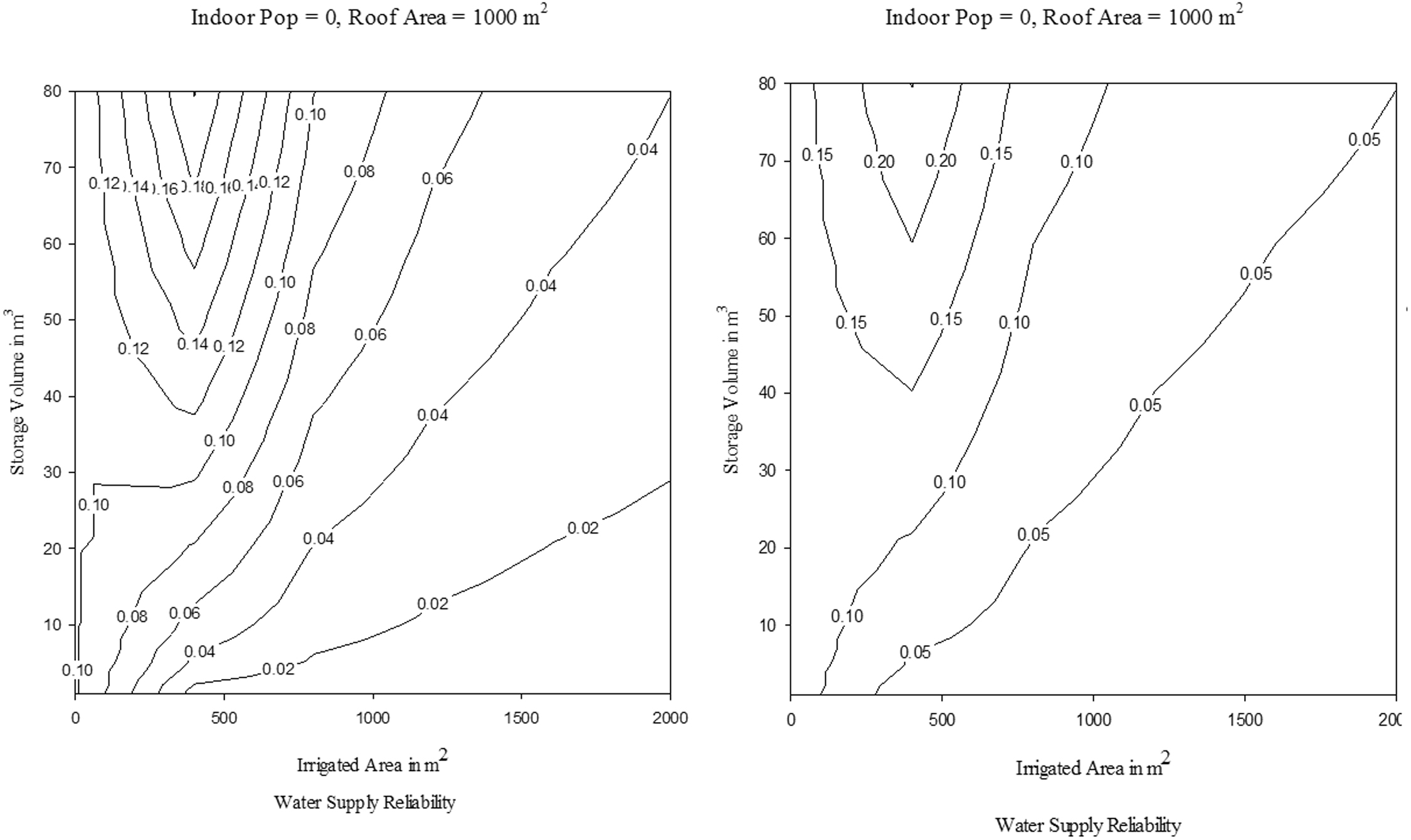

Supplement: Supplementary file 4 — Supplementary material [file mmc4.zip › C13.jpg]

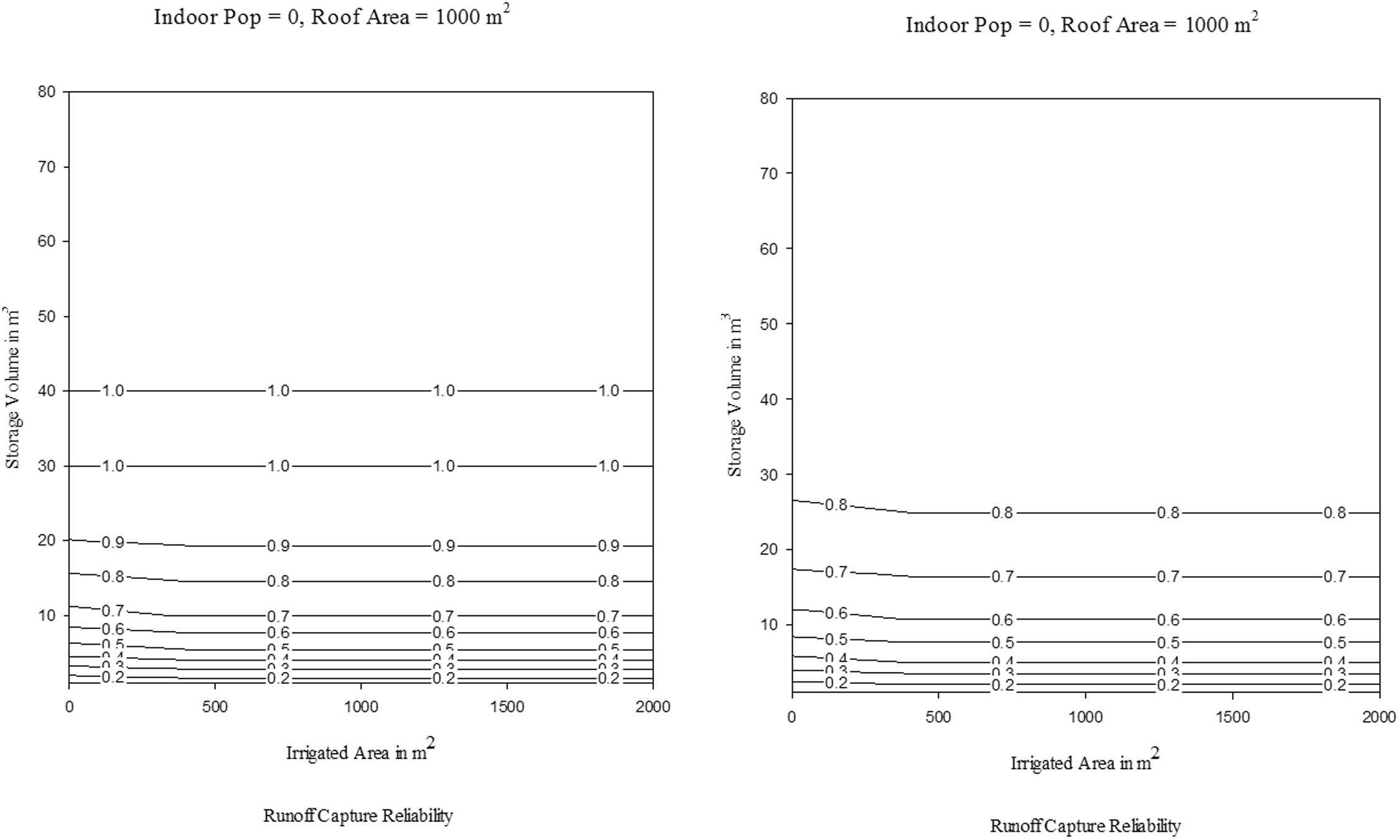

Supplement: Supplementary file 4 — Supplementary material [file mmc4.zip › C14.jpg]

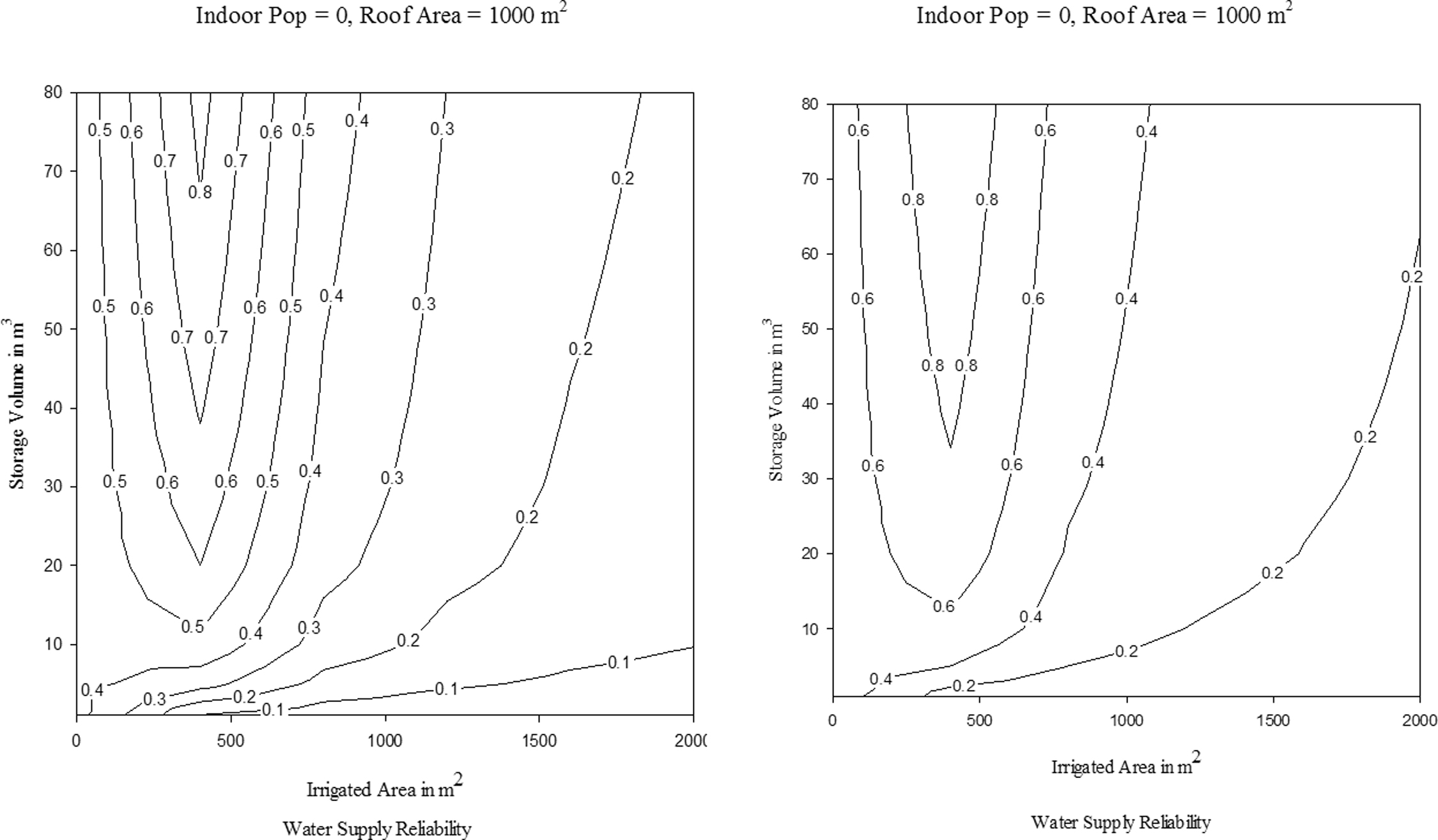

Supplement: Supplementary file 4 — Supplementary material [file mmc4.zip › C15.jpg]

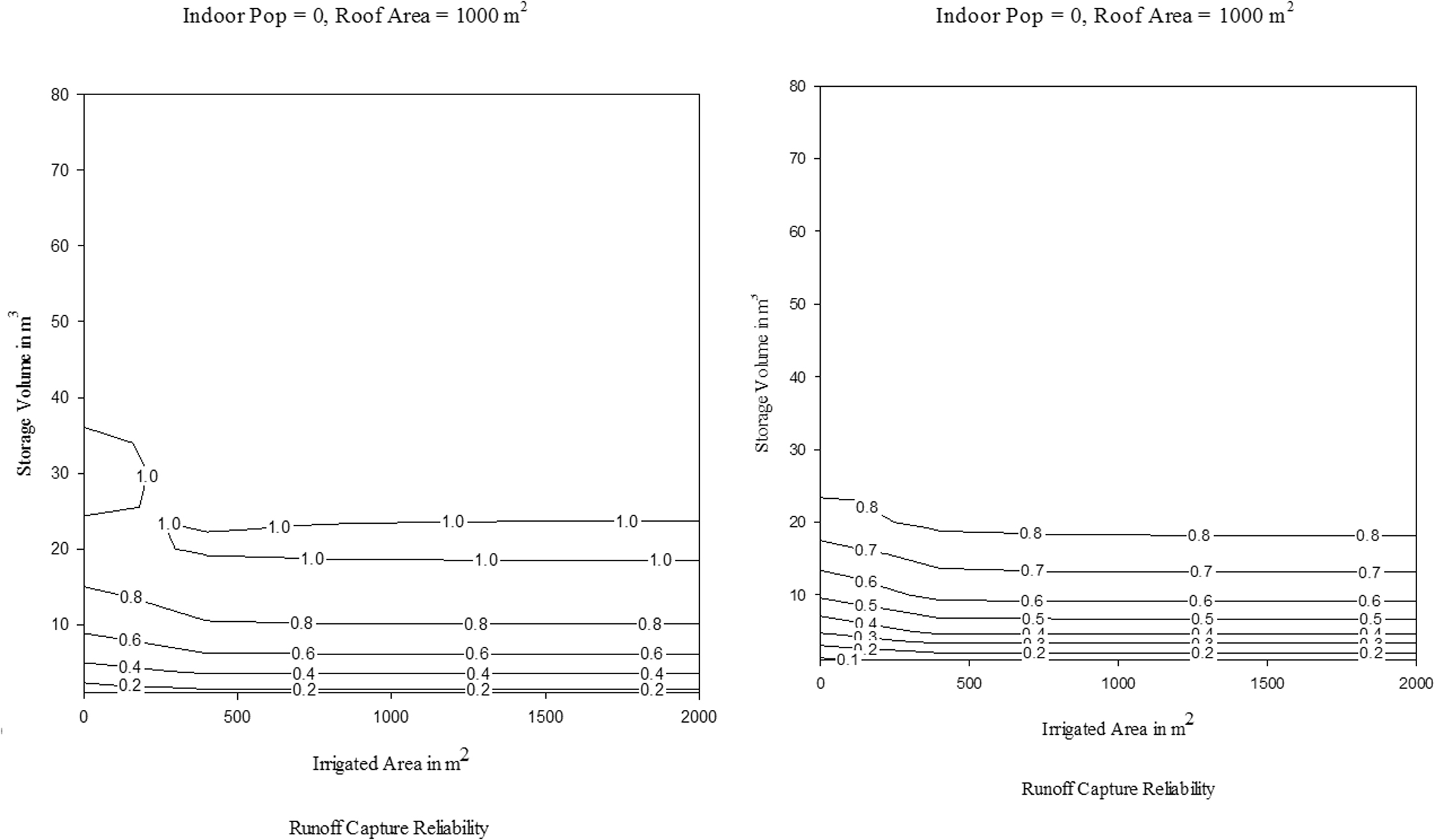

Supplement: Supplementary file 4 — Supplementary material [file mmc4.zip › C16.jpg]

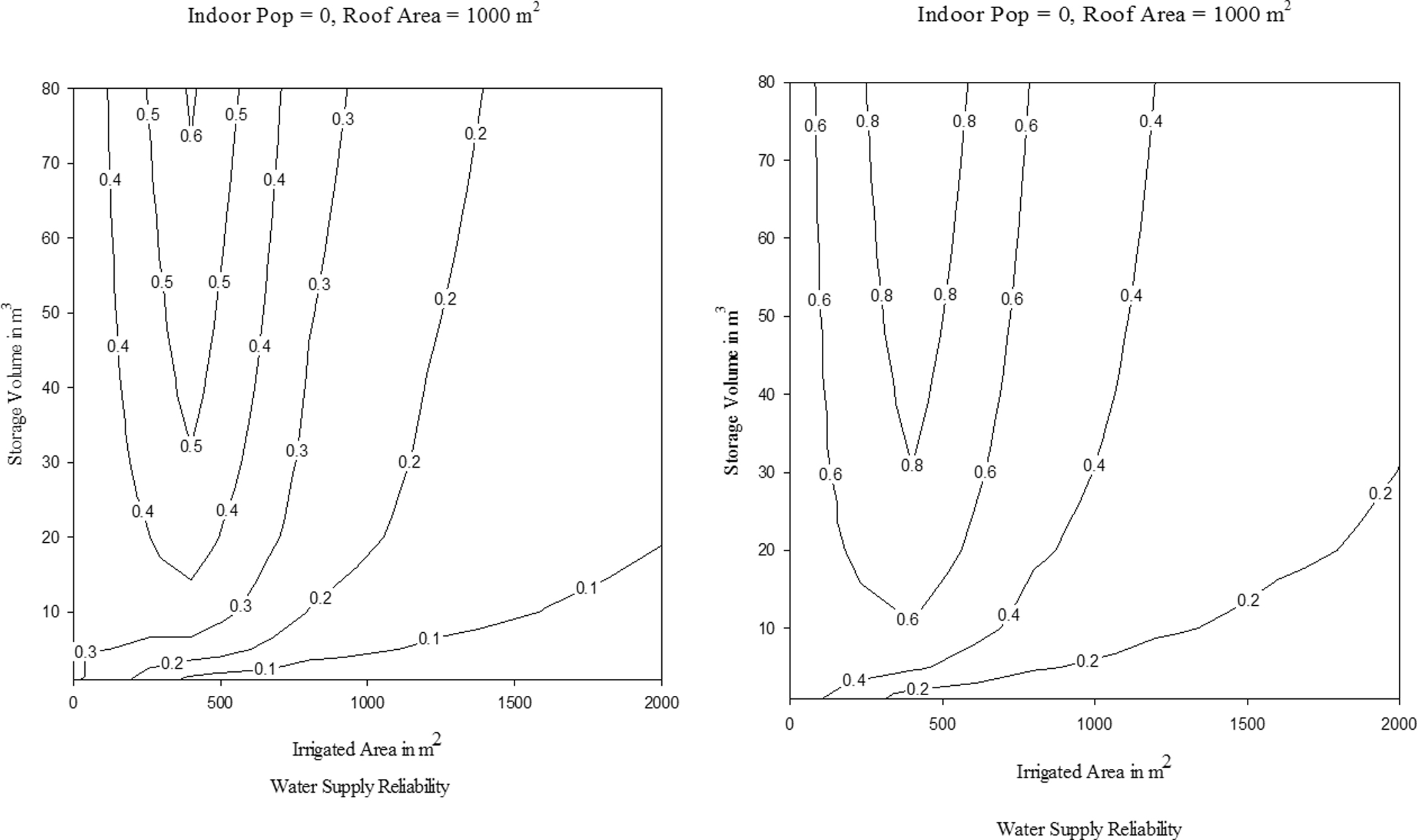

Supplement: Supplementary file 4 — Supplementary material [file mmc4.zip › C17.jpg]

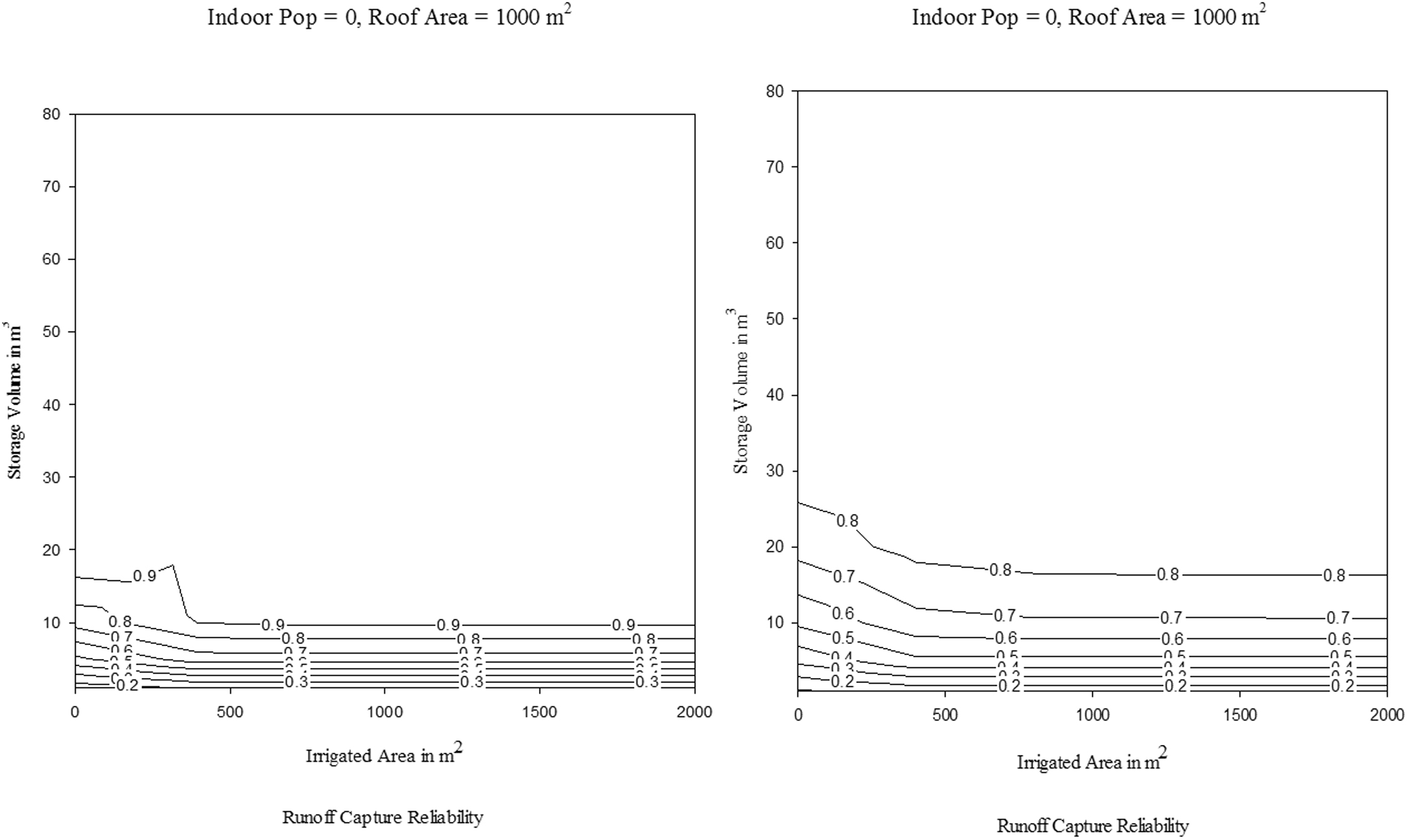

Supplement: Supplementary file 4 — Supplementary material [file mmc4.zip › C18.jpg]

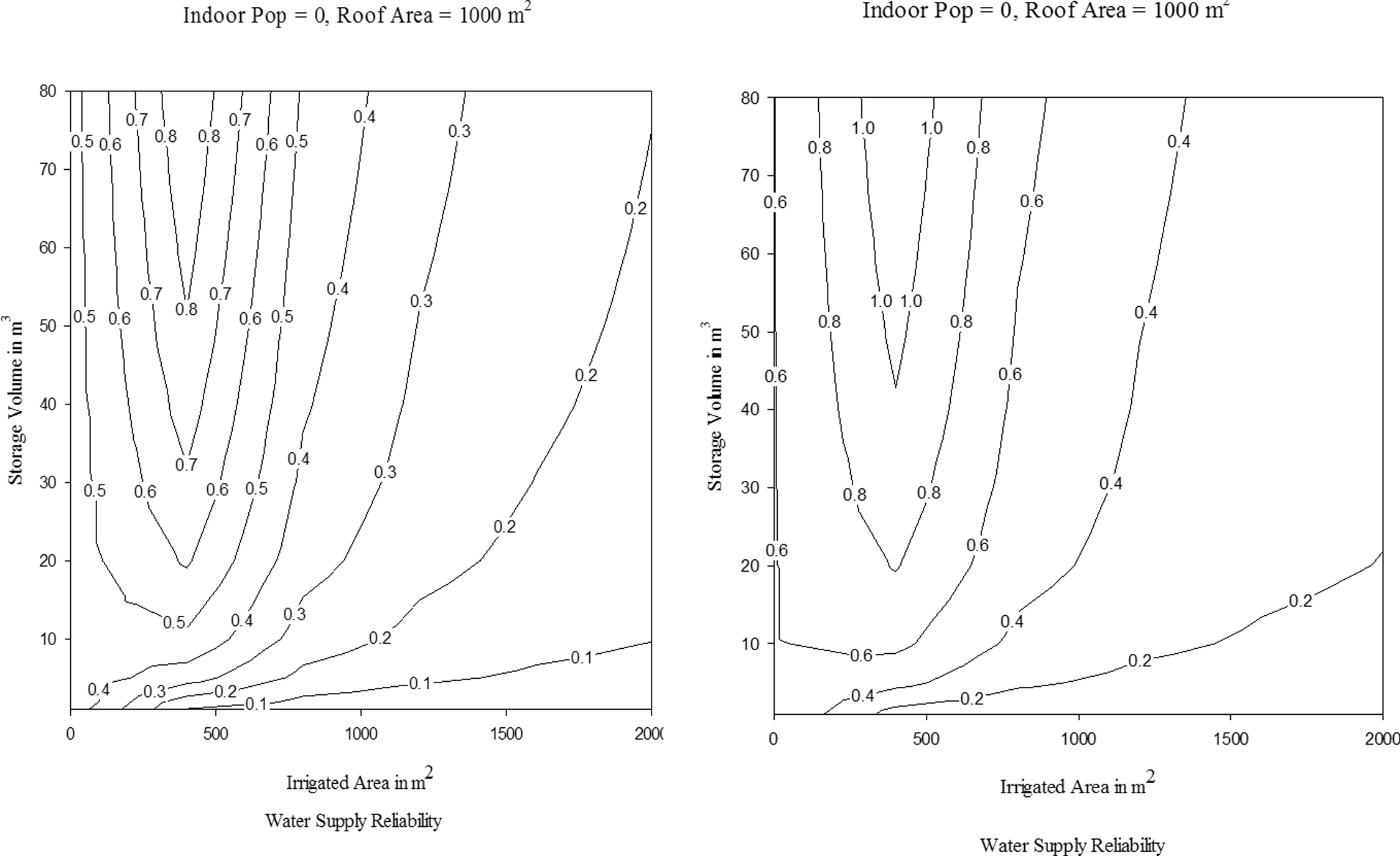

Supplement: Supplementary file 4 — Supplementary material [file mmc4.zip › C19.jpg]

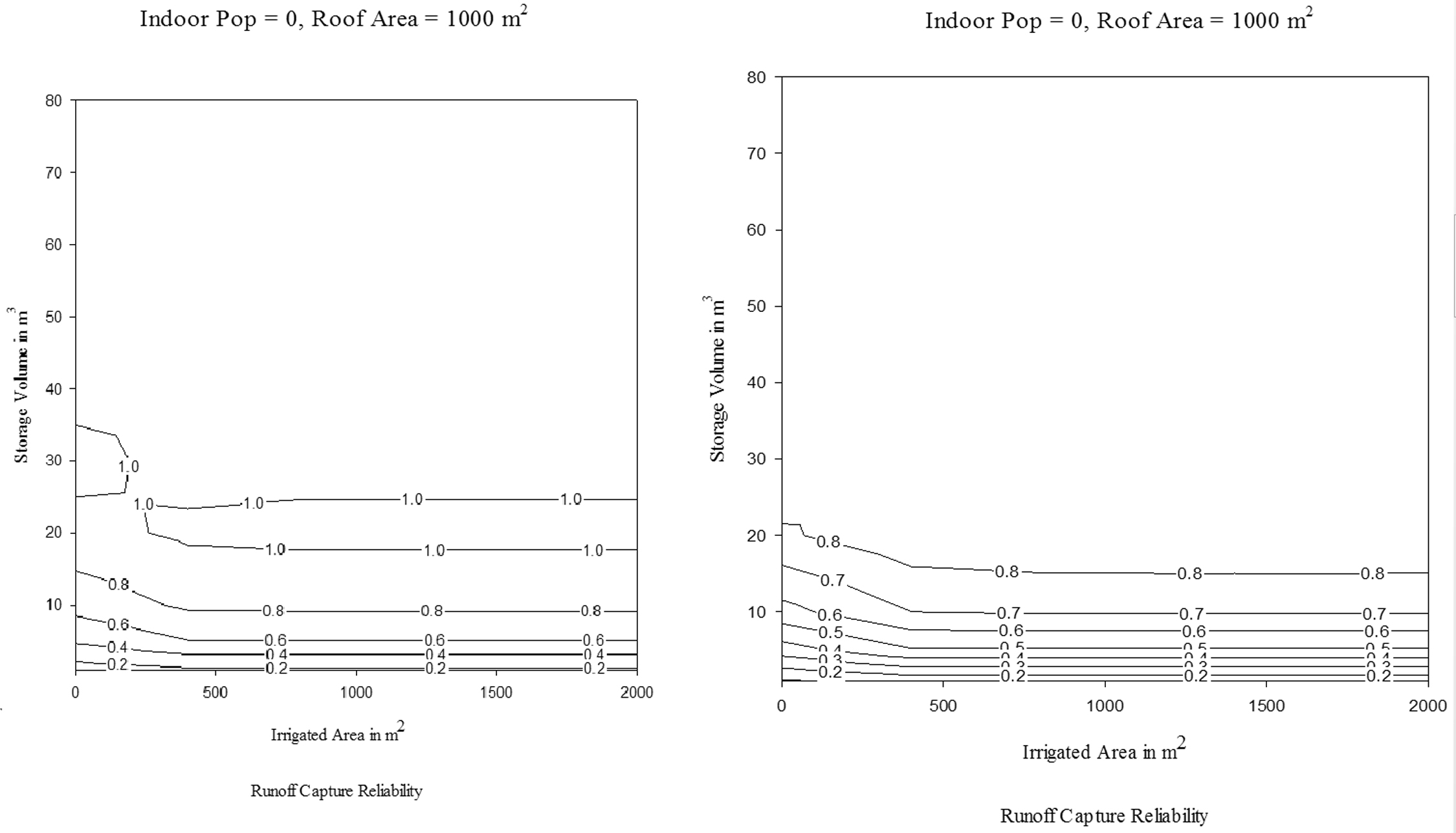

Supplement: Supplementary file 4 — Supplementary material [file mmc4.zip › C2.jpg]

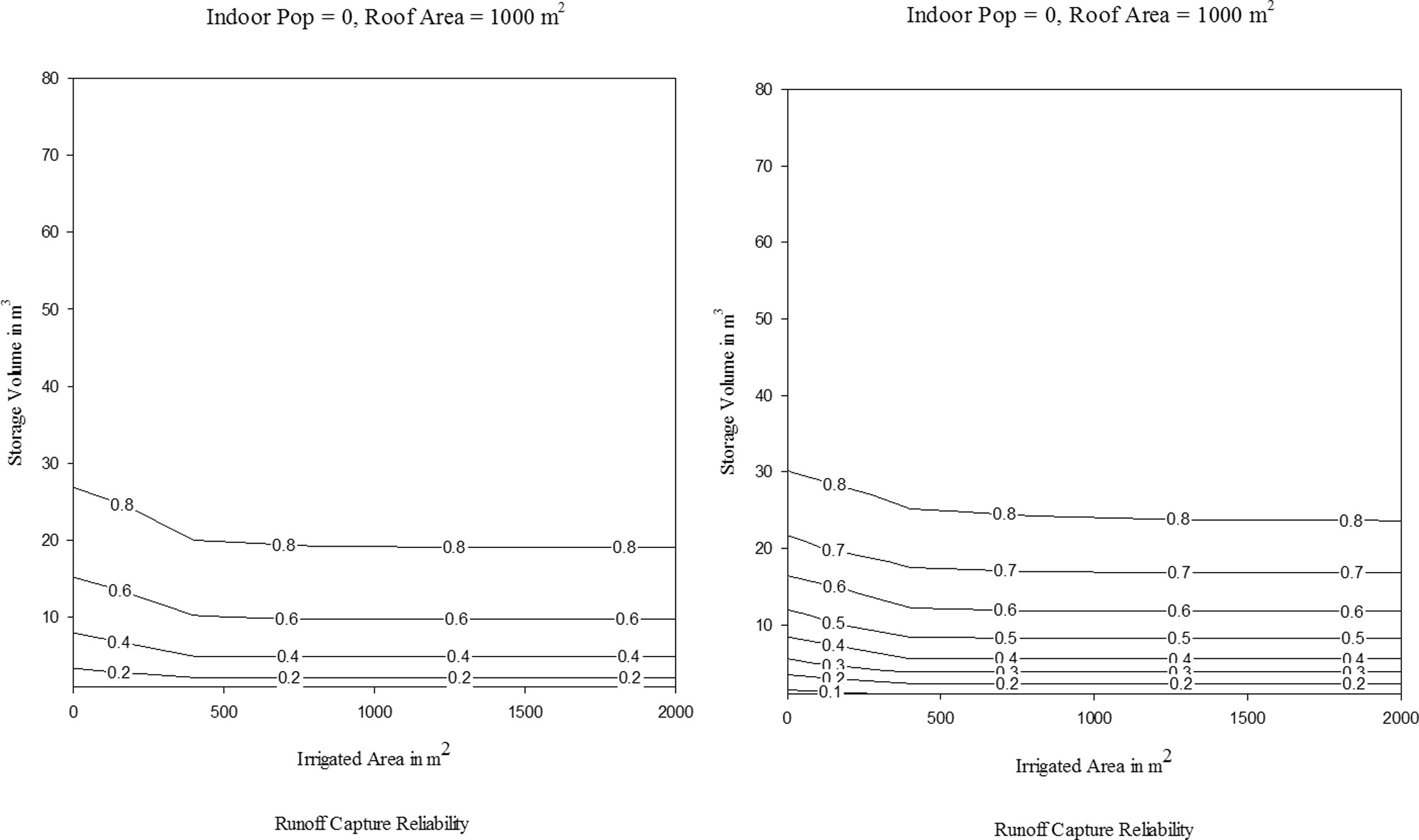

Supplement: Supplementary file 4 — Supplementary material [file mmc4.zip › C20.jpg]

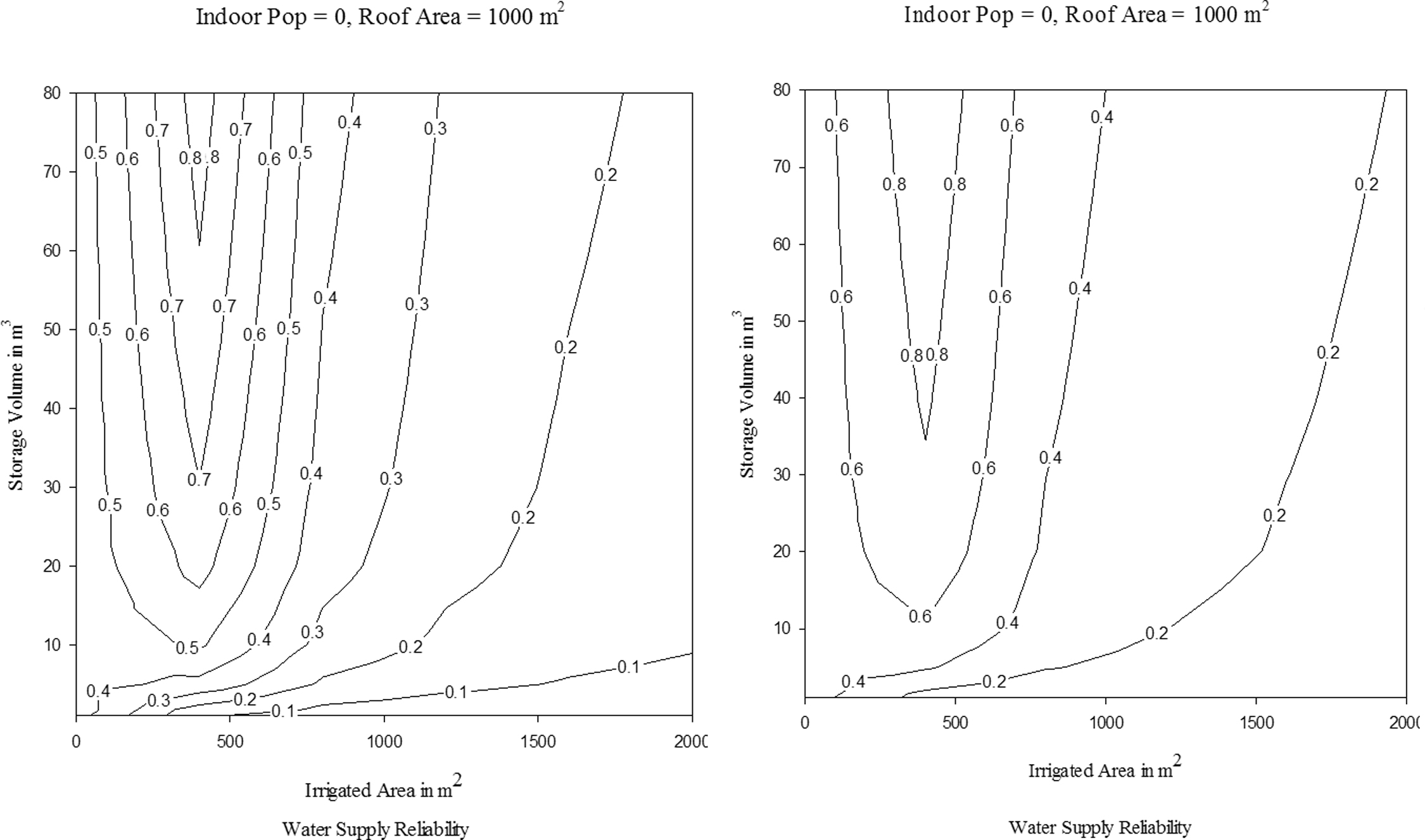

Supplement: Supplementary file 4 — Supplementary material [file mmc4.zip › C21.jpg]

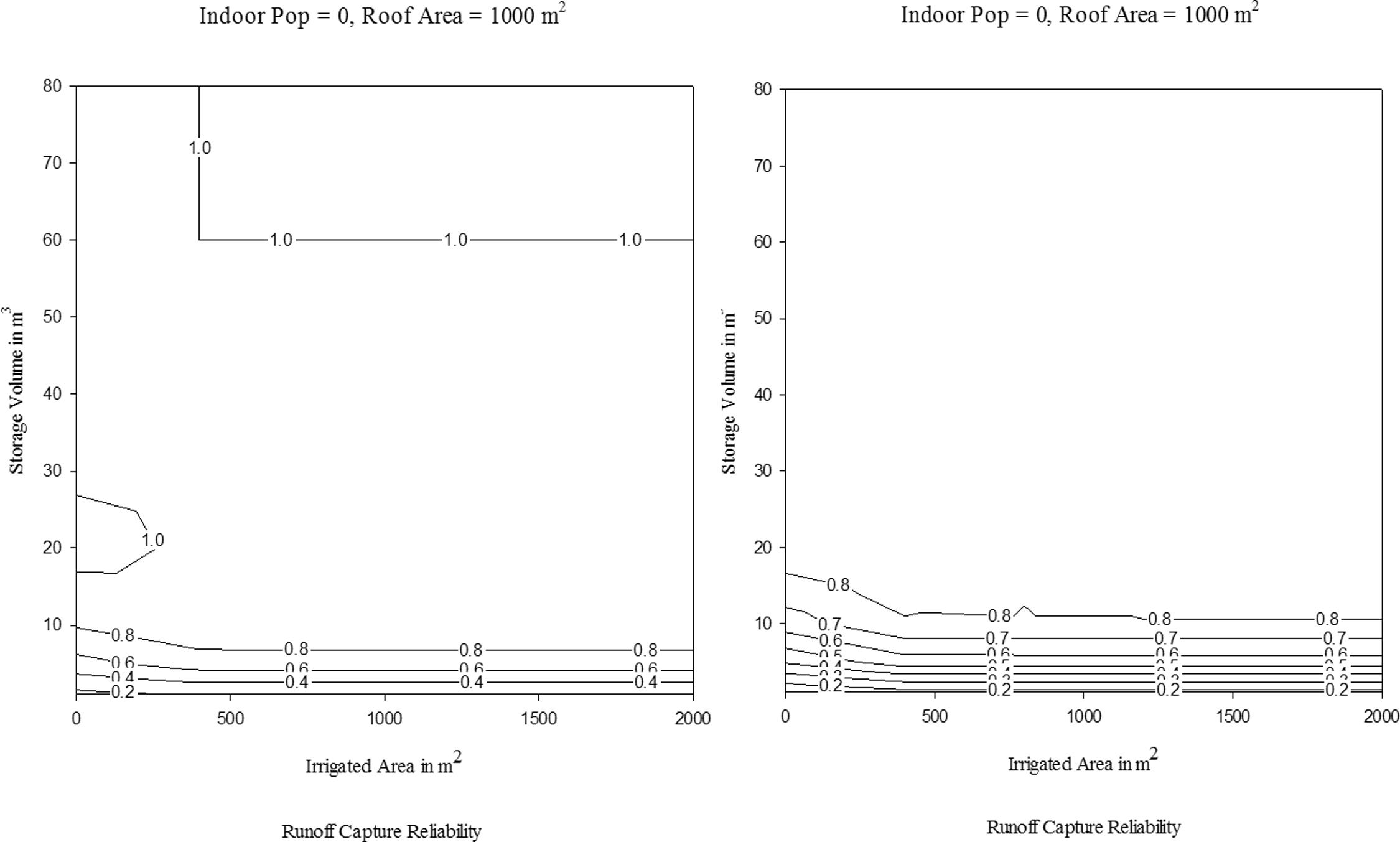

Supplement: Supplementary file 4 — Supplementary material [file mmc4.zip › C22.jpg]

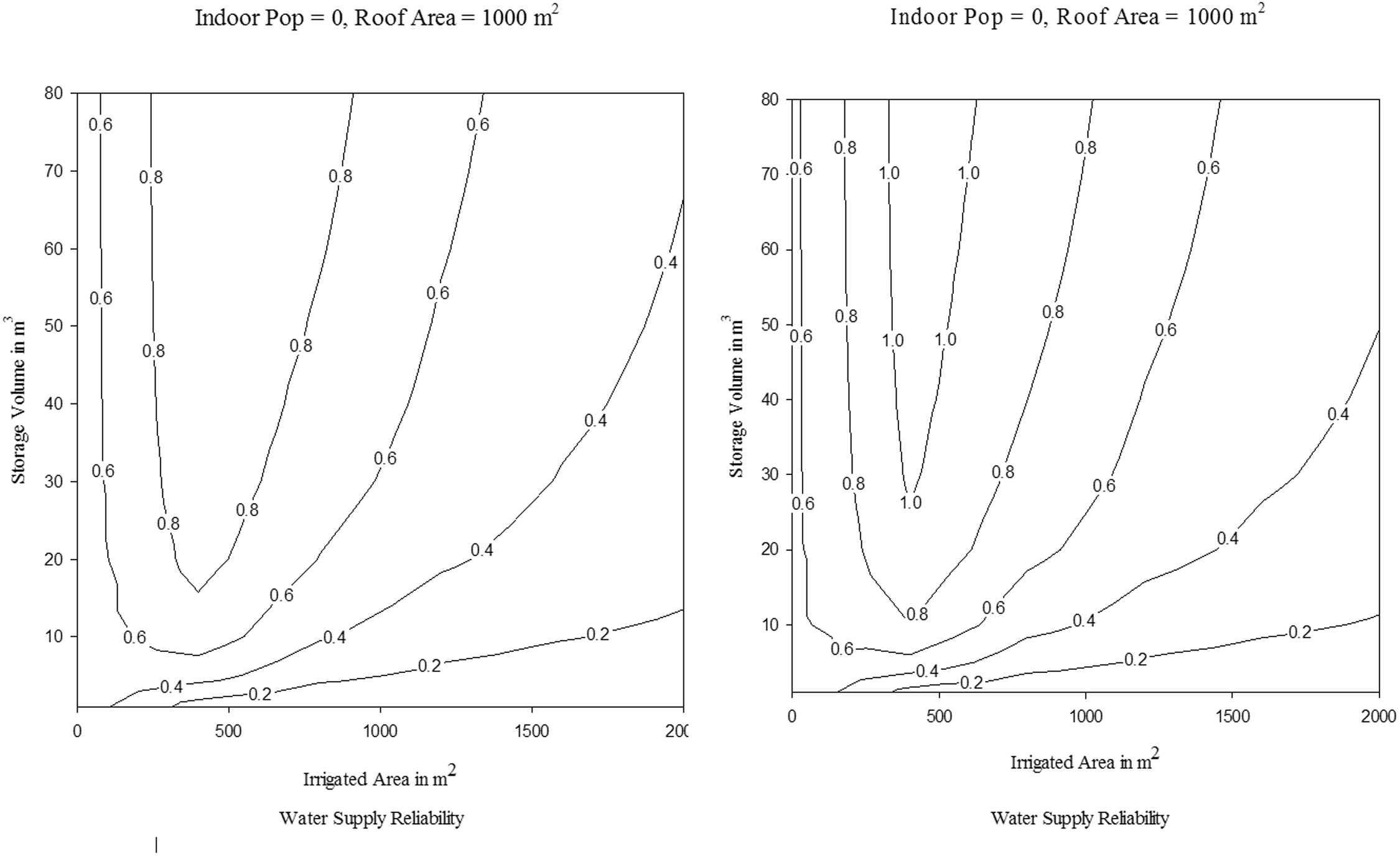

Supplement: Supplementary file 4 — Supplementary material [file mmc4.zip › C23.jpg]

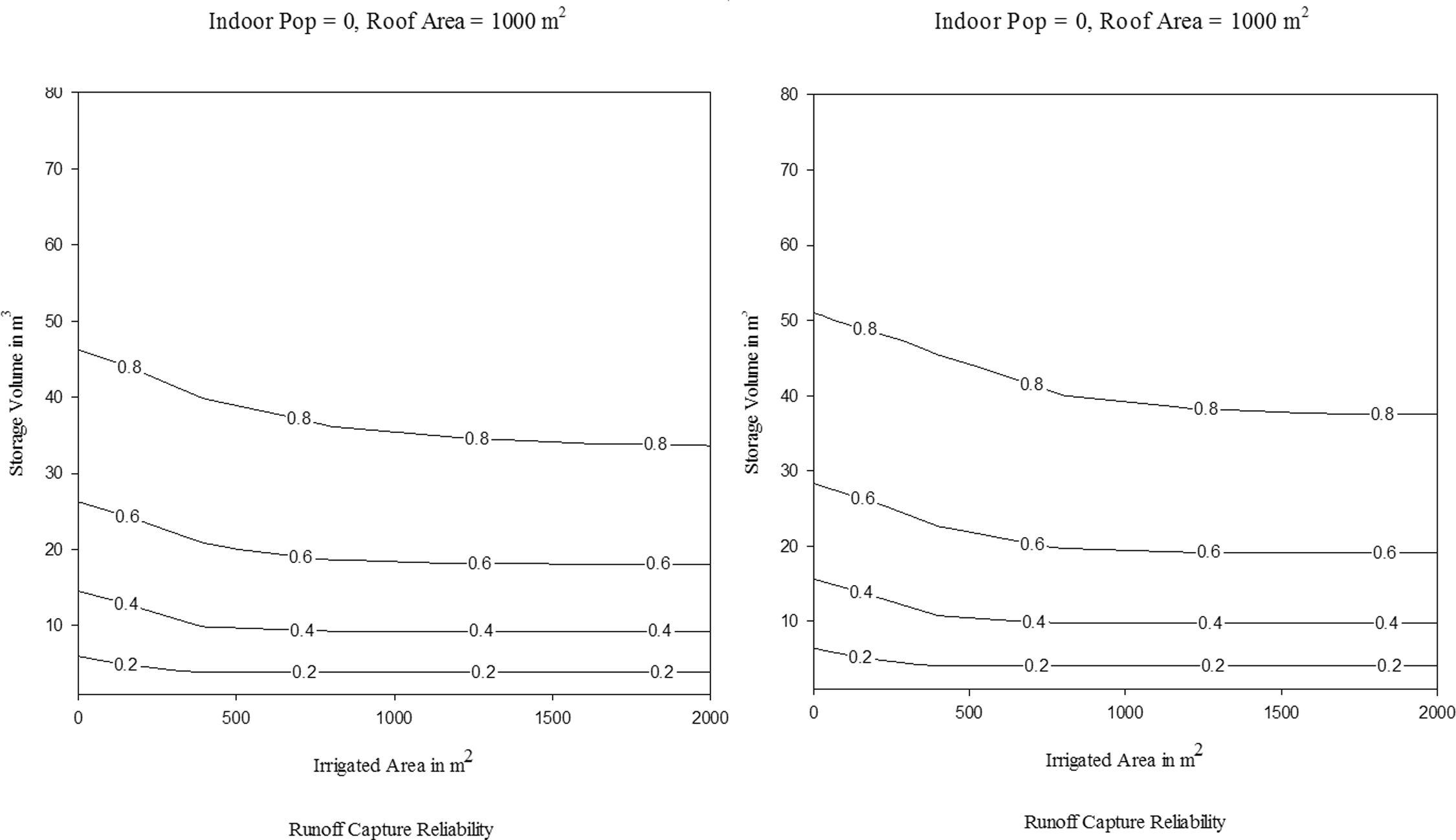

Supplement: Supplementary file 4 — Supplementary material [file mmc4.zip › C24.jpg]

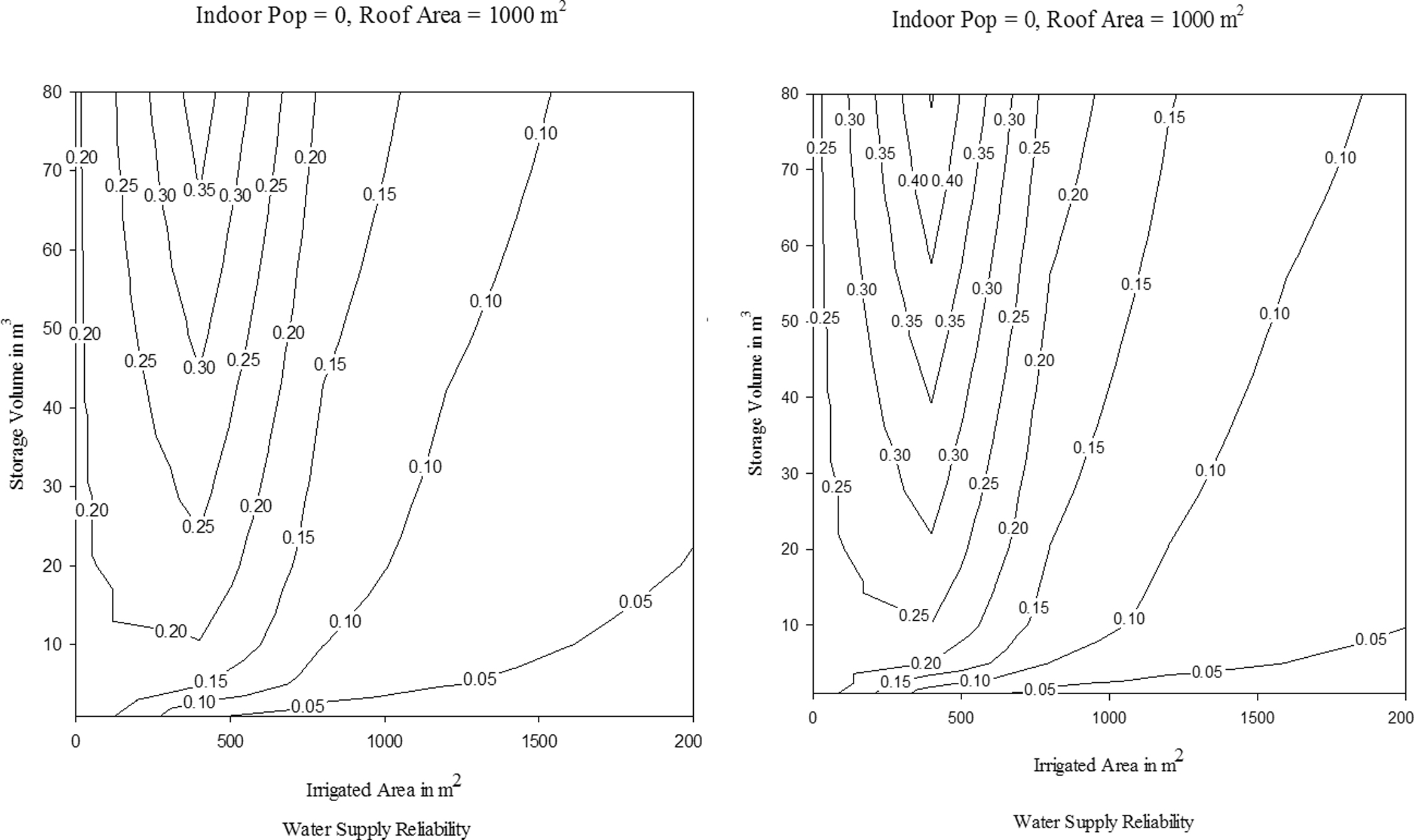

Supplement: Supplementary file 4 — Supplementary material [file mmc4.zip › C25.jpg]

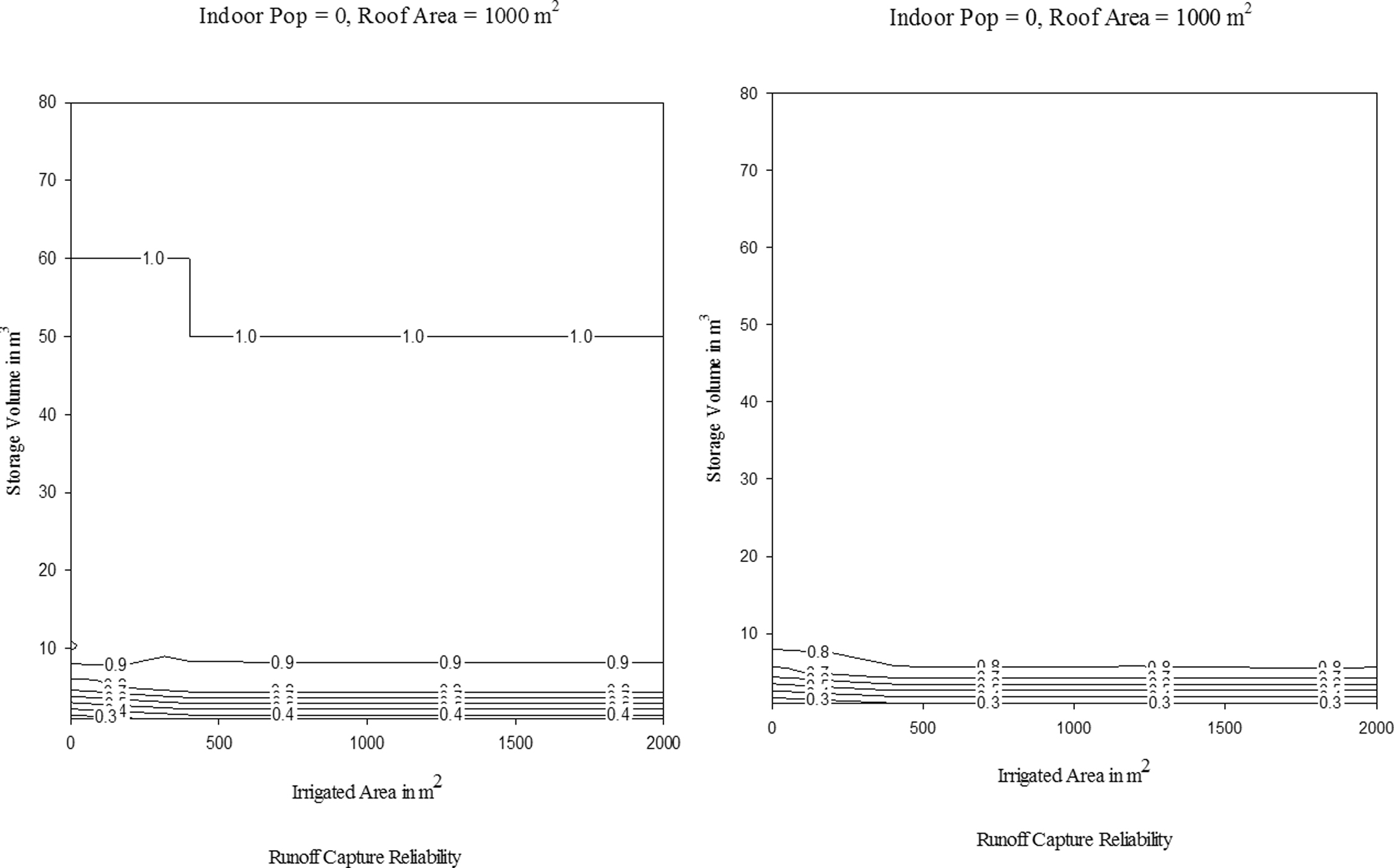

Supplement: Supplementary file 4 — Supplementary material [file mmc4.zip › C26.jpg]

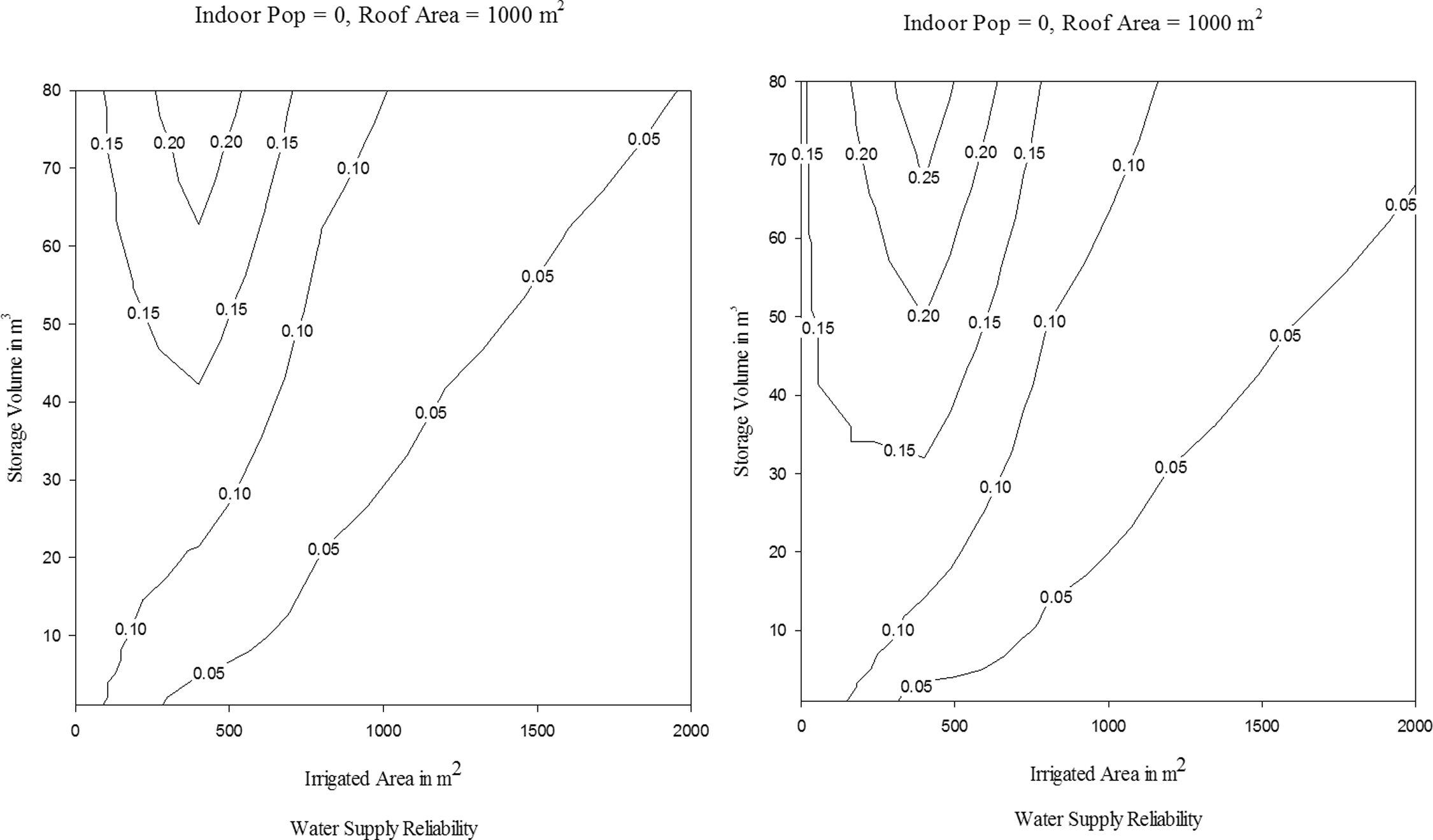

Supplement: Supplementary file 4 — Supplementary material [file mmc4.zip › C27.jpg]

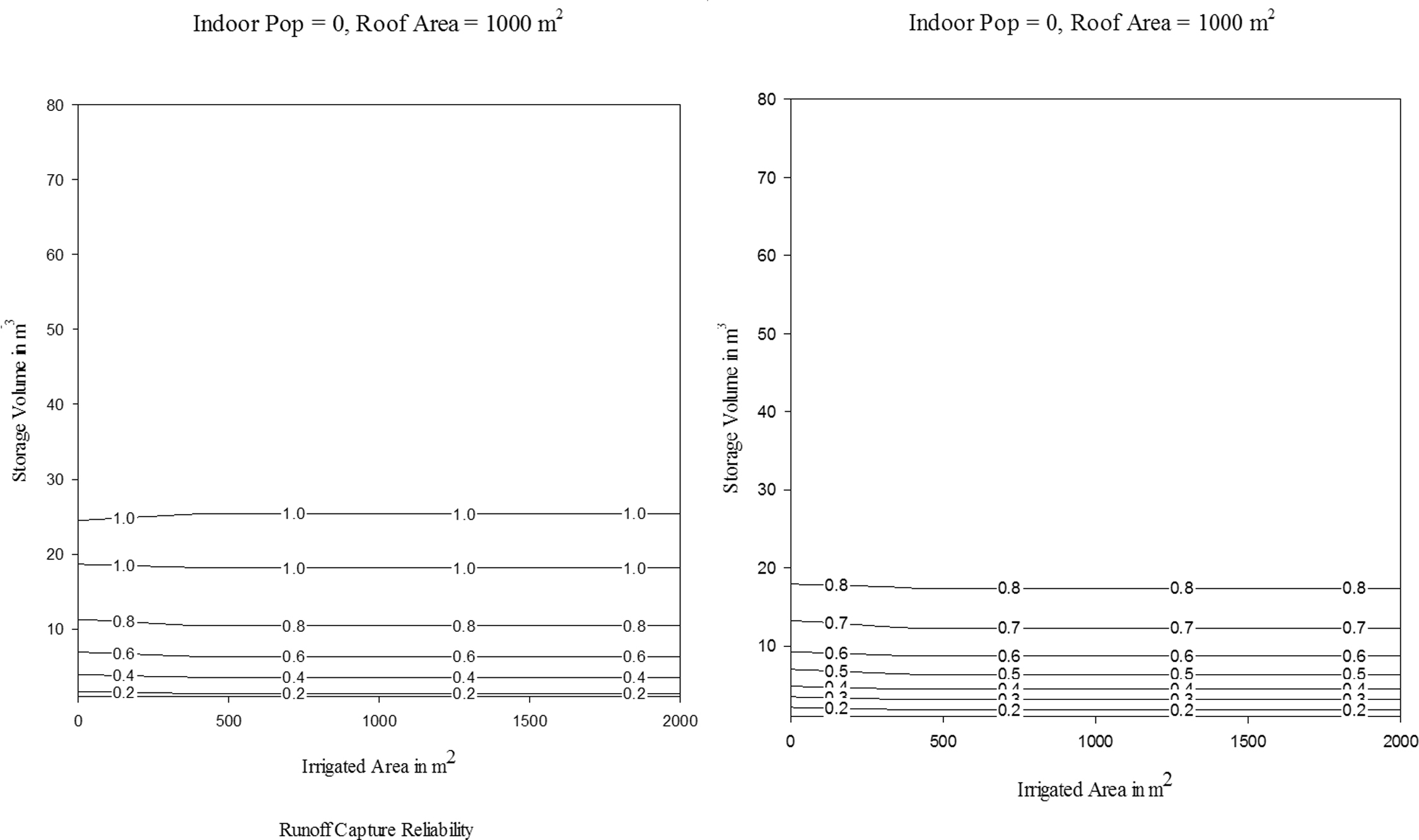

Supplement: Supplementary file 4 — Supplementary material [file mmc4.zip › C28.jpg]

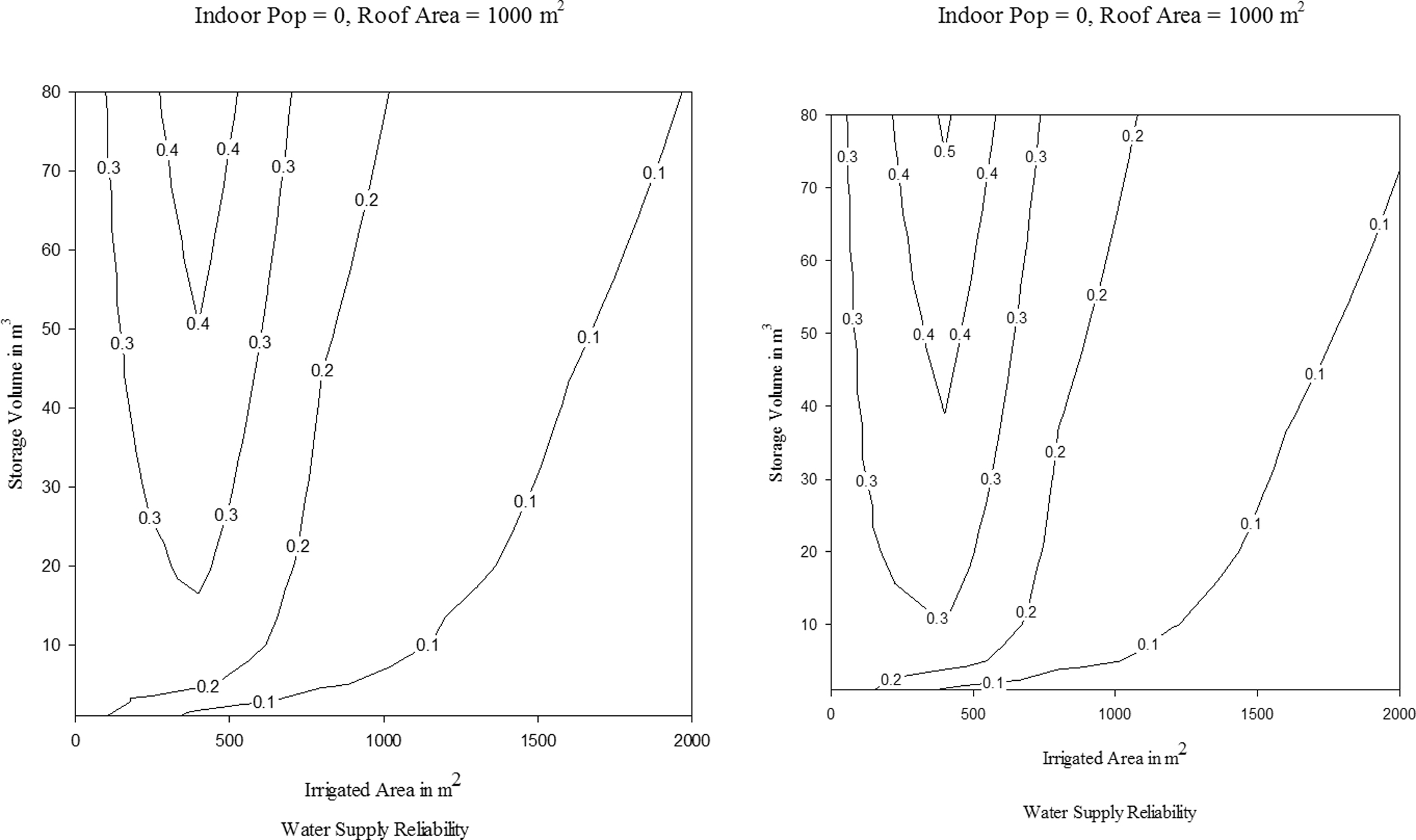

Supplement: Supplementary file 4 — Supplementary material [file mmc4.zip › C29.jpg]

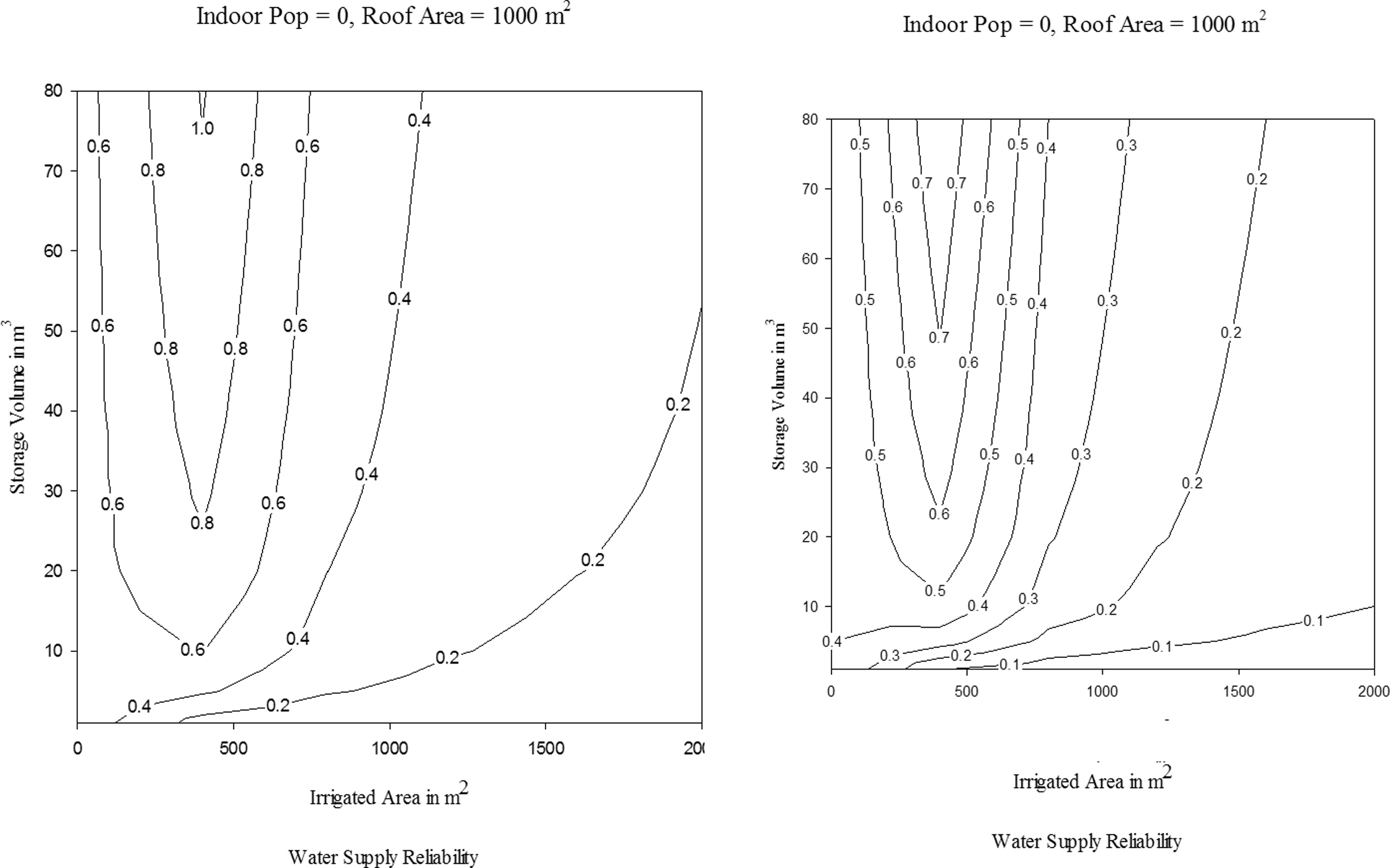

Supplement: Supplementary file 4 — Supplementary material [file mmc4.zip › C3.jpg]

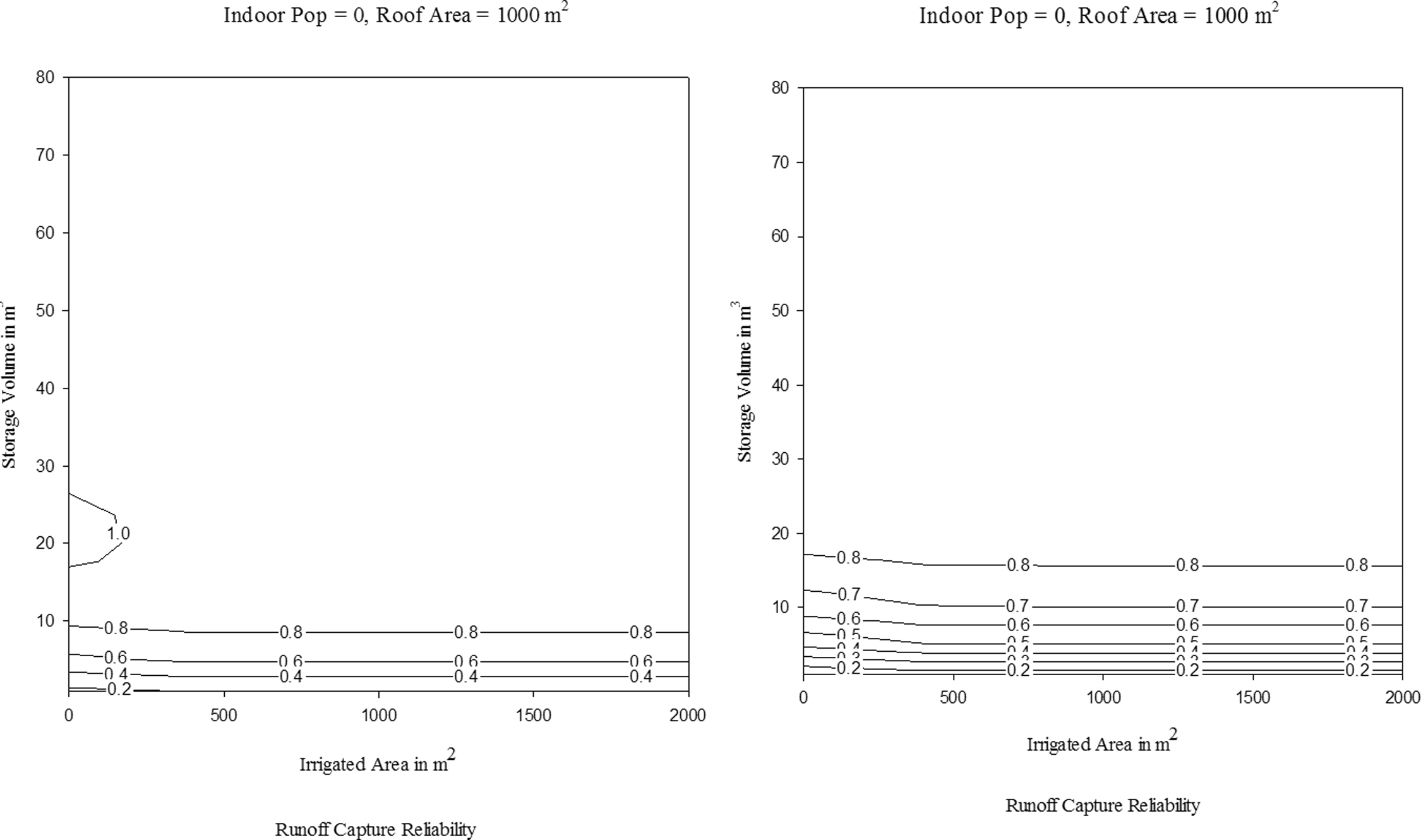

Supplement: Supplementary file 4 — Supplementary material [file mmc4.zip › C30.jpg]

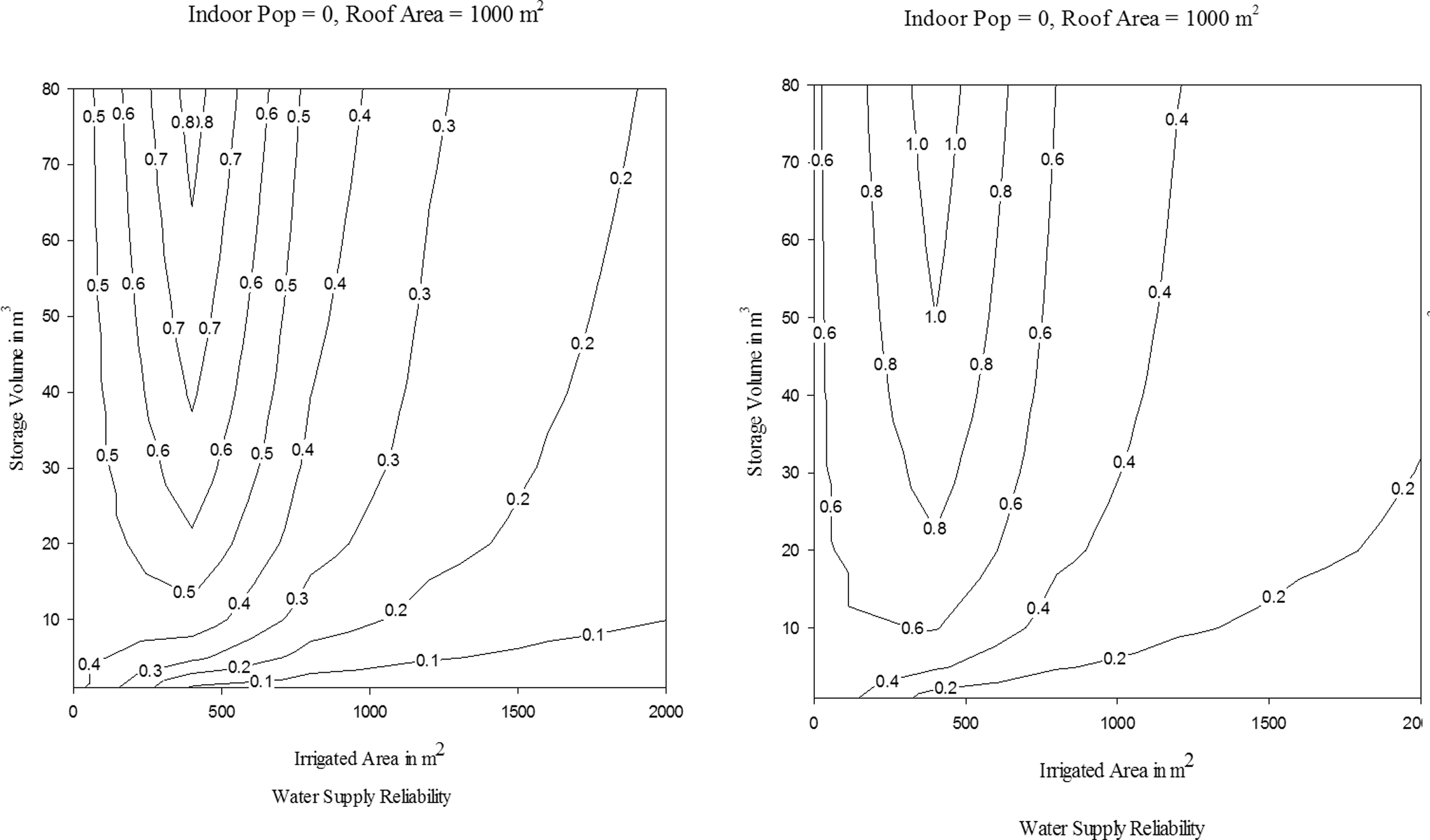

Supplement: Supplementary file 4 — Supplementary material [file mmc4.zip › C31.jpg]

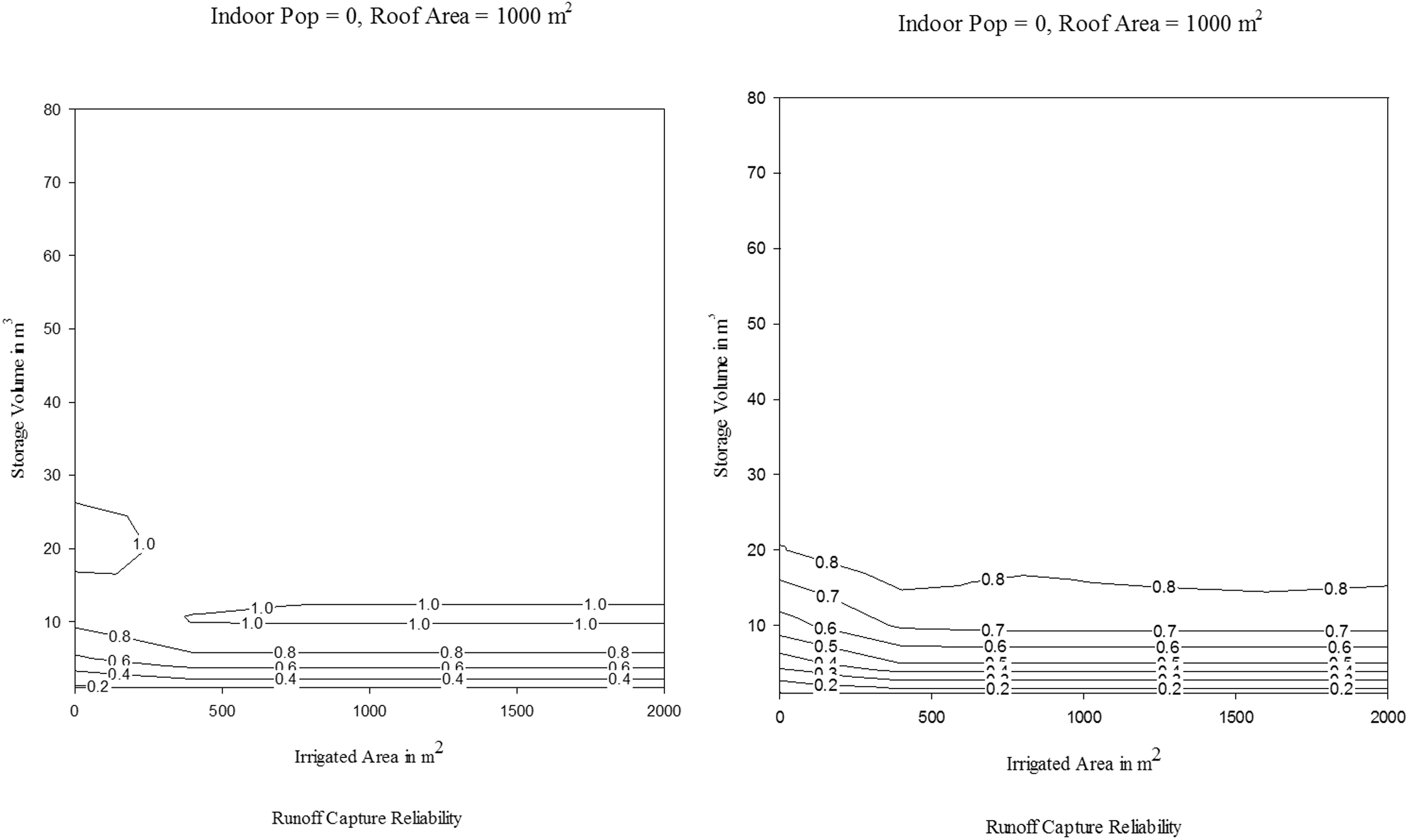

Supplement: Supplementary file 4 — Supplementary material [file mmc4.zip › C32.jpg]

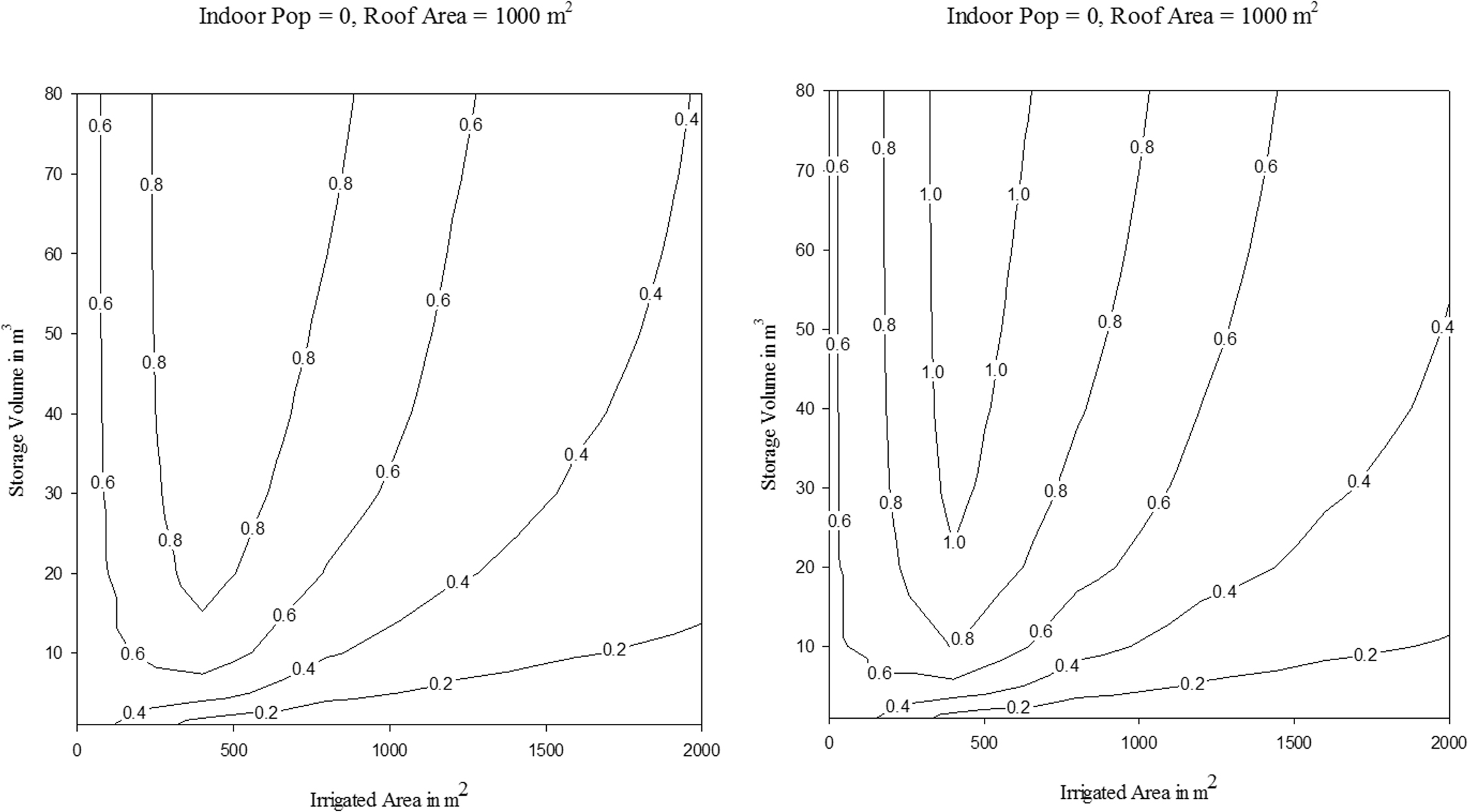

Supplement: Supplementary file 4 — Supplementary material [file mmc4.zip › C33.jpg]

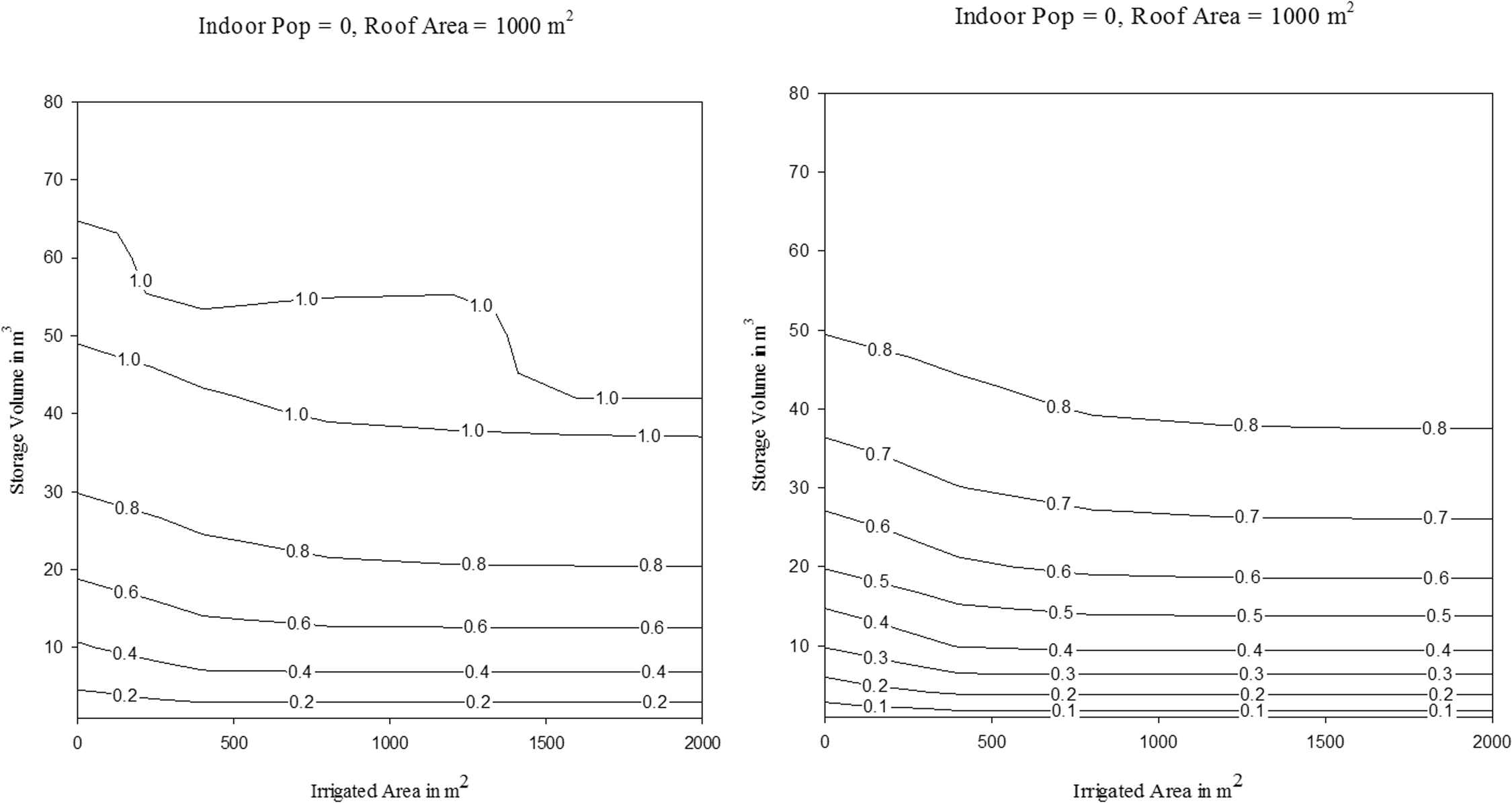

Supplement: Supplementary file 4 — Supplementary material [file mmc4.zip › C34.jpg]

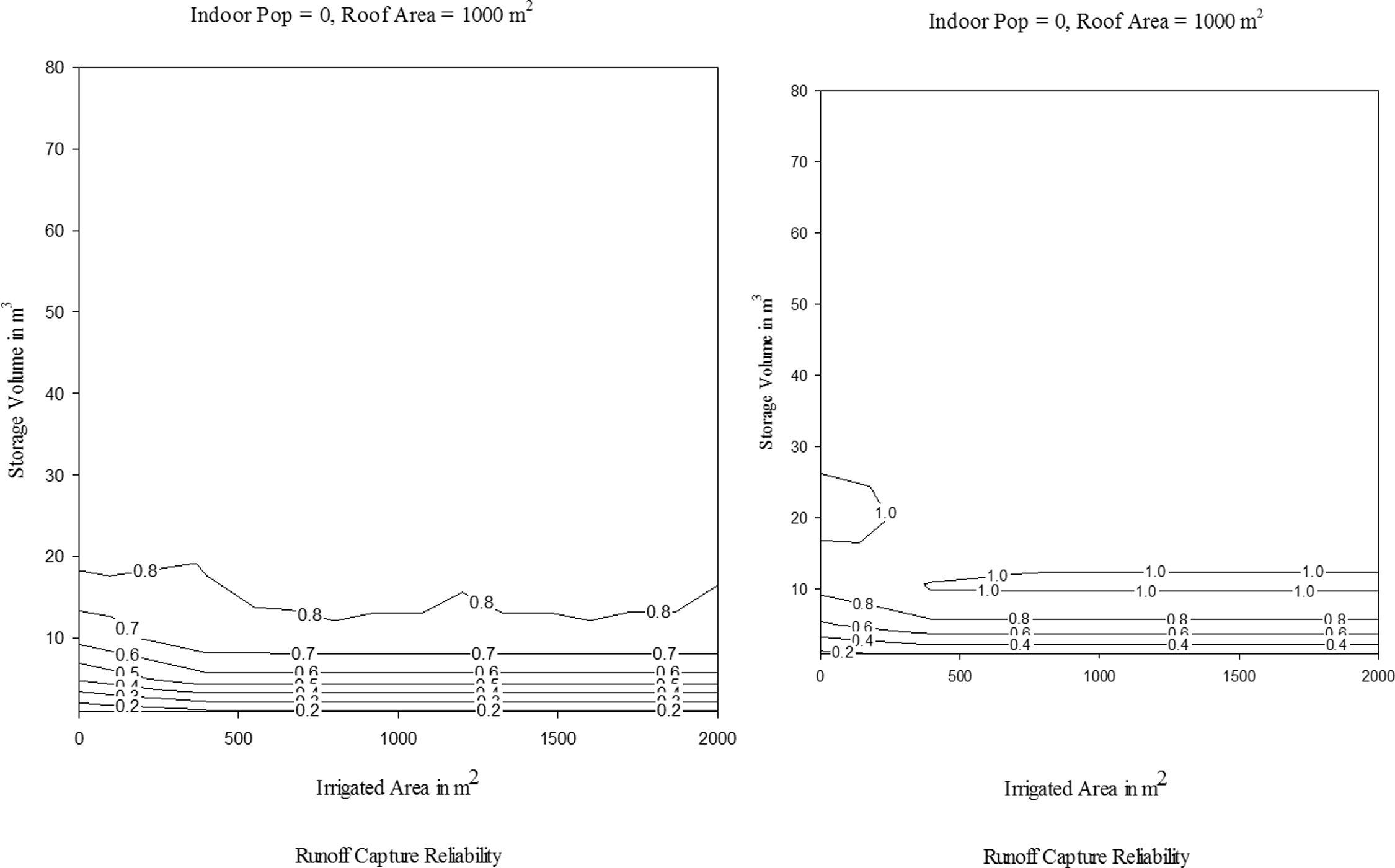

Supplement: Supplementary file 4 — Supplementary material [file mmc4.zip › C4.jpg]

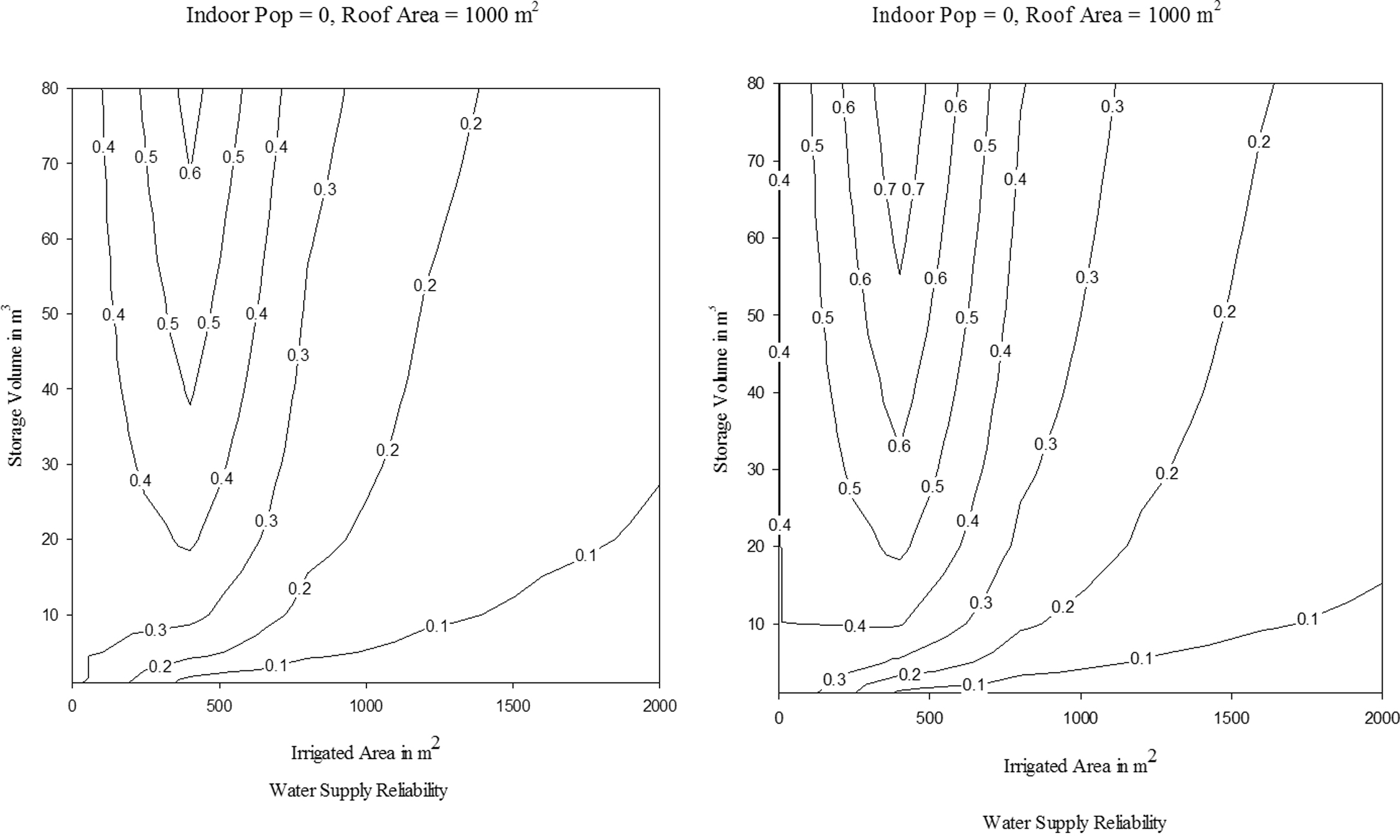

Supplement: Supplementary file 4 — Supplementary material [file mmc4.zip › C5.jpg]

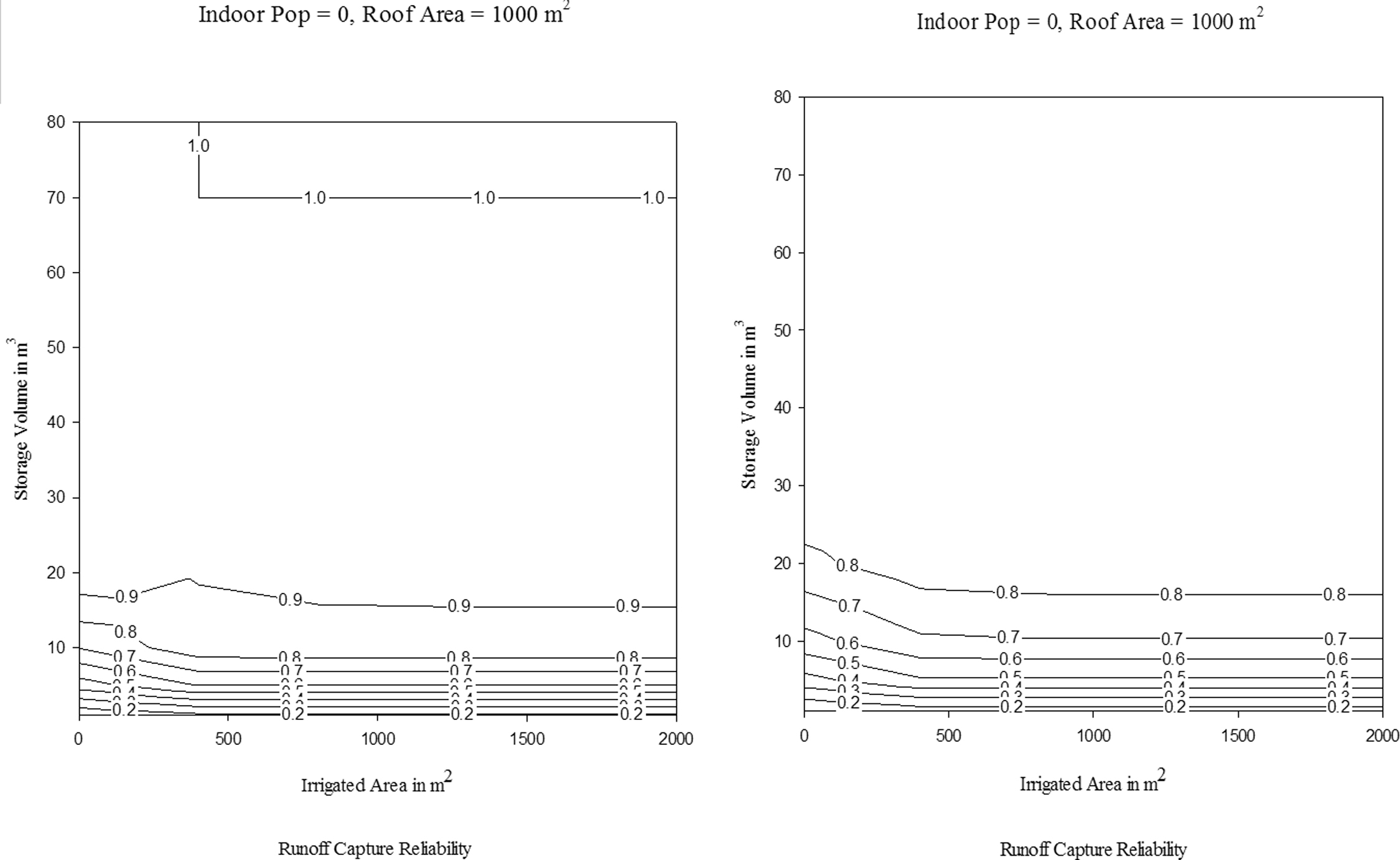

Supplement: Supplementary file 4 — Supplementary material [file mmc4.zip › C6.jpg]

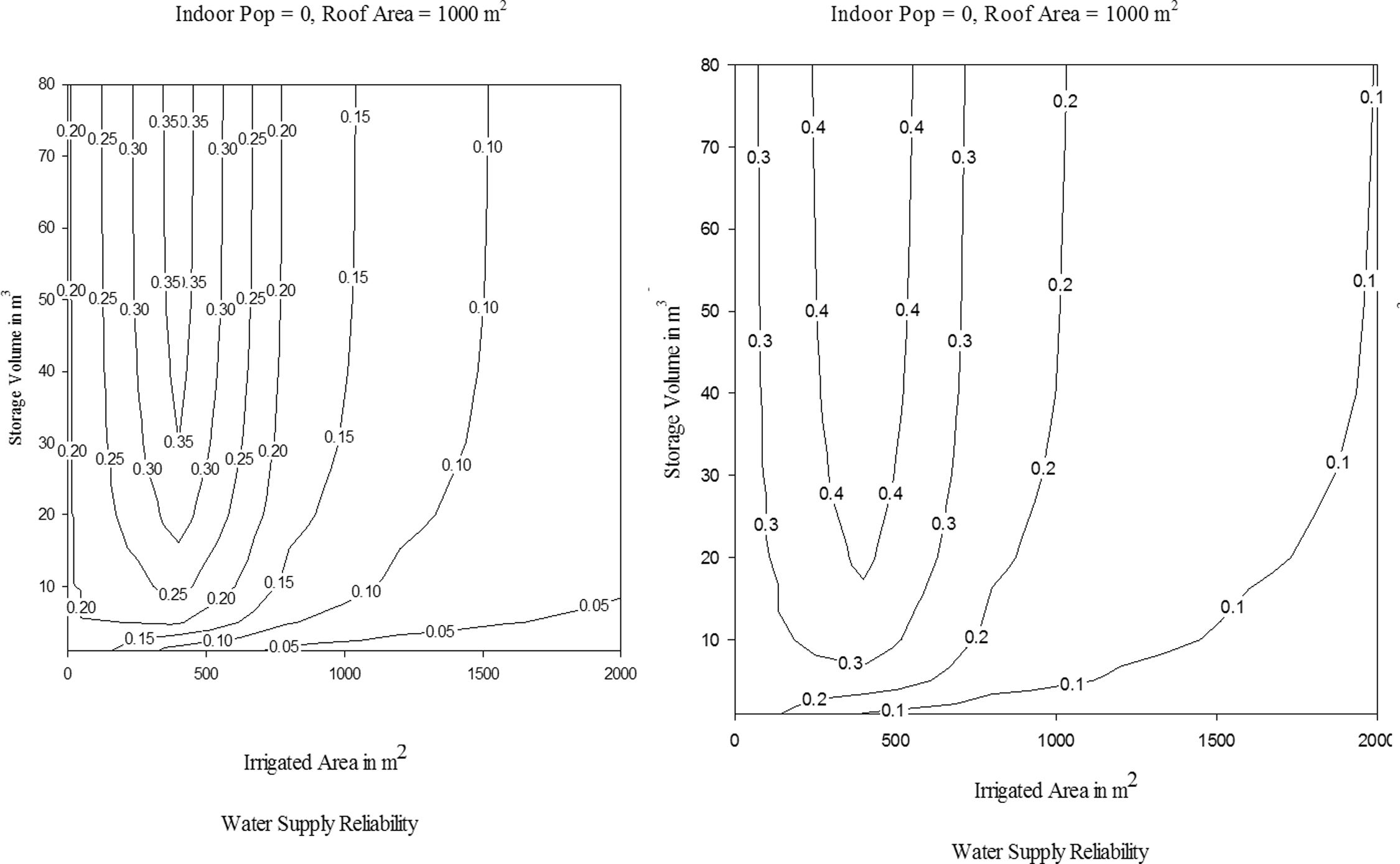

Supplement: Supplementary file 4 — Supplementary material [file mmc4.zip › C7.jpg]

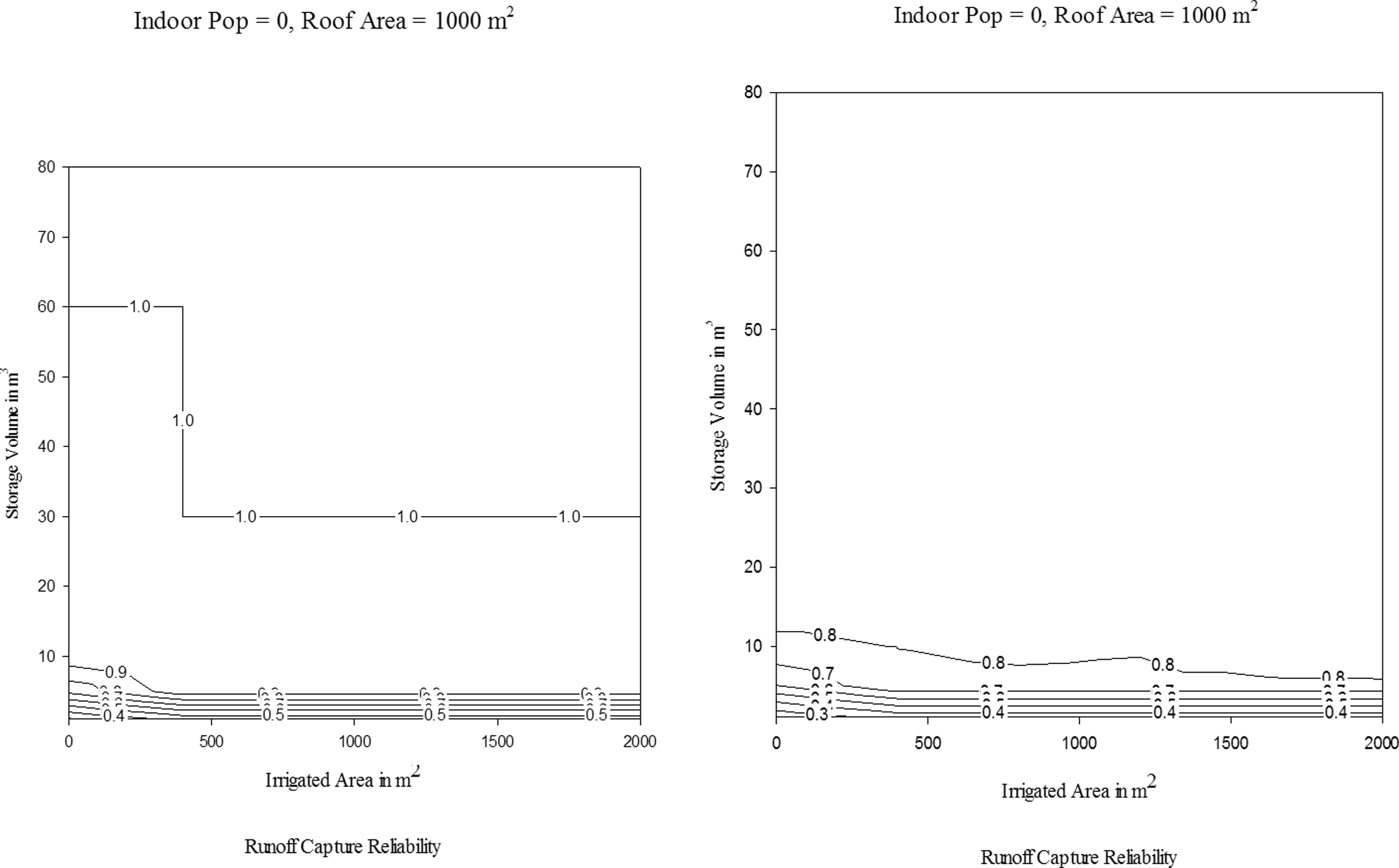

Supplement: Supplementary file 4 — Supplementary material [file mmc4.zip › C8.jpg]

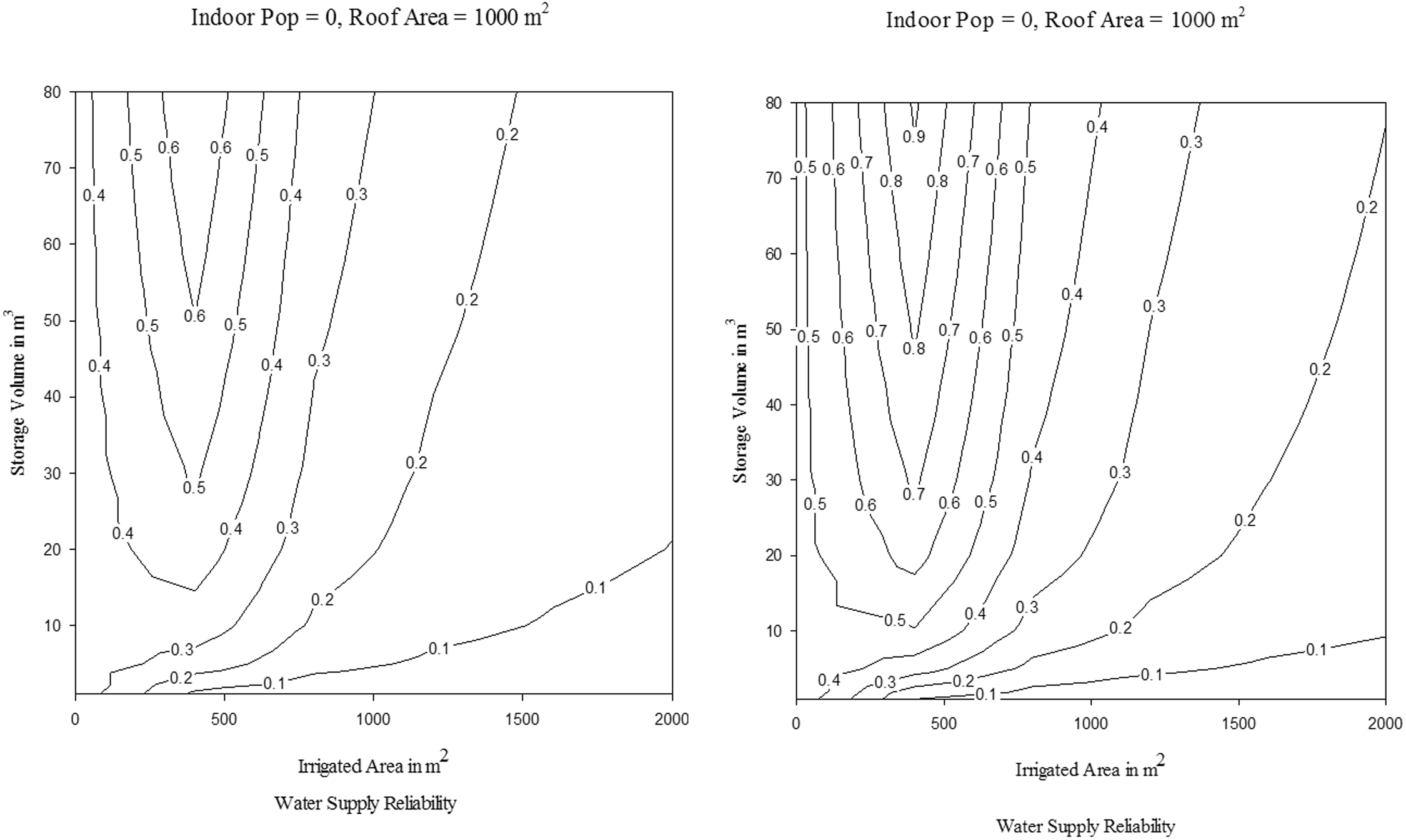

Supplement: Supplementary file 4 — Supplementary material [file mmc4.zip › C9.jpg]

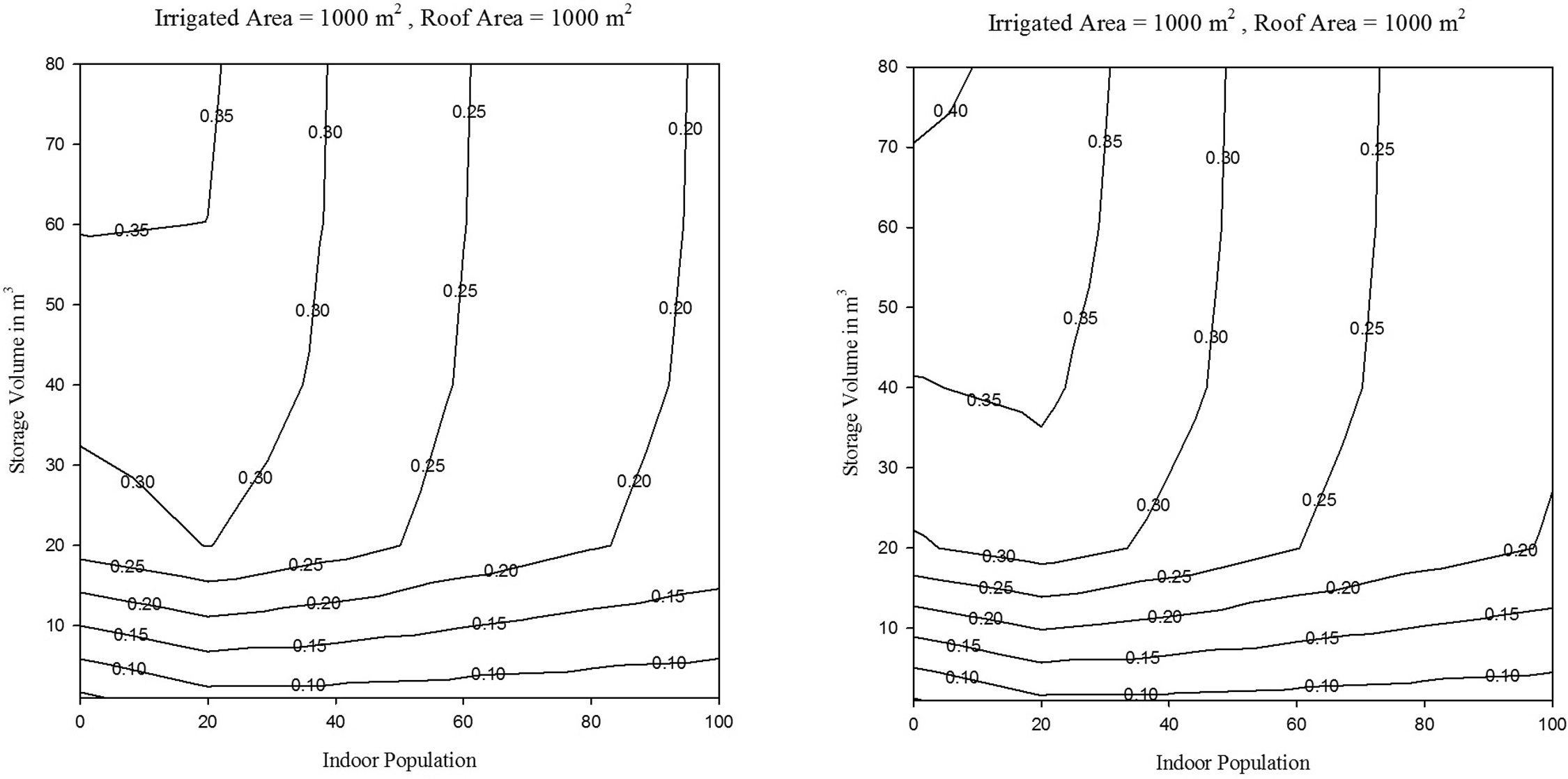

Supplement: Supplementary file 5 — Supplementary material [file mmc5.zip › D1.jpg]

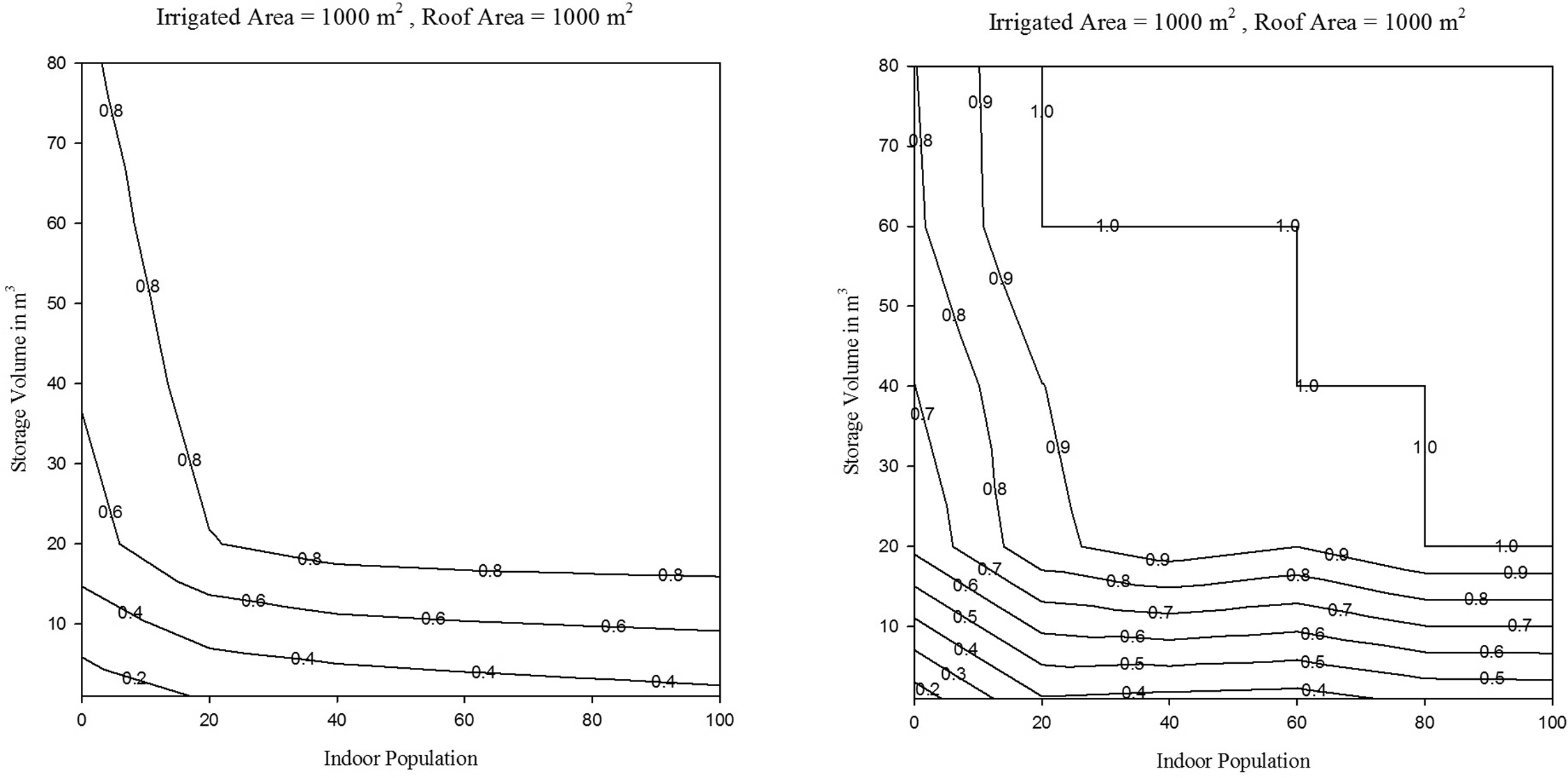

Supplement: Supplementary file 5 — Supplementary material [file mmc5.zip › D10.jpg]

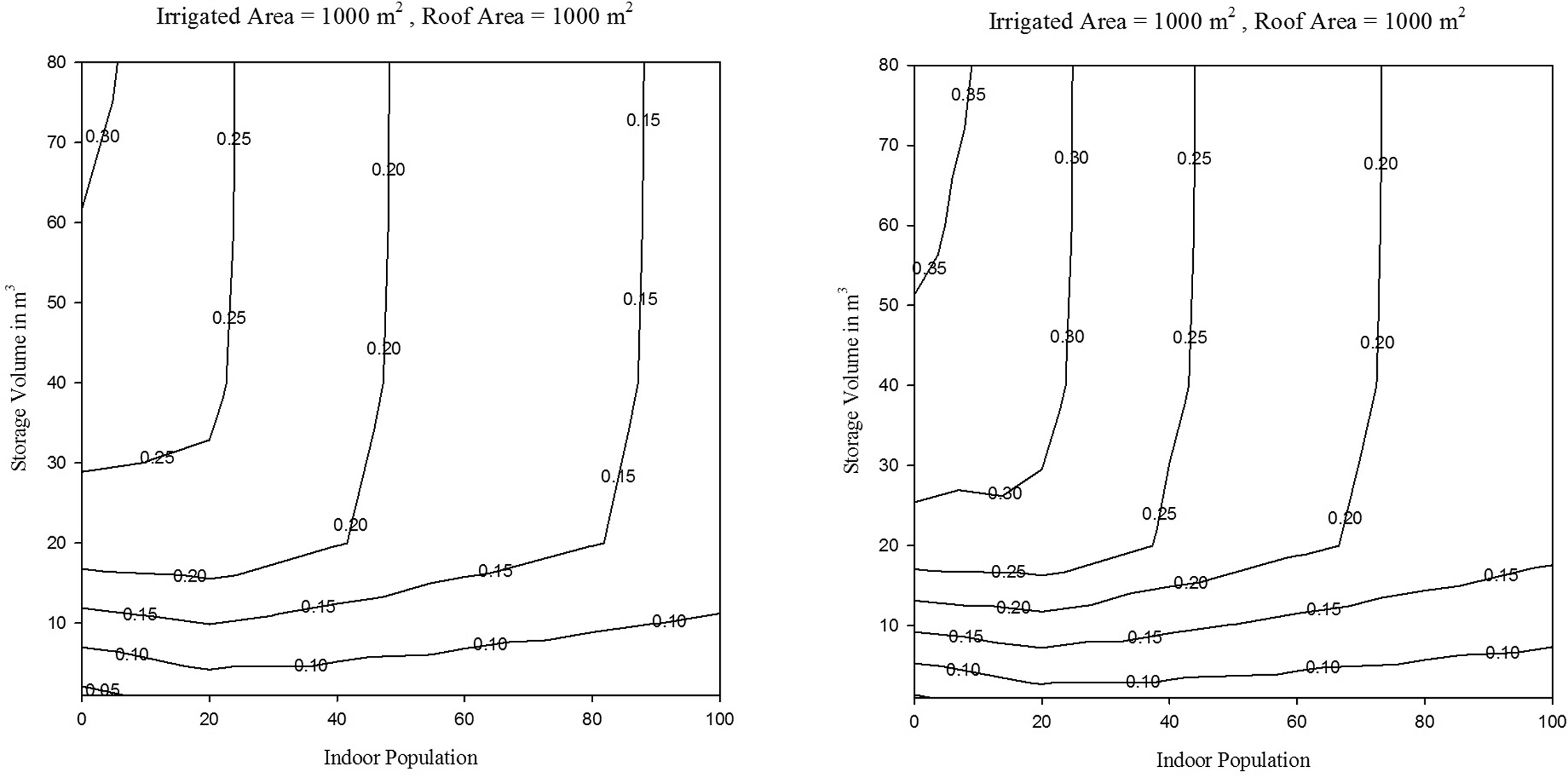

Supplement: Supplementary file 5 — Supplementary material [file mmc5.zip › D11.jpg]

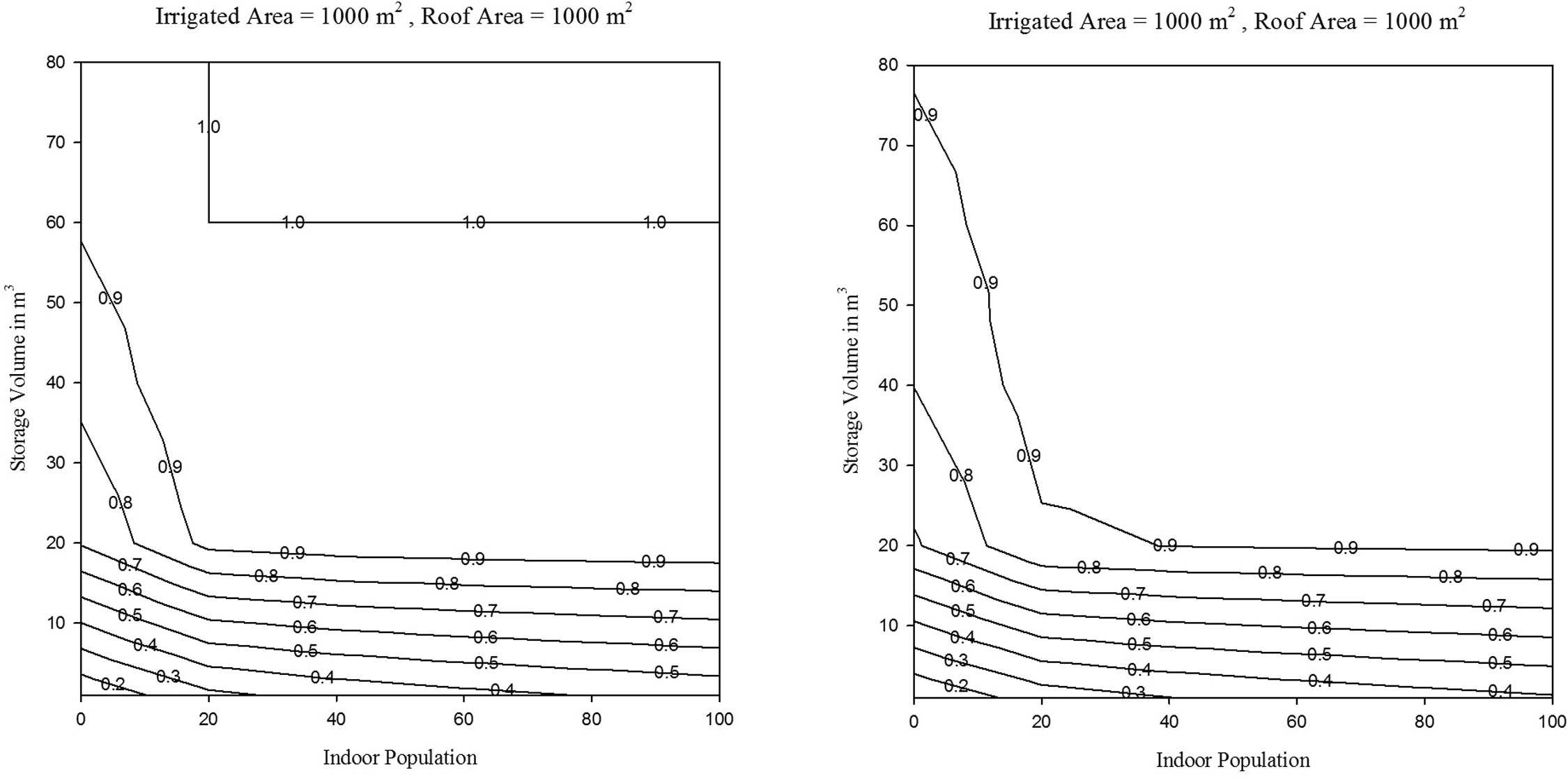

Supplement: Supplementary file 5 — Supplementary material [file mmc5.zip › D12.jpg]

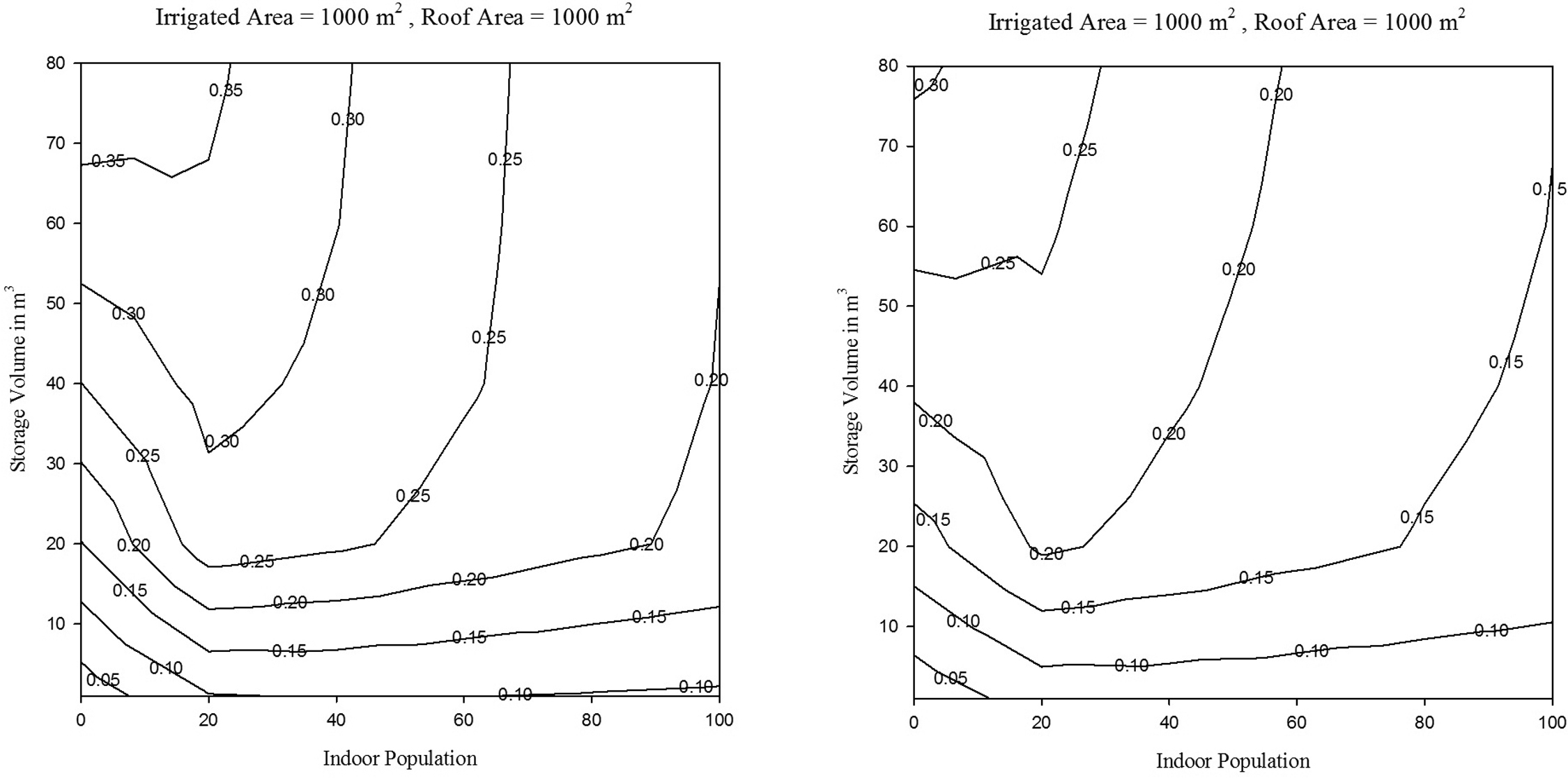

Supplement: Supplementary file 5 — Supplementary material [file mmc5.zip › D13.jpg]

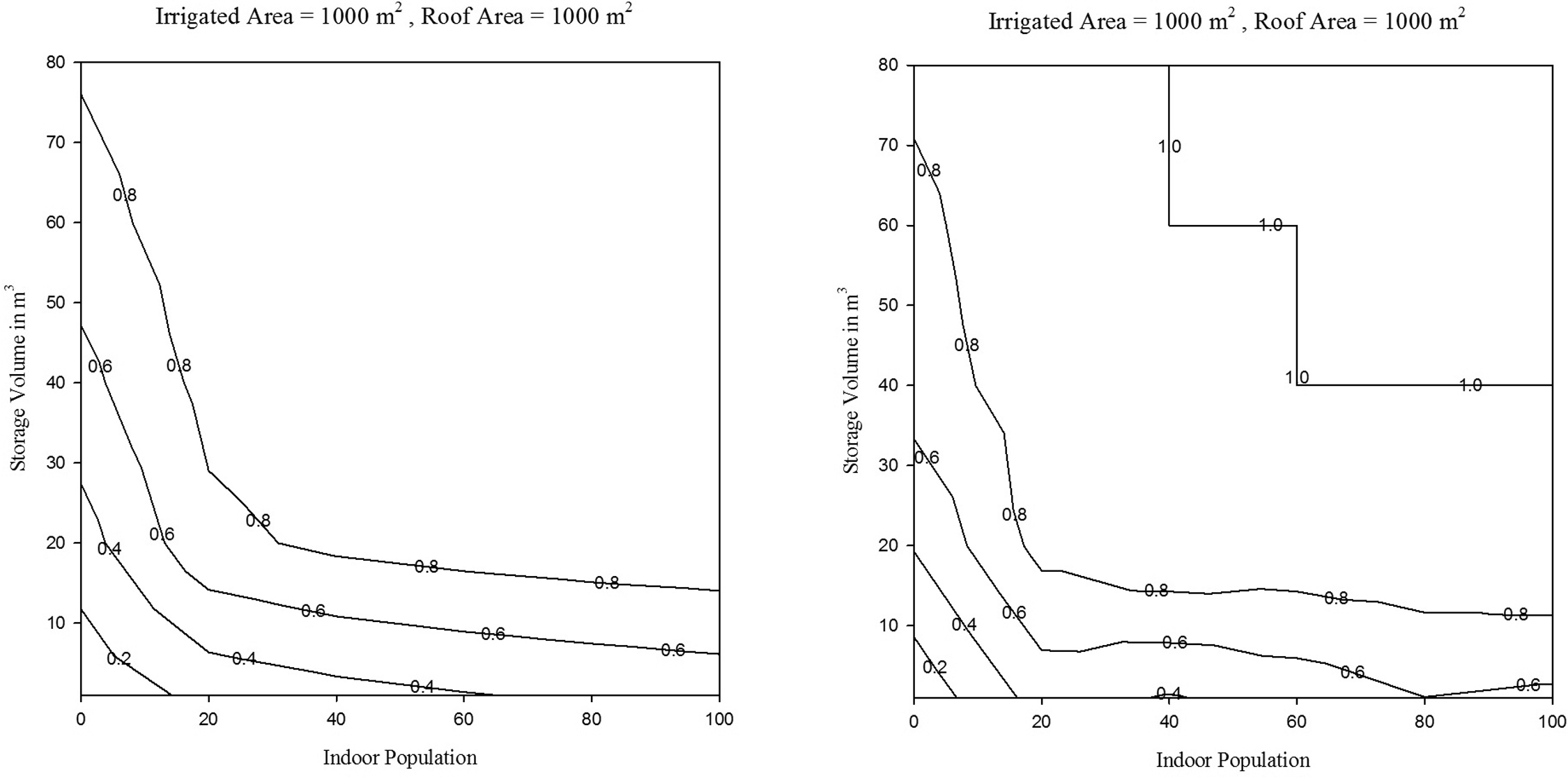

Supplement: Supplementary file 5 — Supplementary material [file mmc5.zip › D14.jpg]

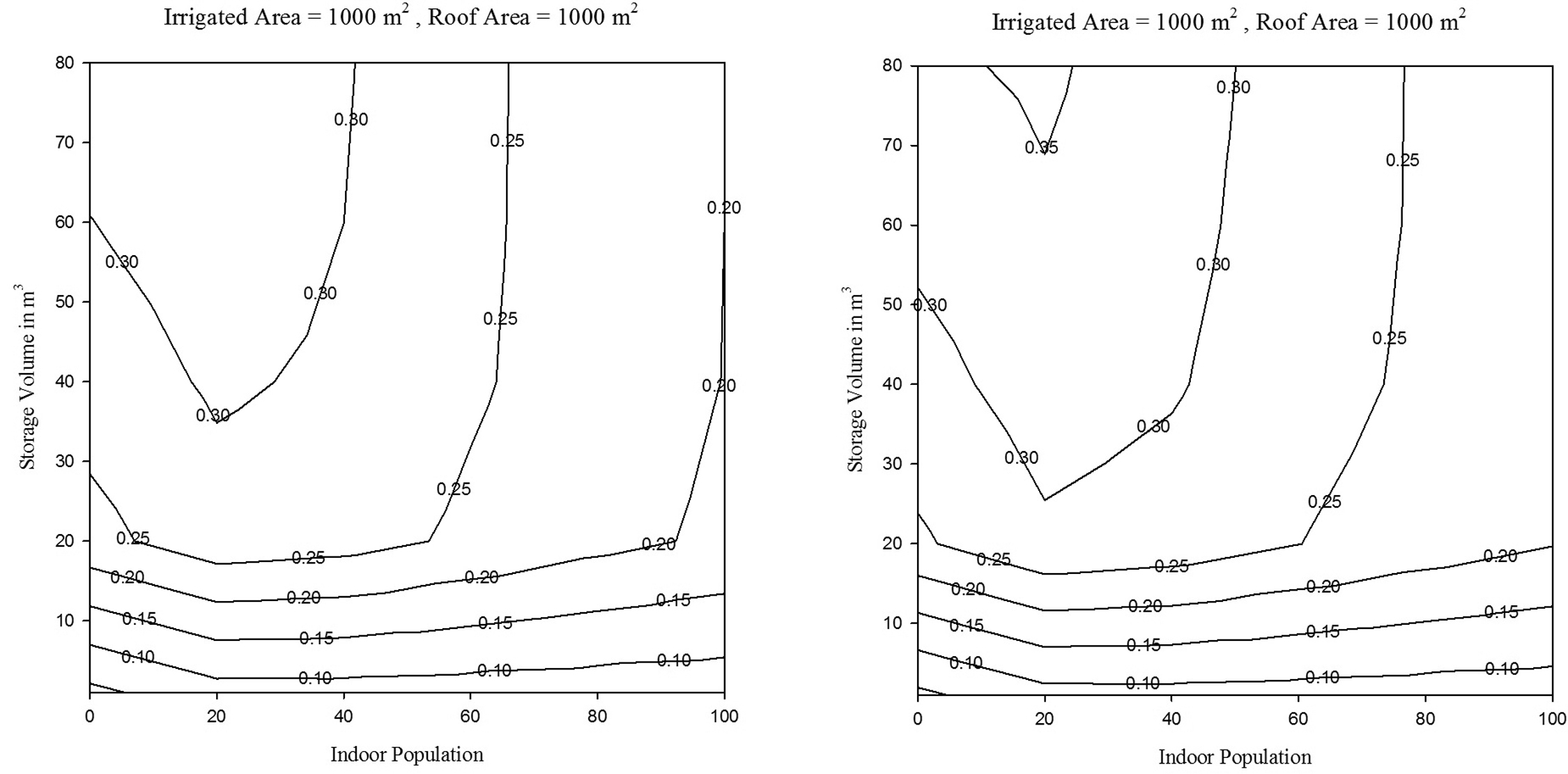

Supplement: Supplementary file 5 — Supplementary material [file mmc5.zip › D15.jpg]

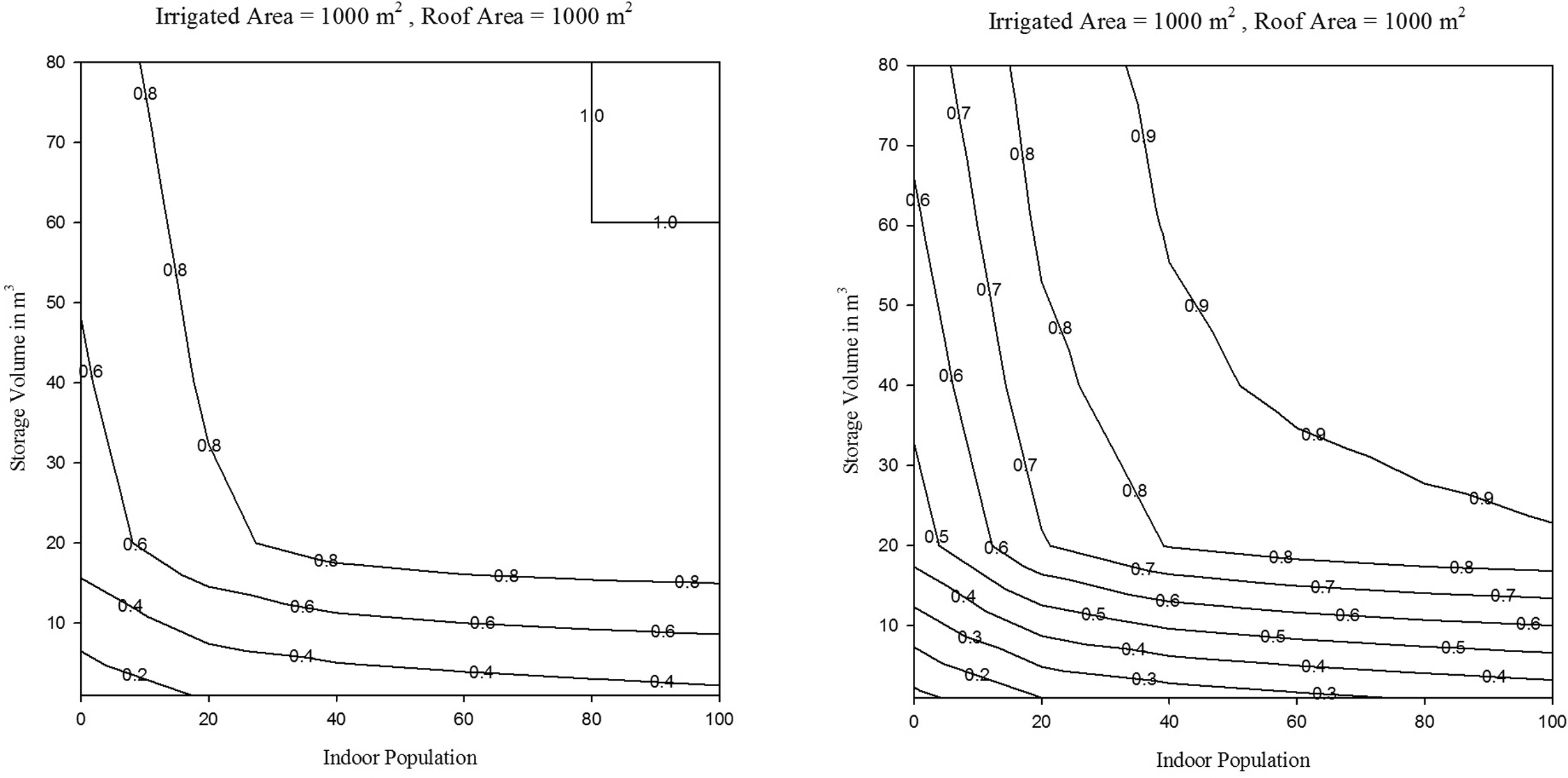

Supplement: Supplementary file 5 — Supplementary material [file mmc5.zip › D16.jpg]

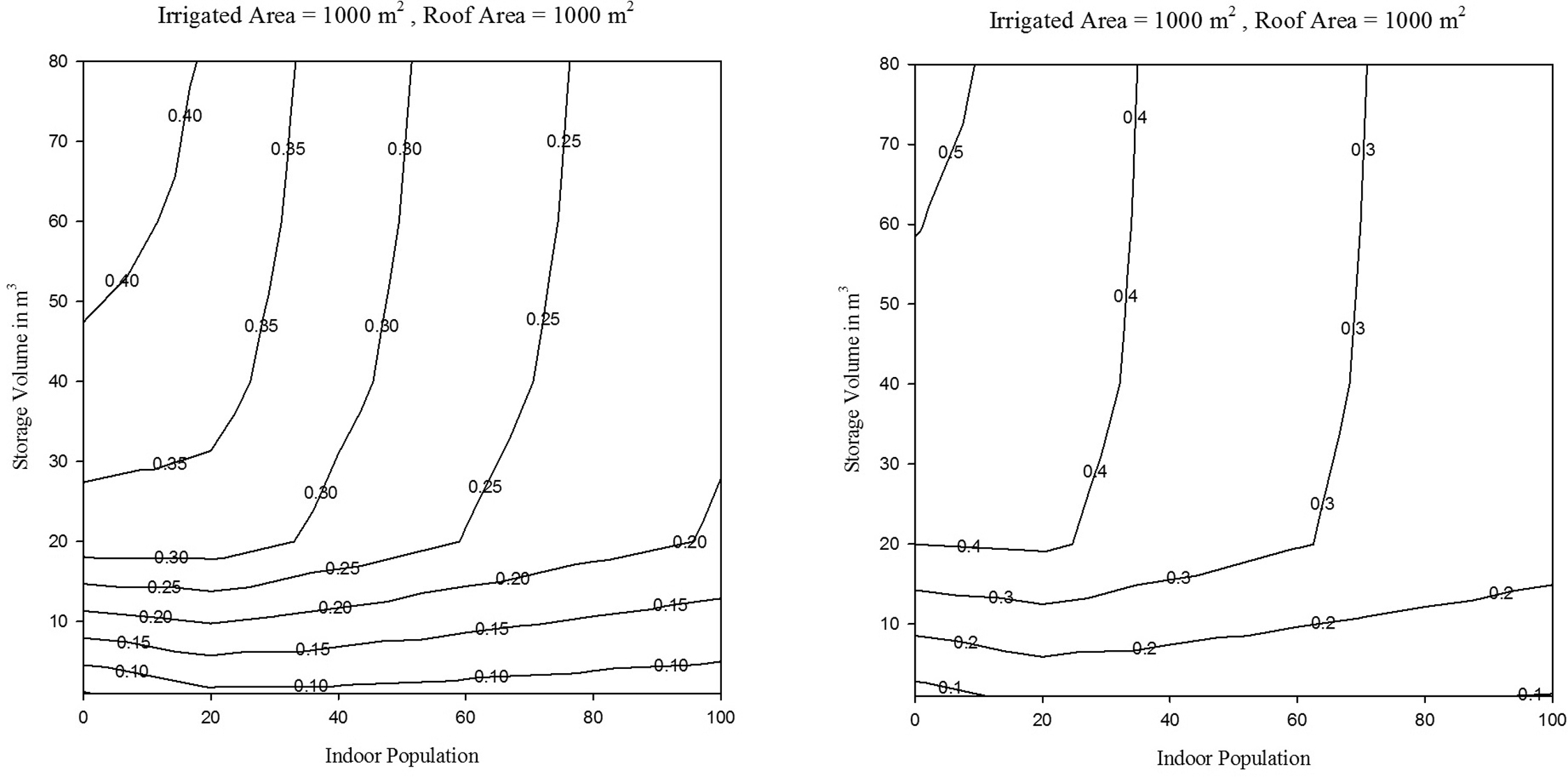

Supplement: Supplementary file 5 — Supplementary material [file mmc5.zip › D17.jpg]

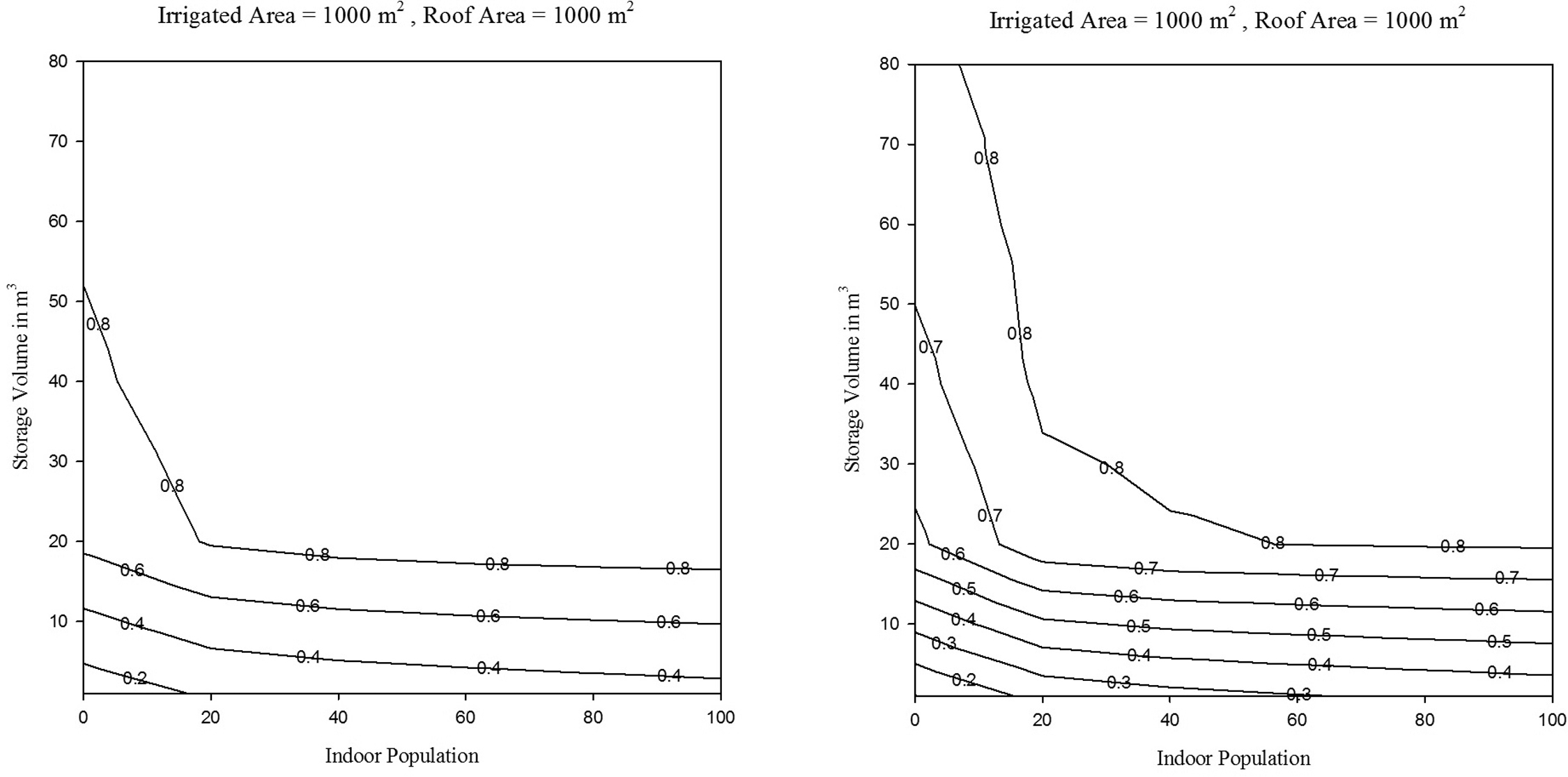

Supplement: Supplementary file 5 — Supplementary material [file mmc5.zip › D18.jpg]

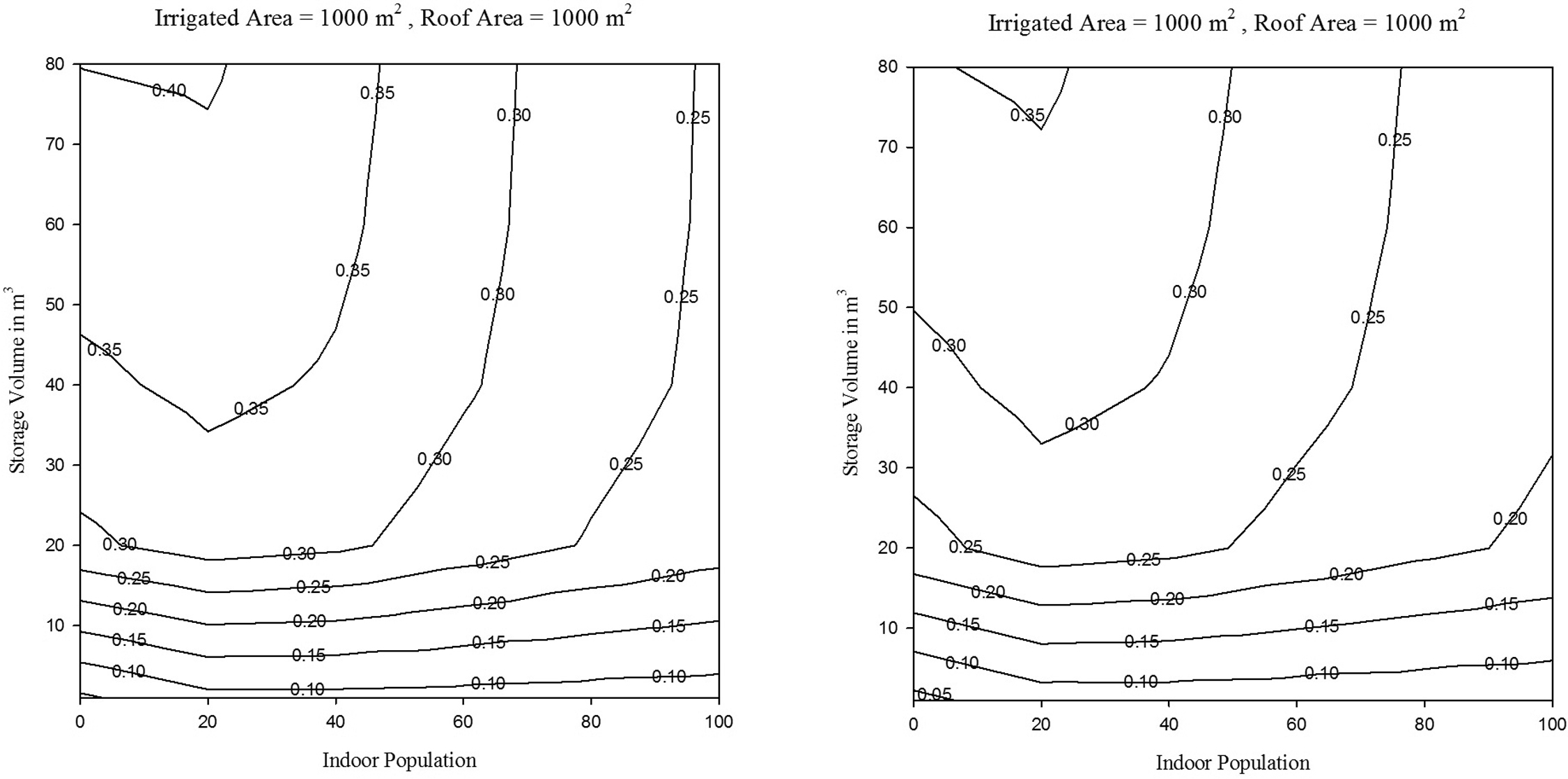

Supplement: Supplementary file 5 — Supplementary material [file mmc5.zip › D19.jpg]

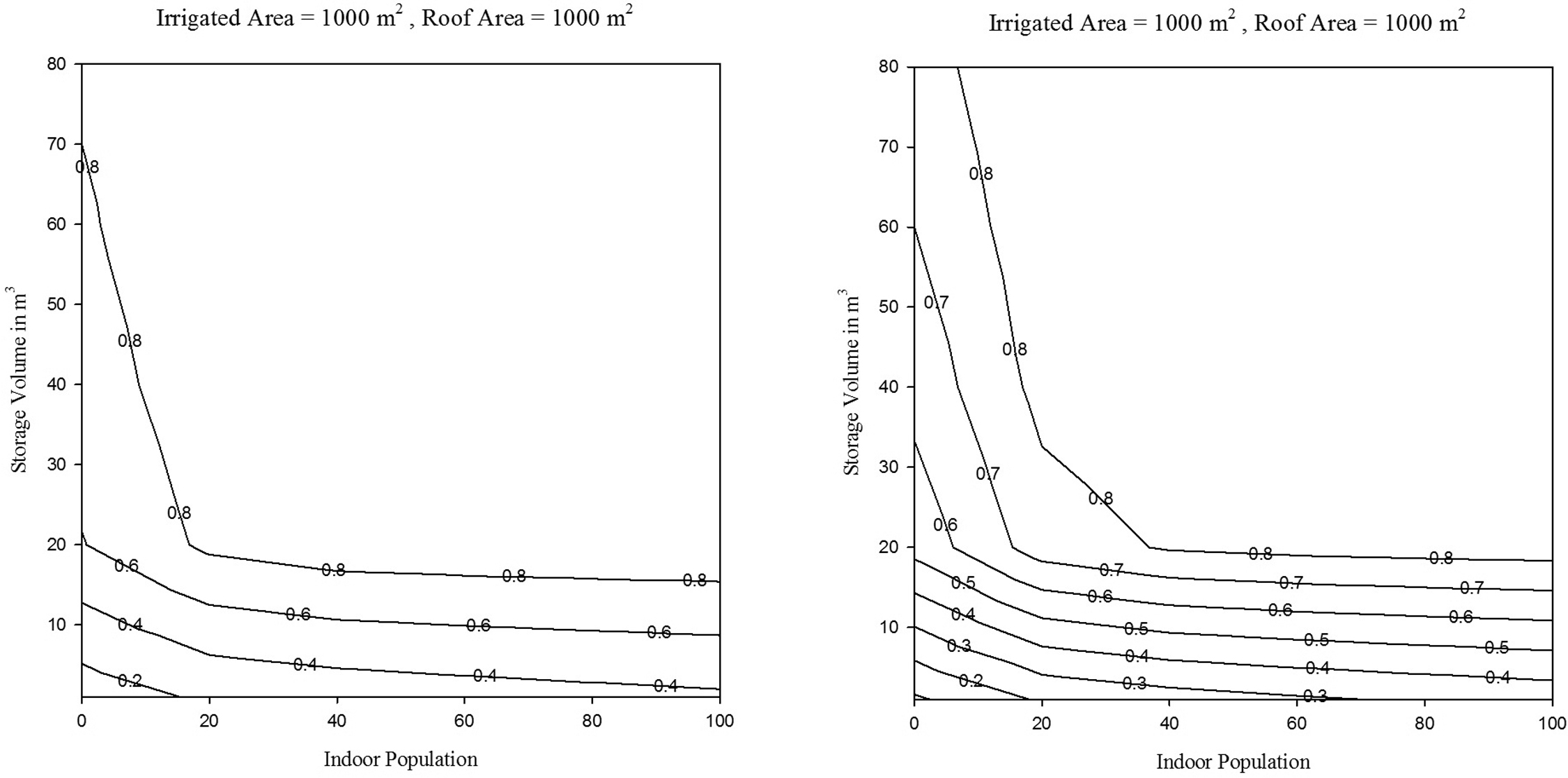

Supplement: Supplementary file 5 — Supplementary material [file mmc5.zip › D2.jpg]

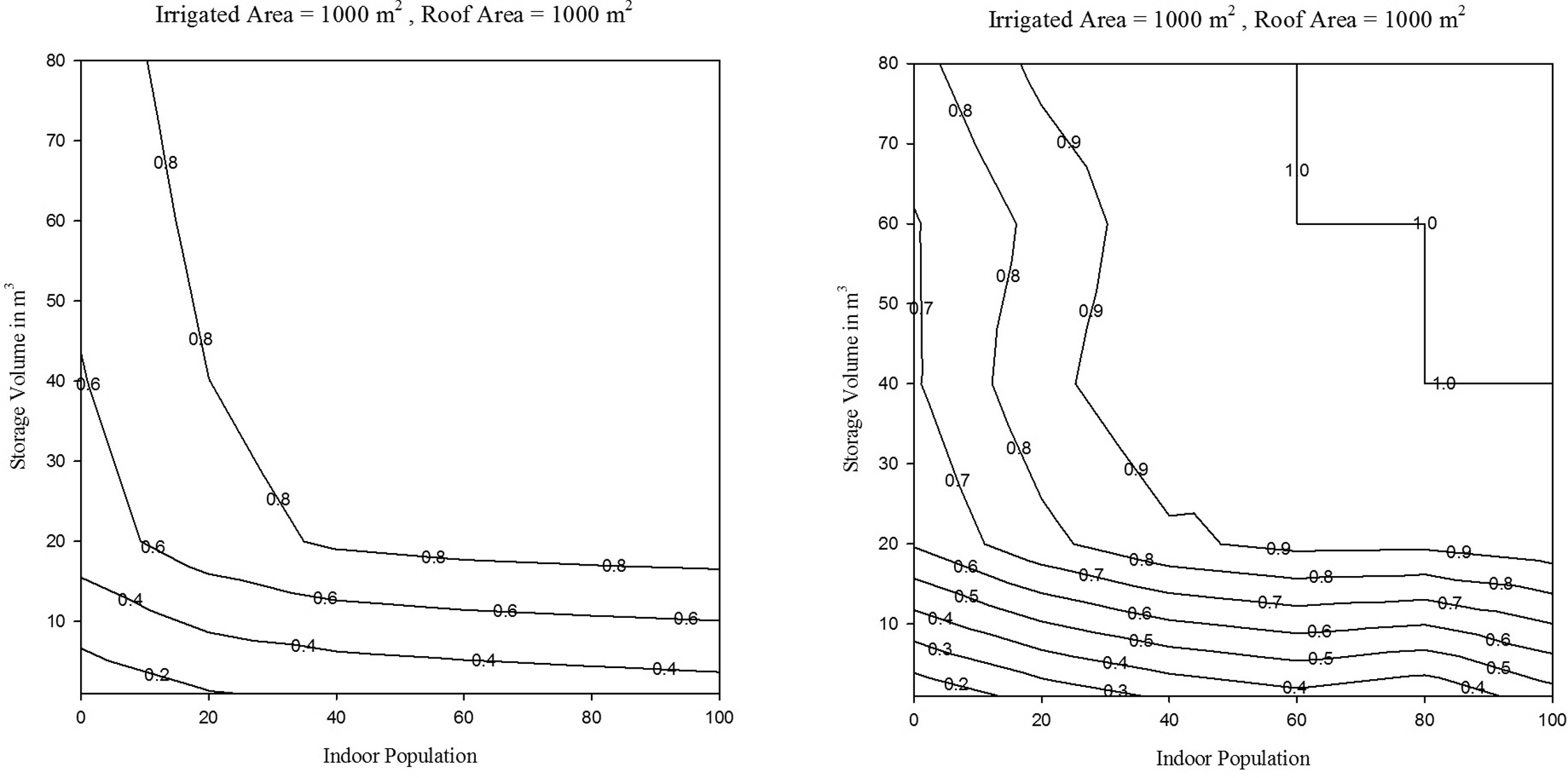

Supplement: Supplementary file 5 — Supplementary material [file mmc5.zip › D20.jpg]

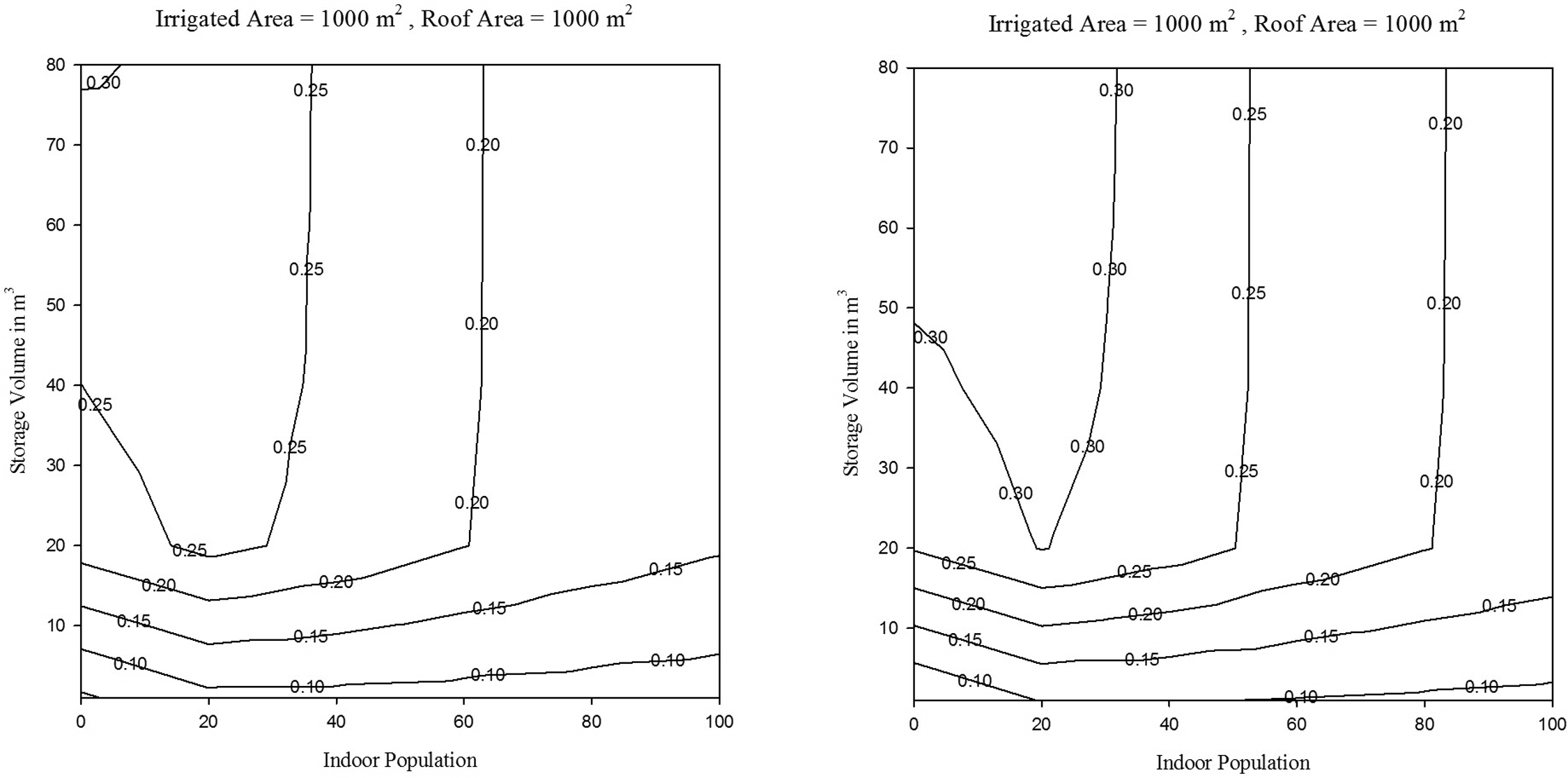

Supplement: Supplementary file 5 — Supplementary material [file mmc5.zip › D21.jpg]

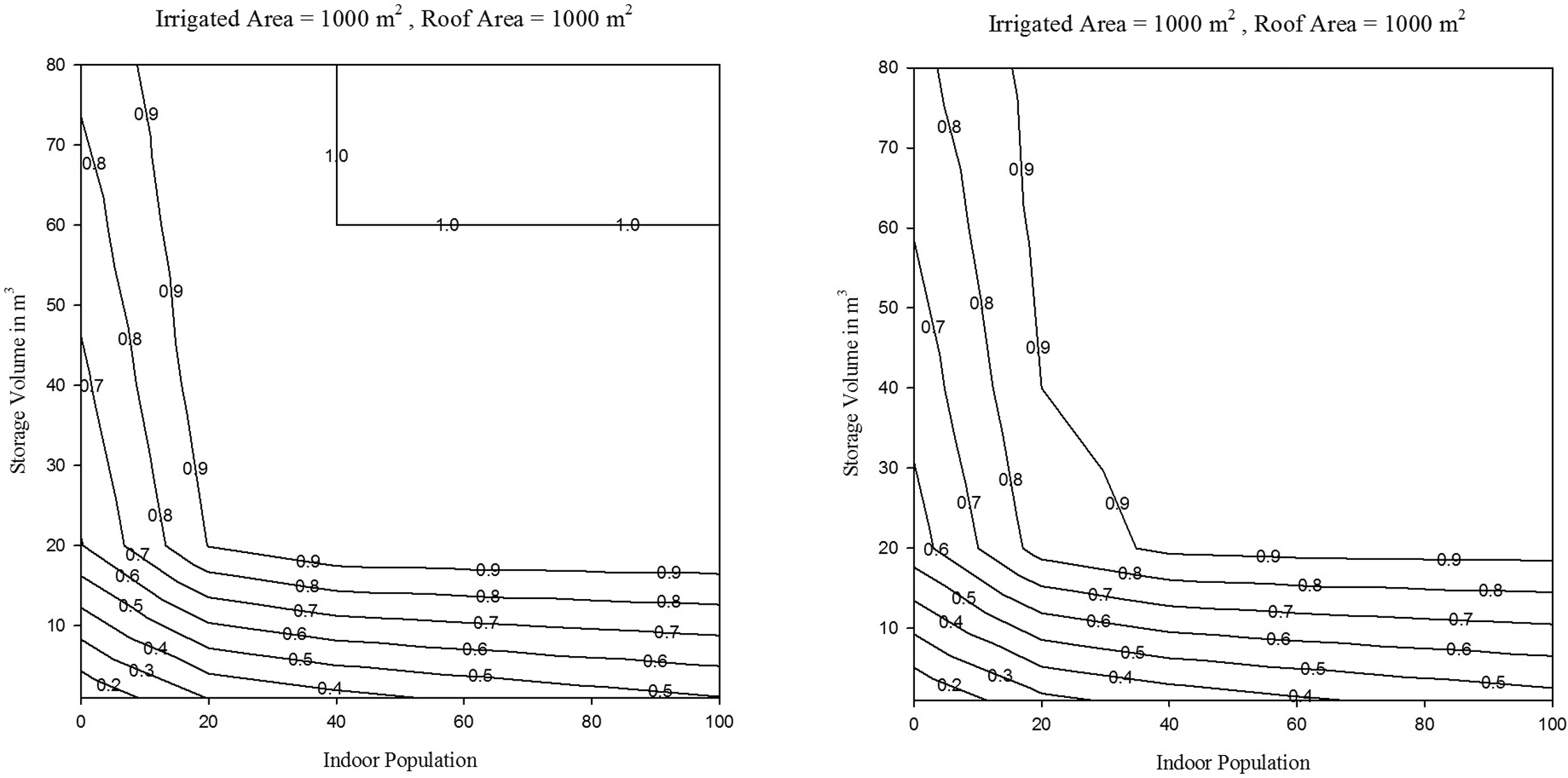

Supplement: Supplementary file 5 — Supplementary material [file mmc5.zip › D22.jpg]

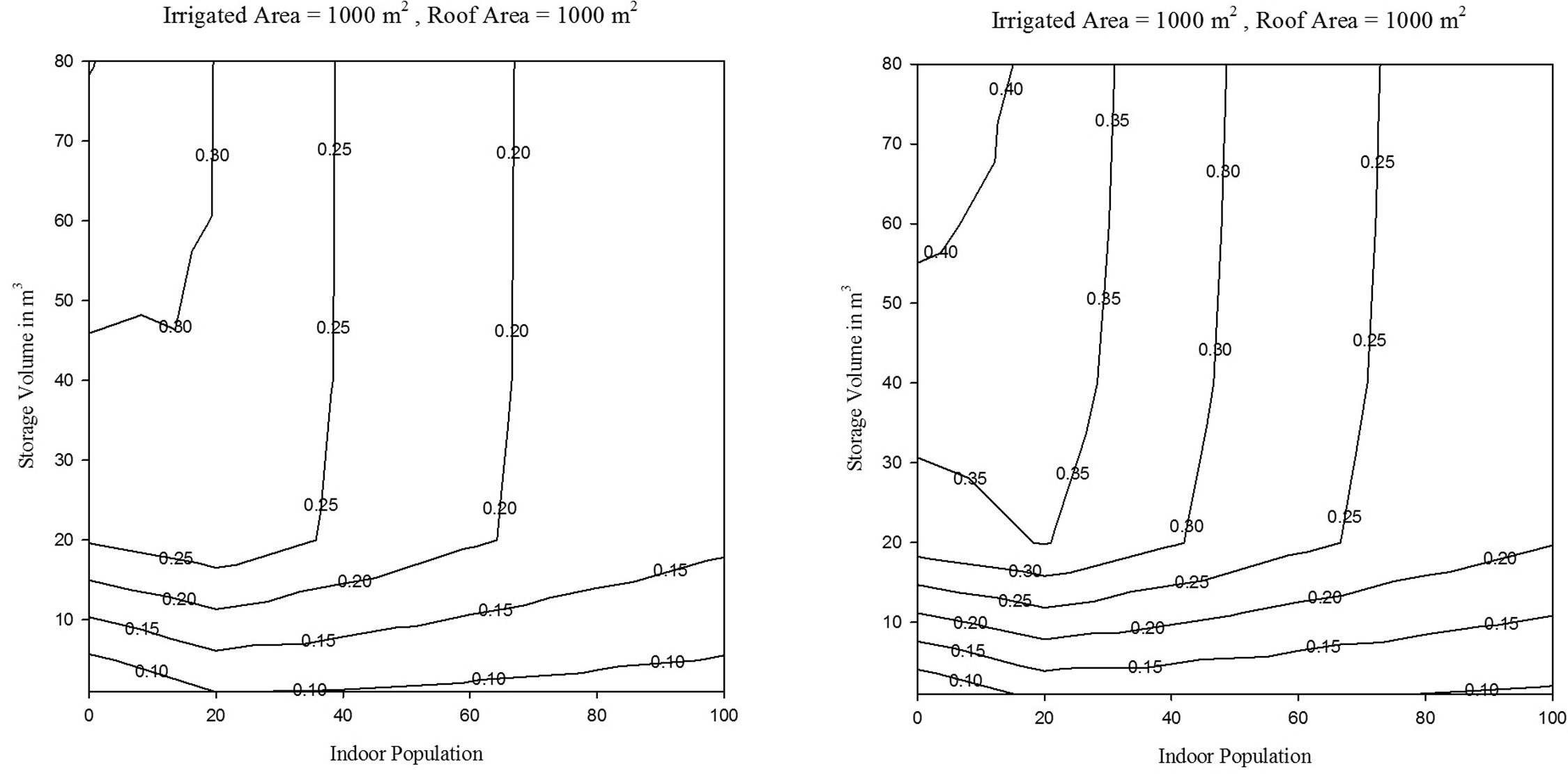

Supplement: Supplementary file 5 — Supplementary material [file mmc5.zip › D23.jpg]

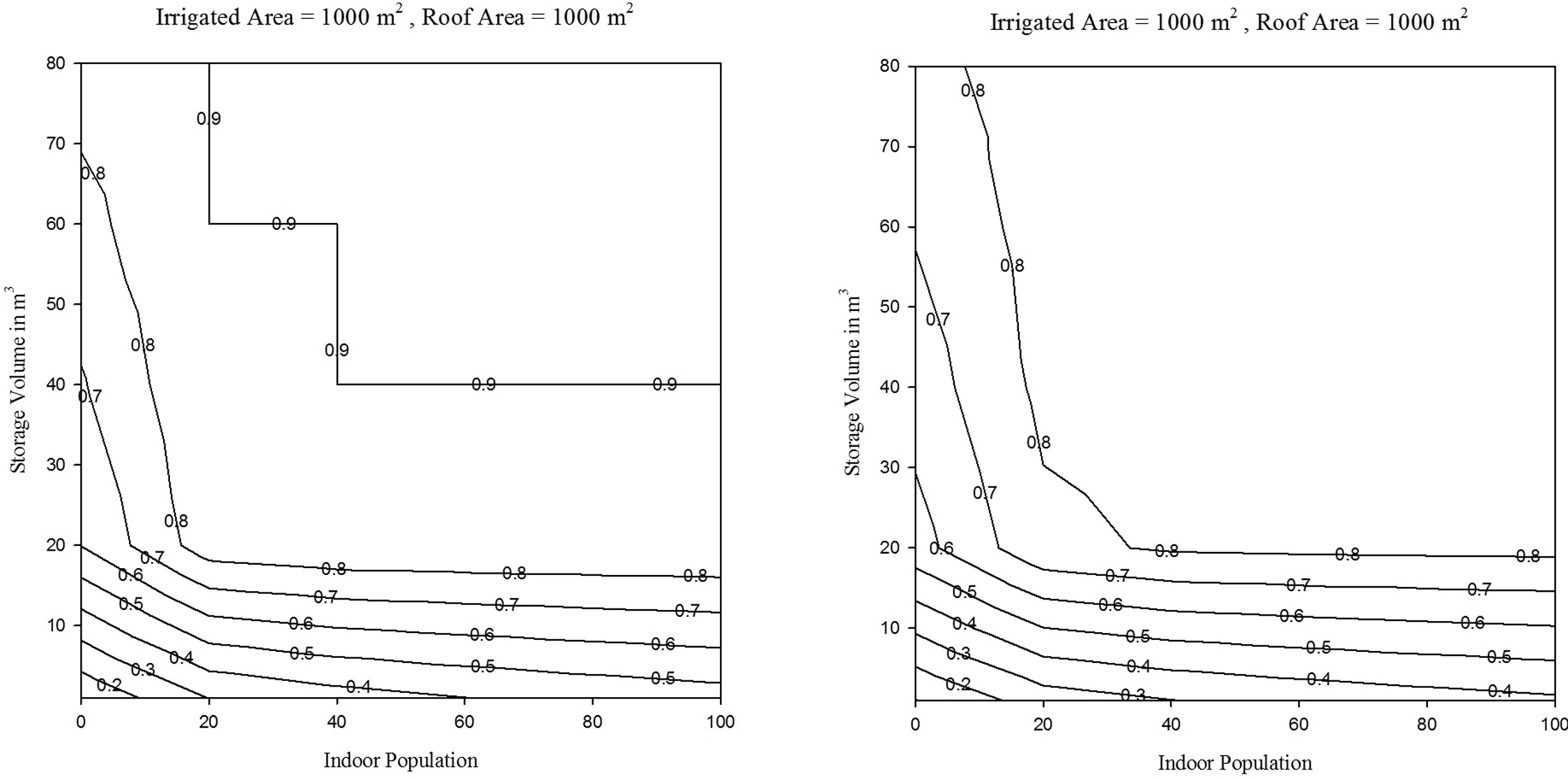

Supplement: Supplementary file 5 — Supplementary material [file mmc5.zip › D24.jpg]

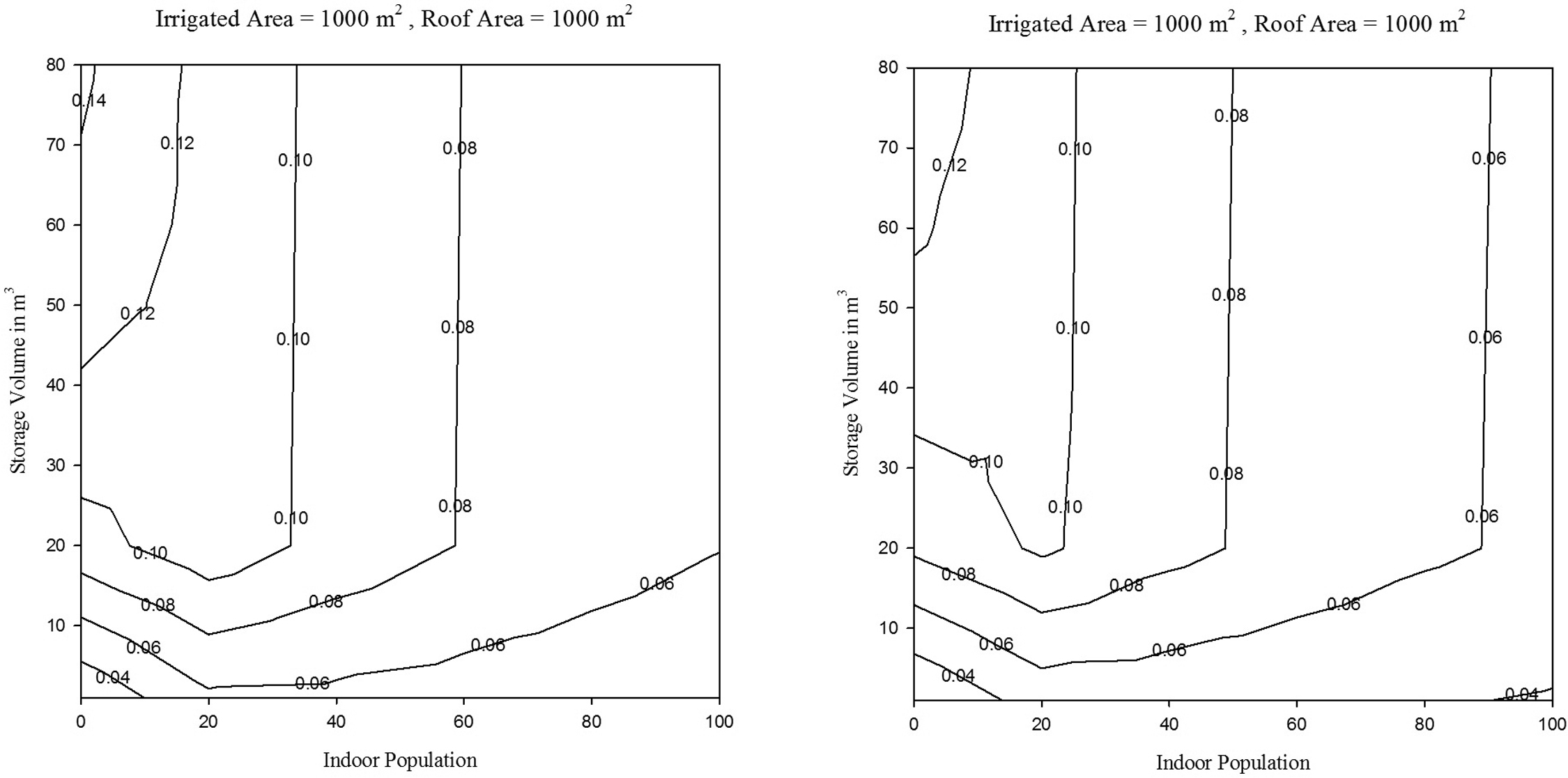

Supplement: Supplementary file 5 — Supplementary material [file mmc5.zip › D25.jpg]

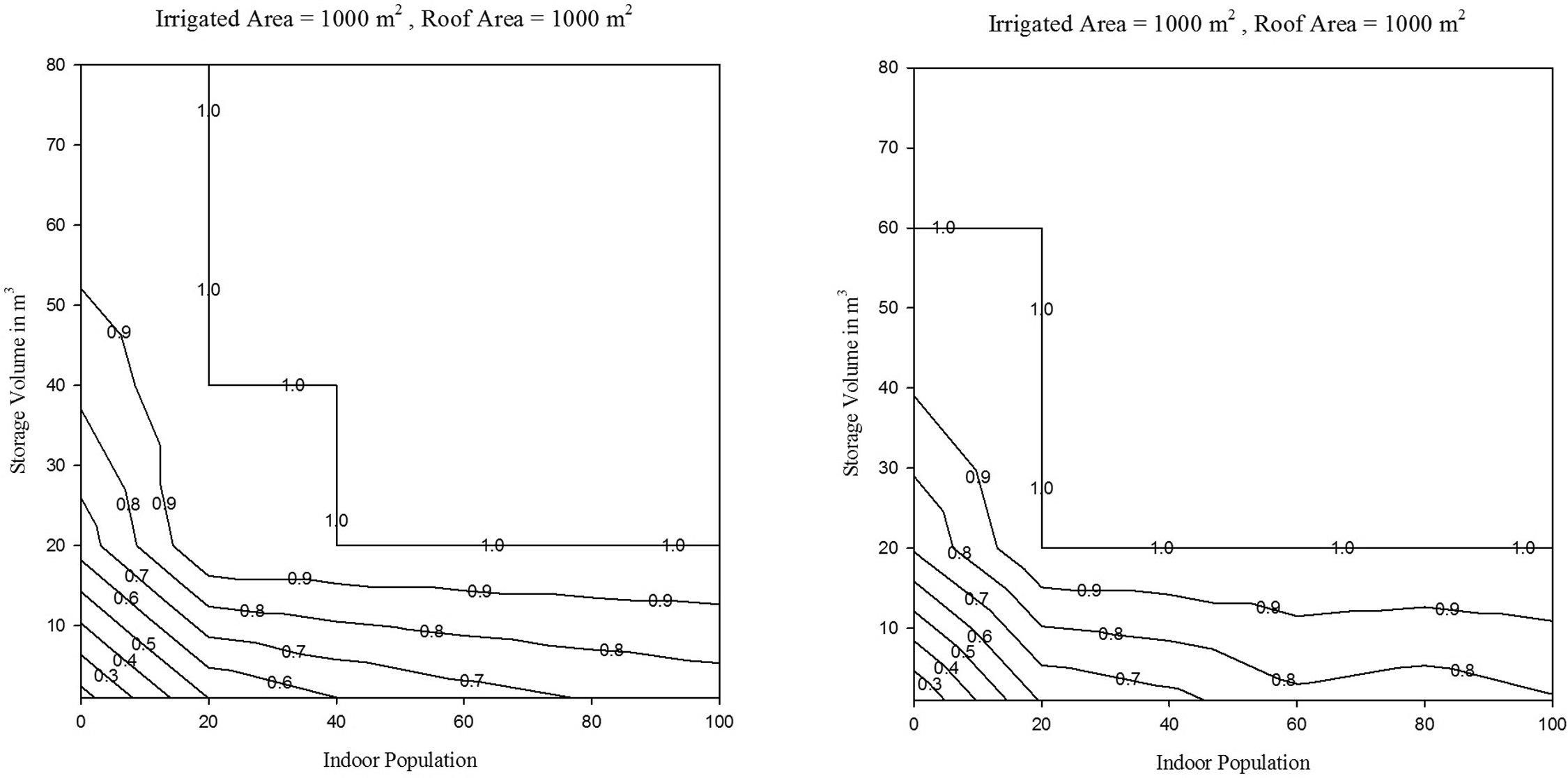

Supplement: Supplementary file 5 — Supplementary material [file mmc5.zip › D26.jpg]

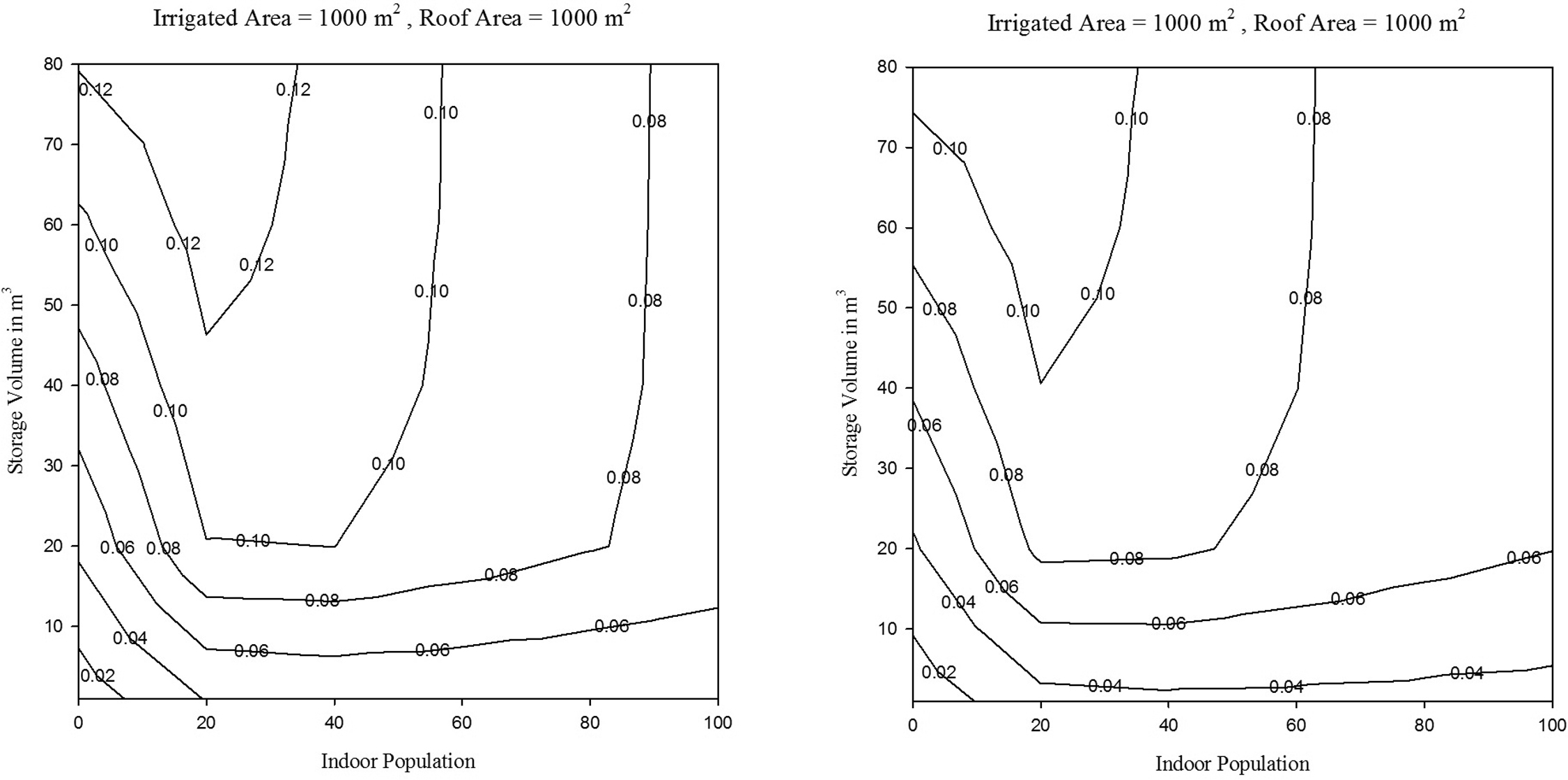

Supplement: Supplementary file 5 — Supplementary material [file mmc5.zip › D27.jpg]

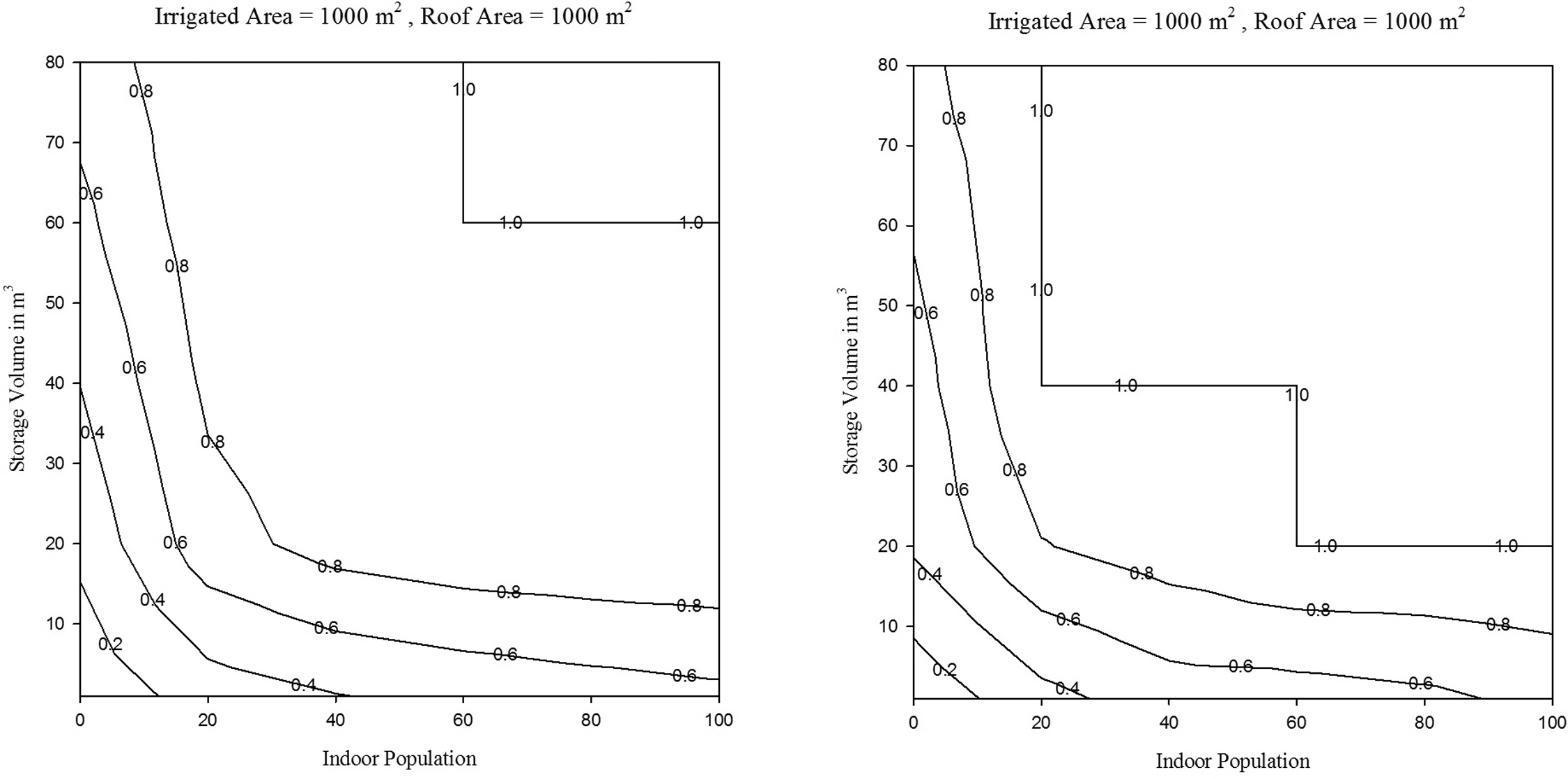

Supplement: Supplementary file 5 — Supplementary material [file mmc5.zip › D28.jpg]

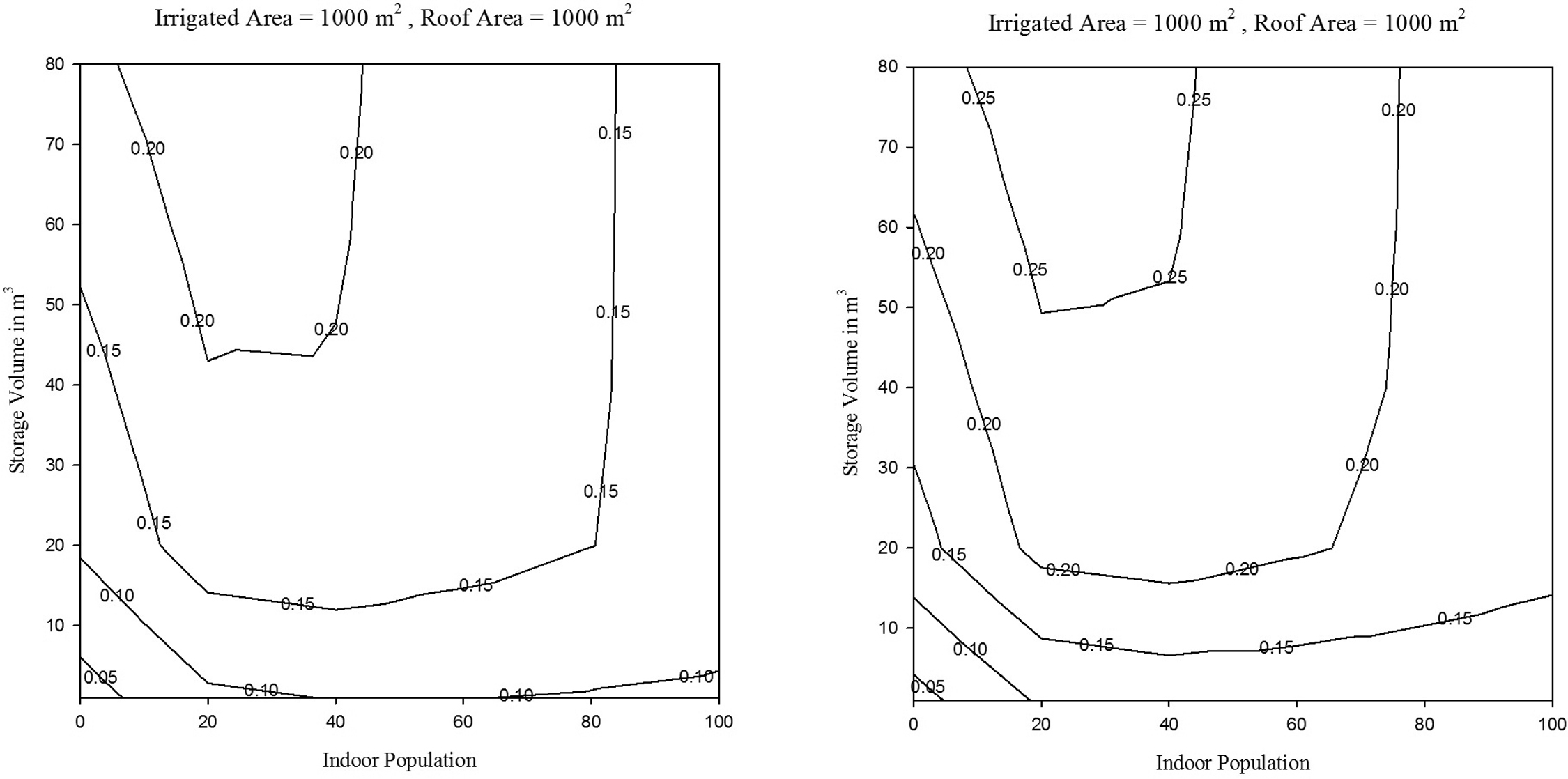

Supplement: Supplementary file 5 — Supplementary material [file mmc5.zip › D29.jpg]

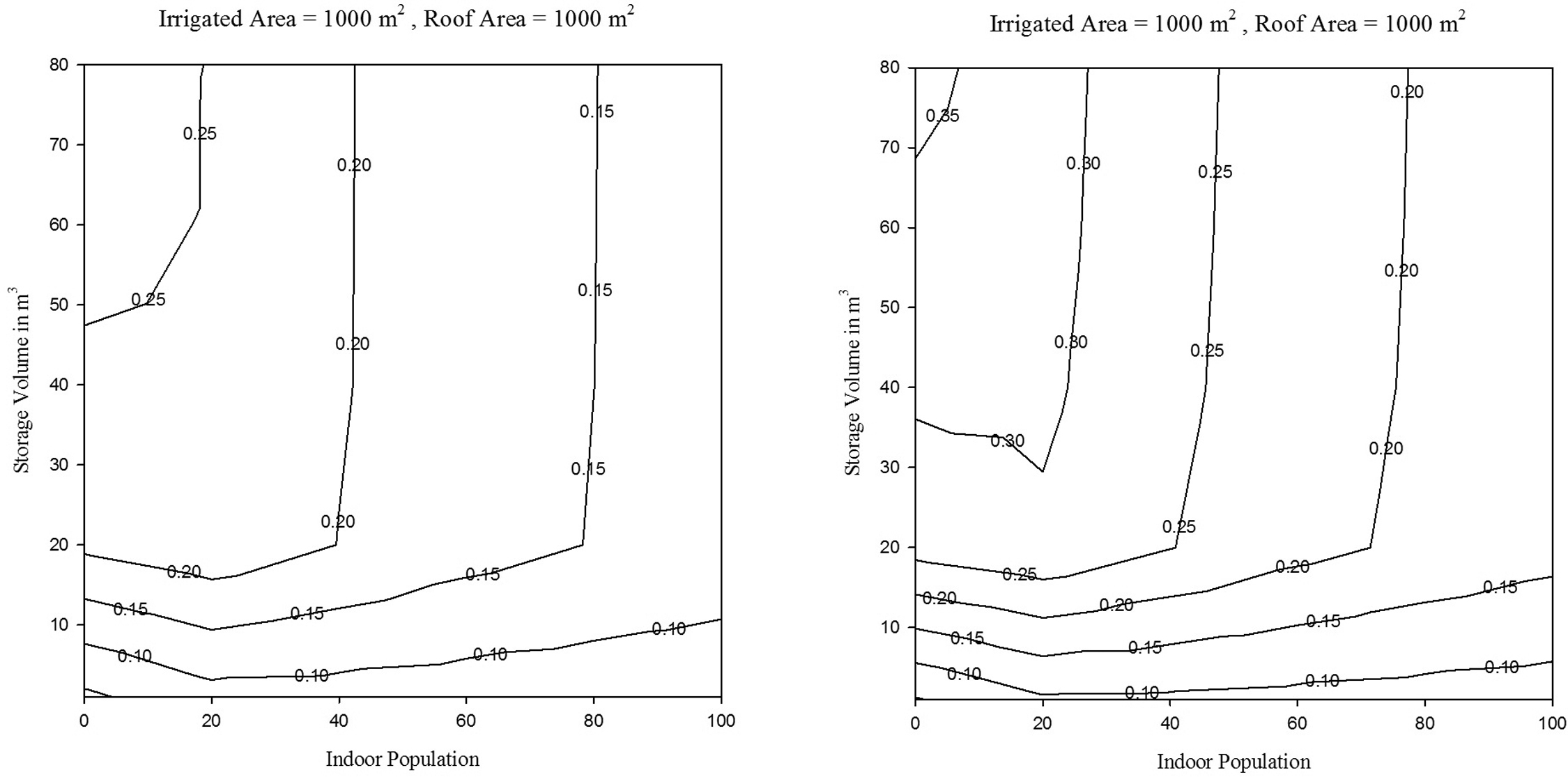

Supplement: Supplementary file 5 — Supplementary material [file mmc5.zip › D3.jpg]

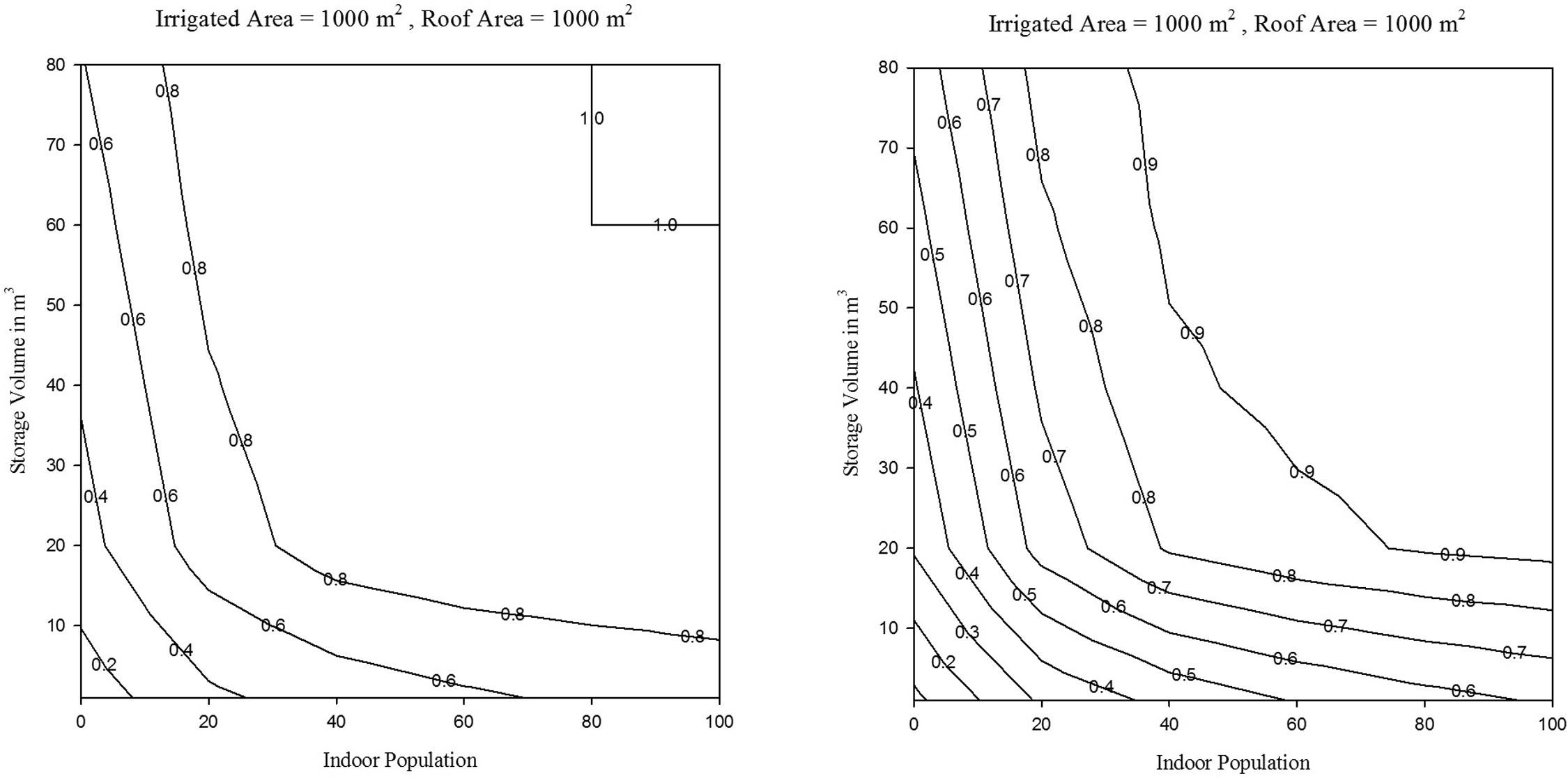

Supplement: Supplementary file 5 — Supplementary material [file mmc5.zip › D30.jpg]

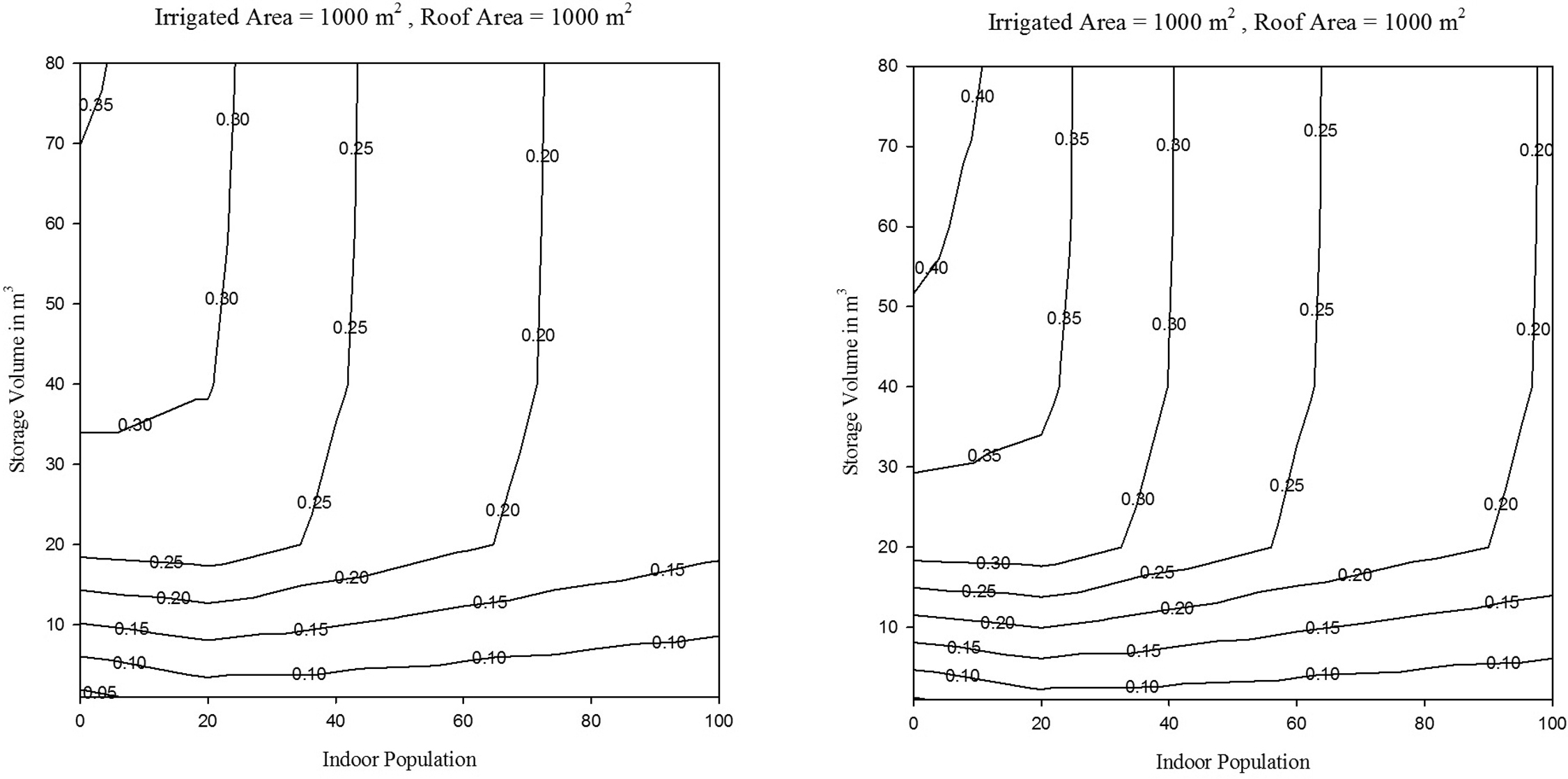

Supplement: Supplementary file 5 — Supplementary material [file mmc5.zip › D31.jpg]

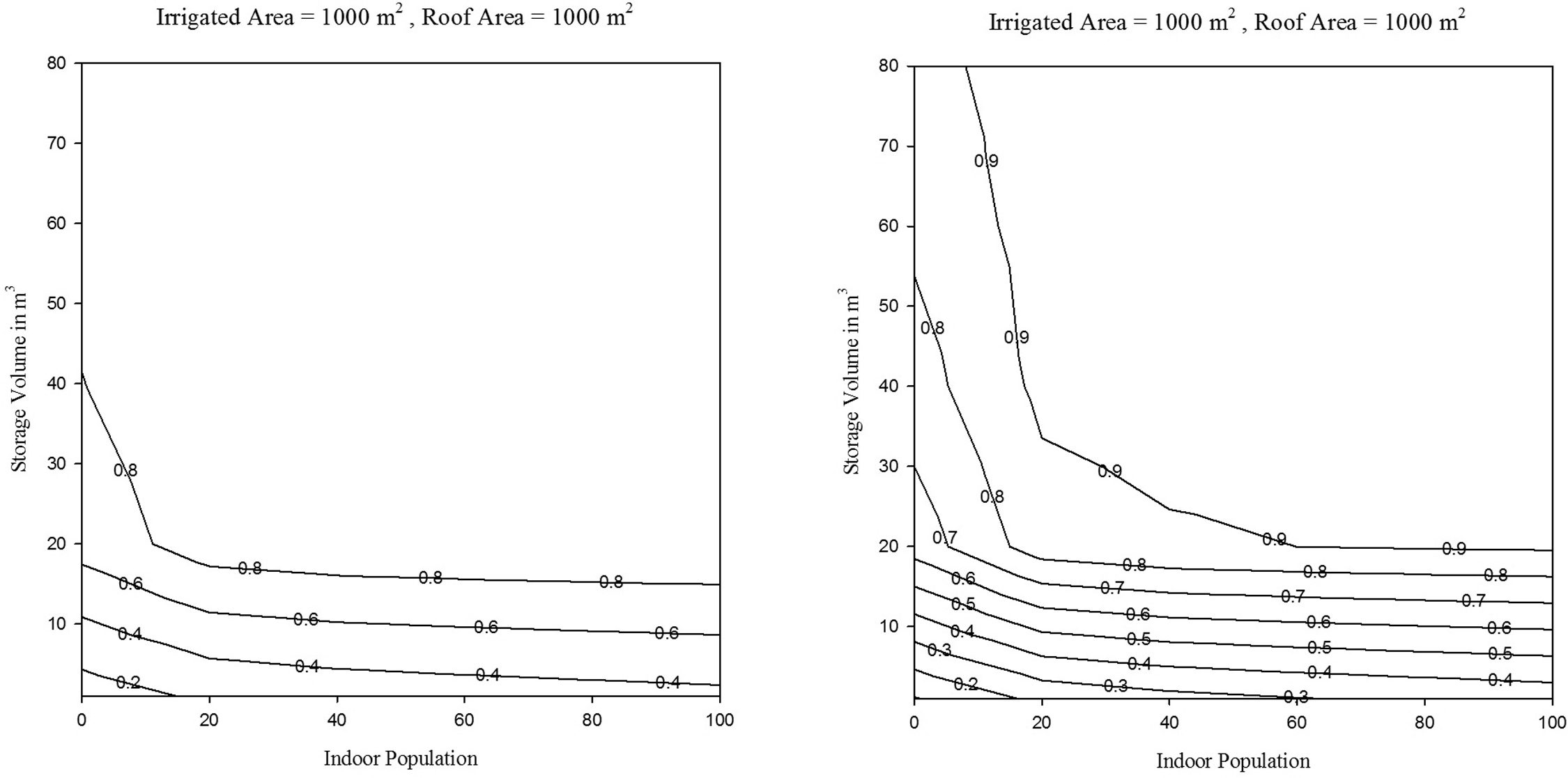

Supplement: Supplementary file 5 — Supplementary material [file mmc5.zip › D32.jpg]

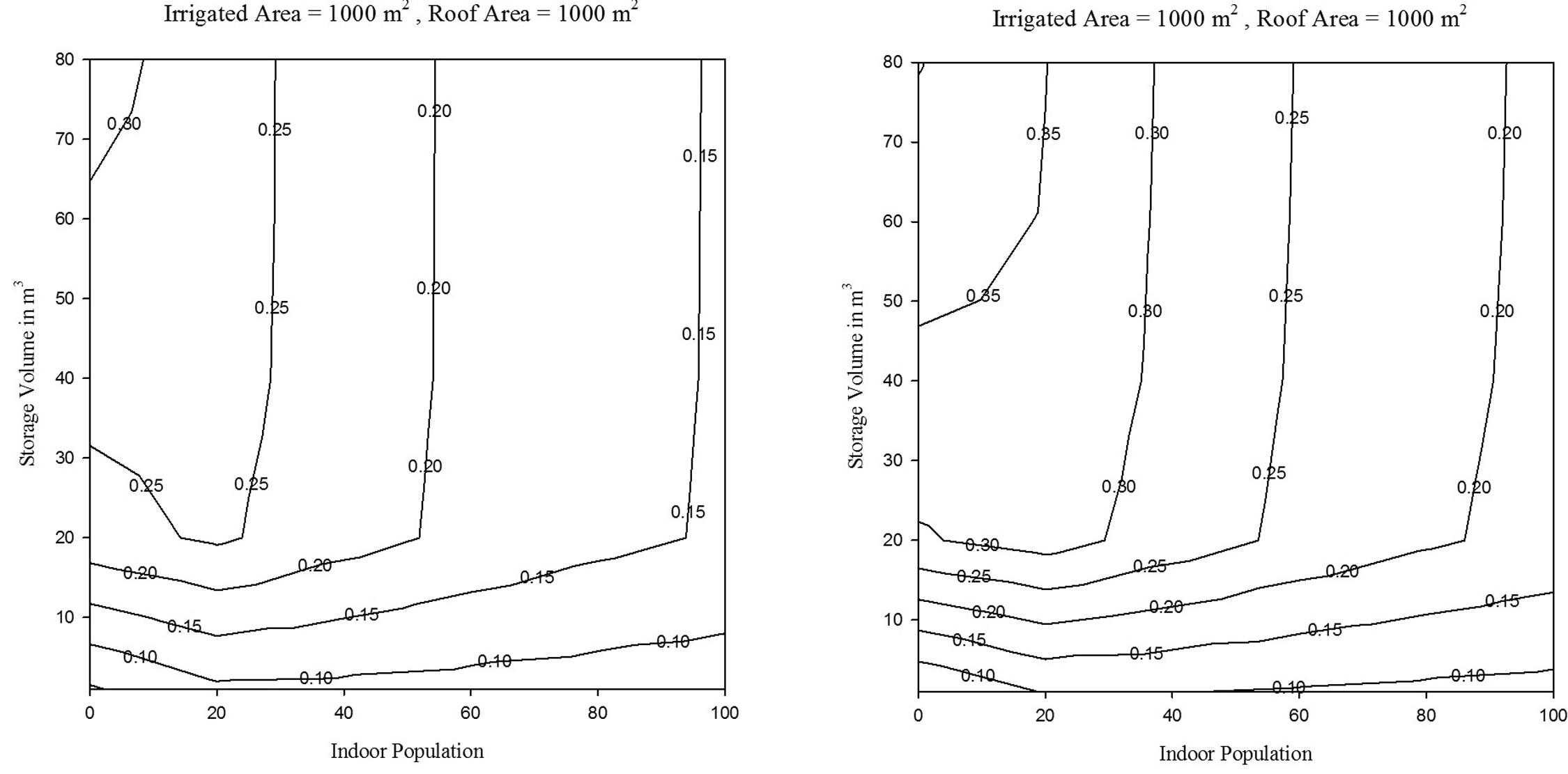

Supplement: Supplementary file 5 — Supplementary material [file mmc5.zip › D33.jpg]

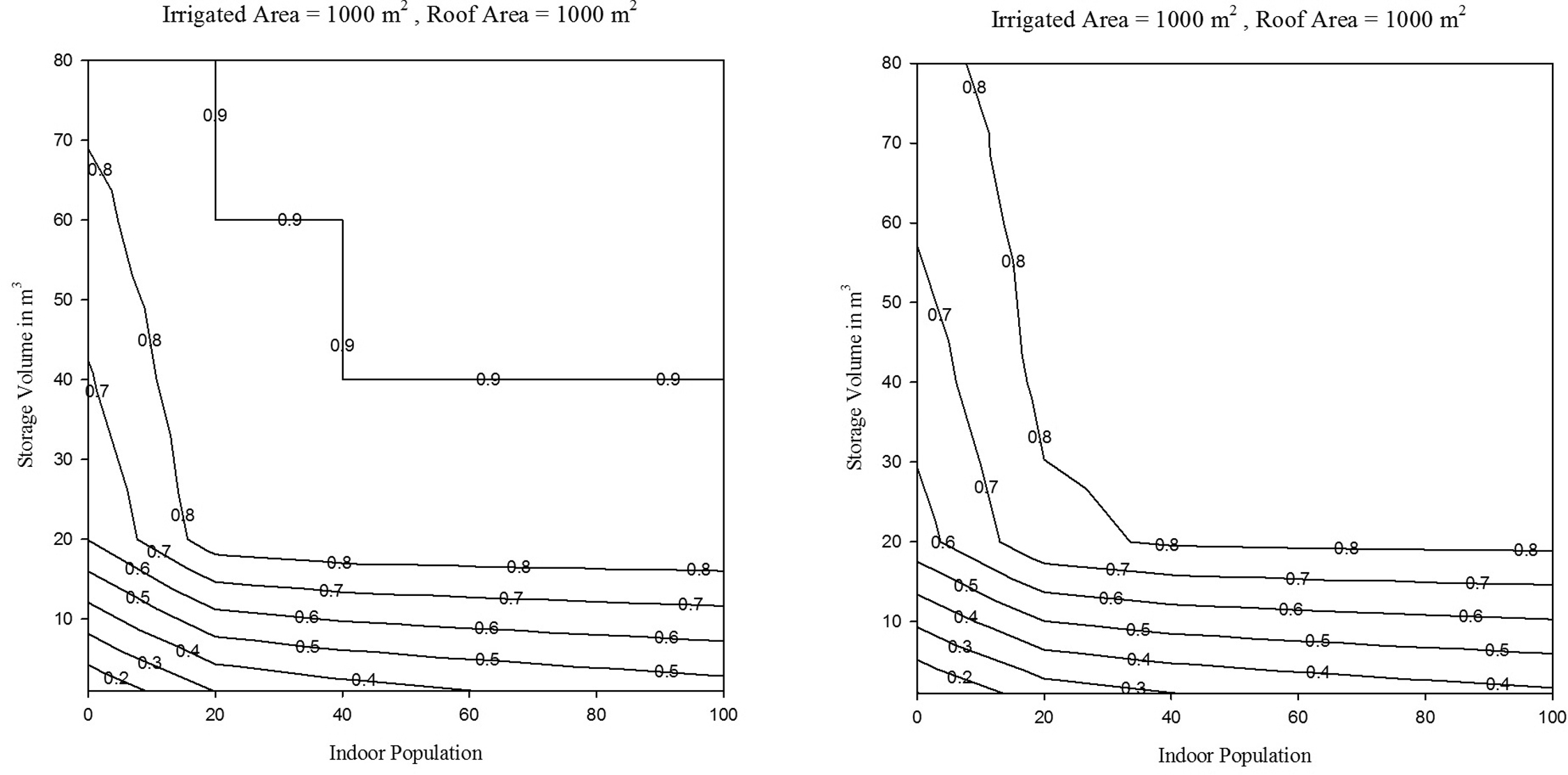

Supplement: Supplementary file 5 — Supplementary material [file mmc5.zip › D34.jpg]

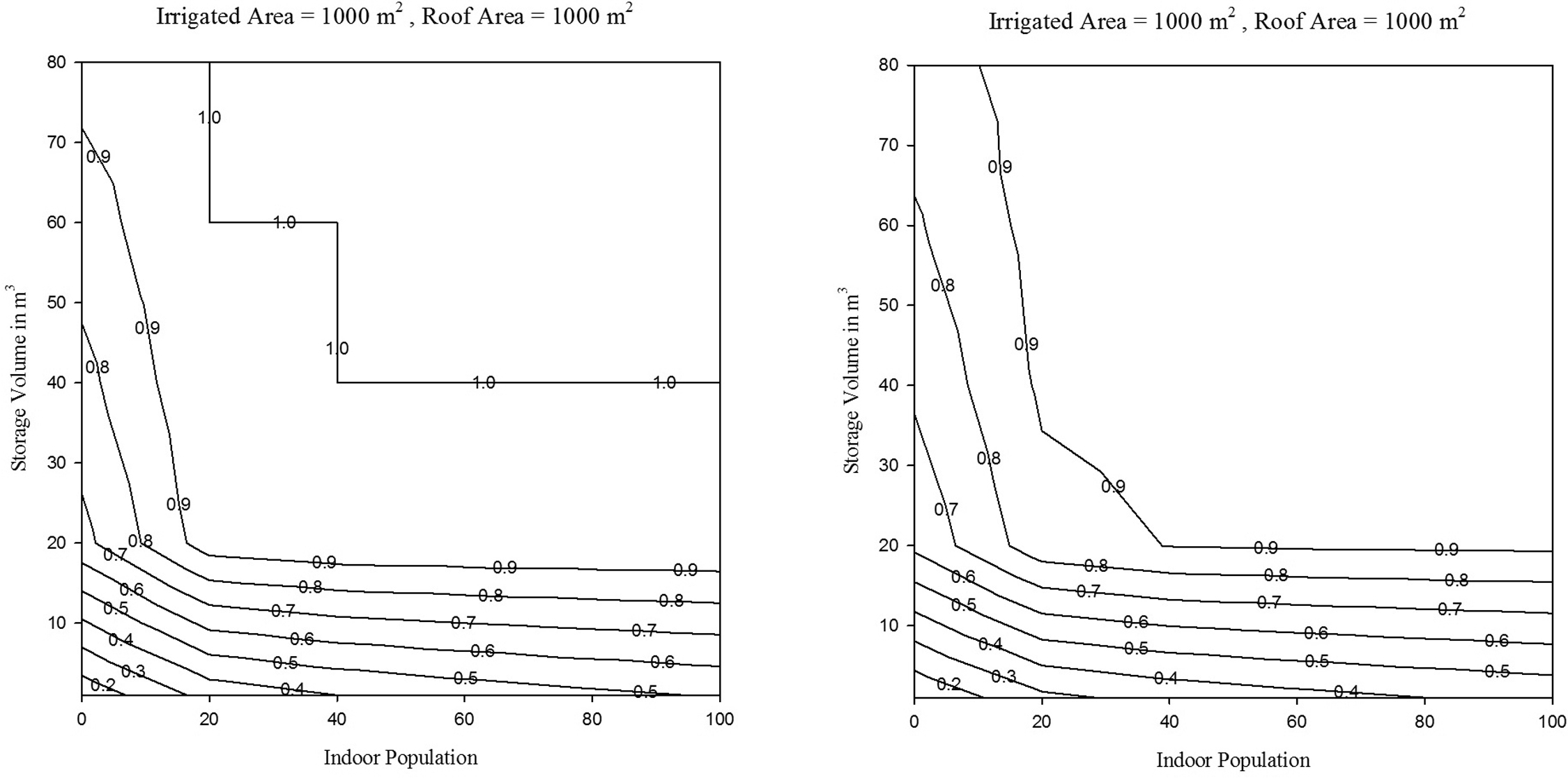

Supplement: Supplementary file 5 — Supplementary material [file mmc5.zip › D4.jpg]

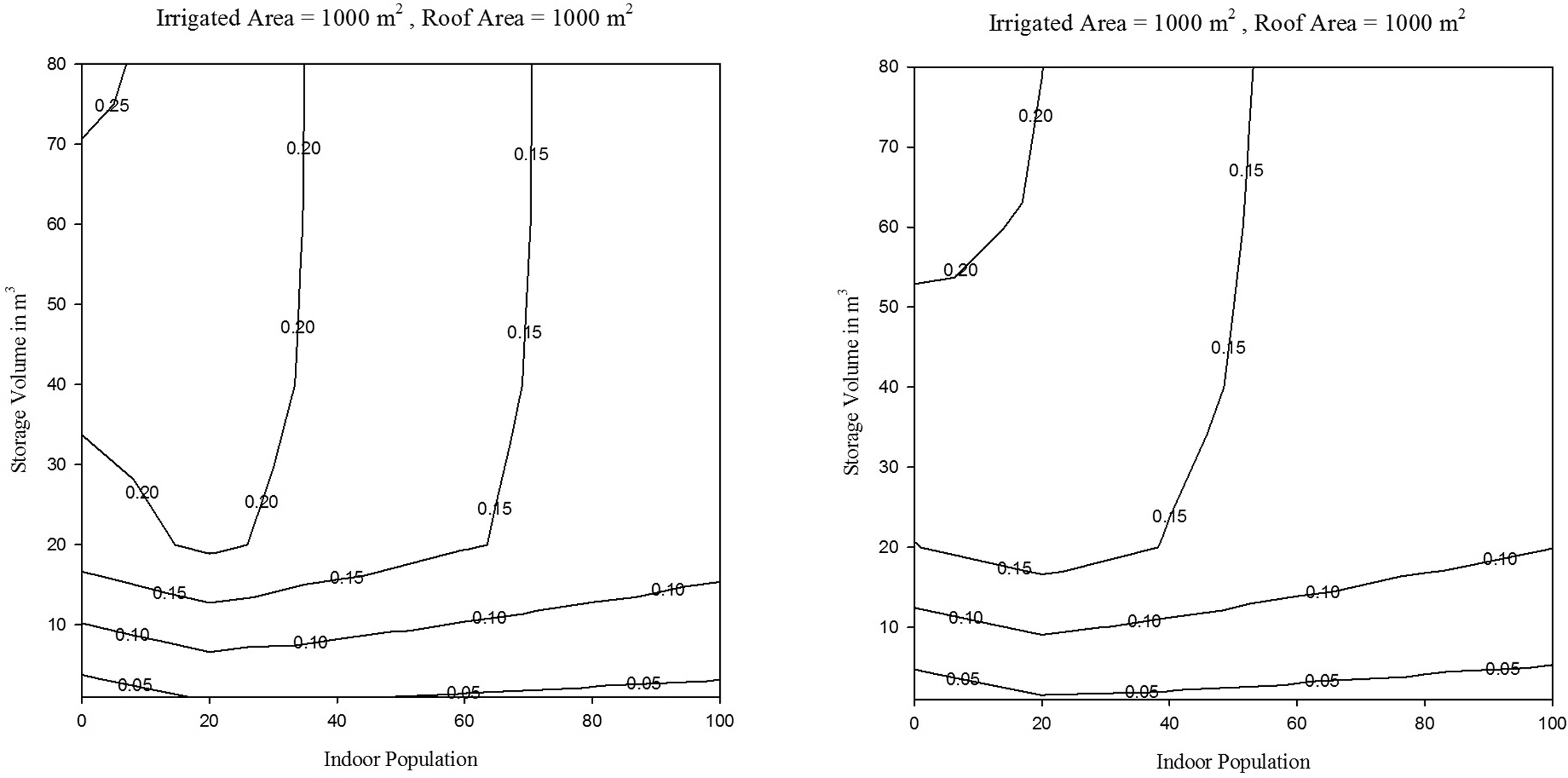

Supplement: Supplementary file 5 — Supplementary material [file mmc5.zip › D5.jpg]

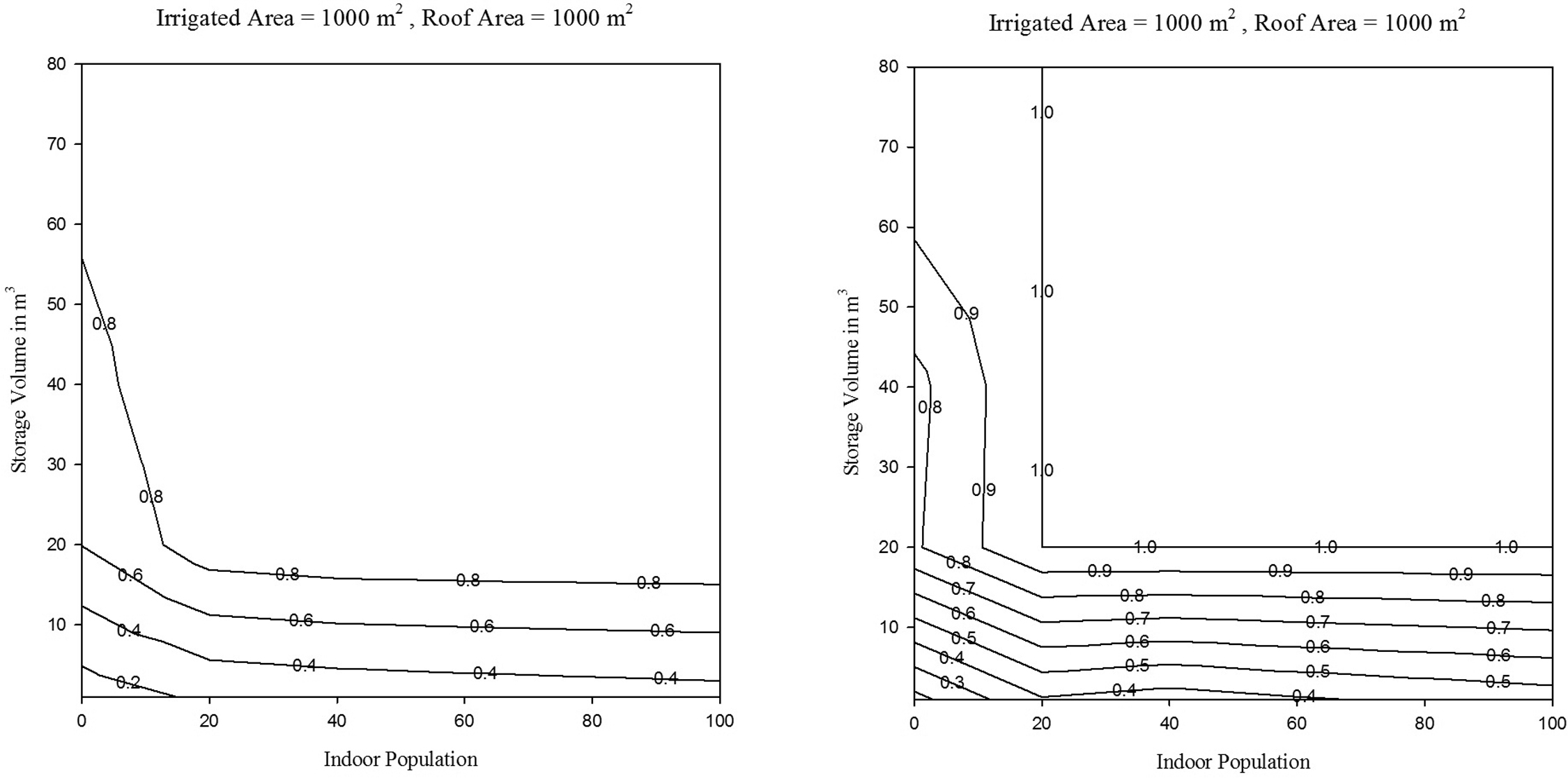

Supplement: Supplementary file 5 — Supplementary material [file mmc5.zip › D6.jpg]

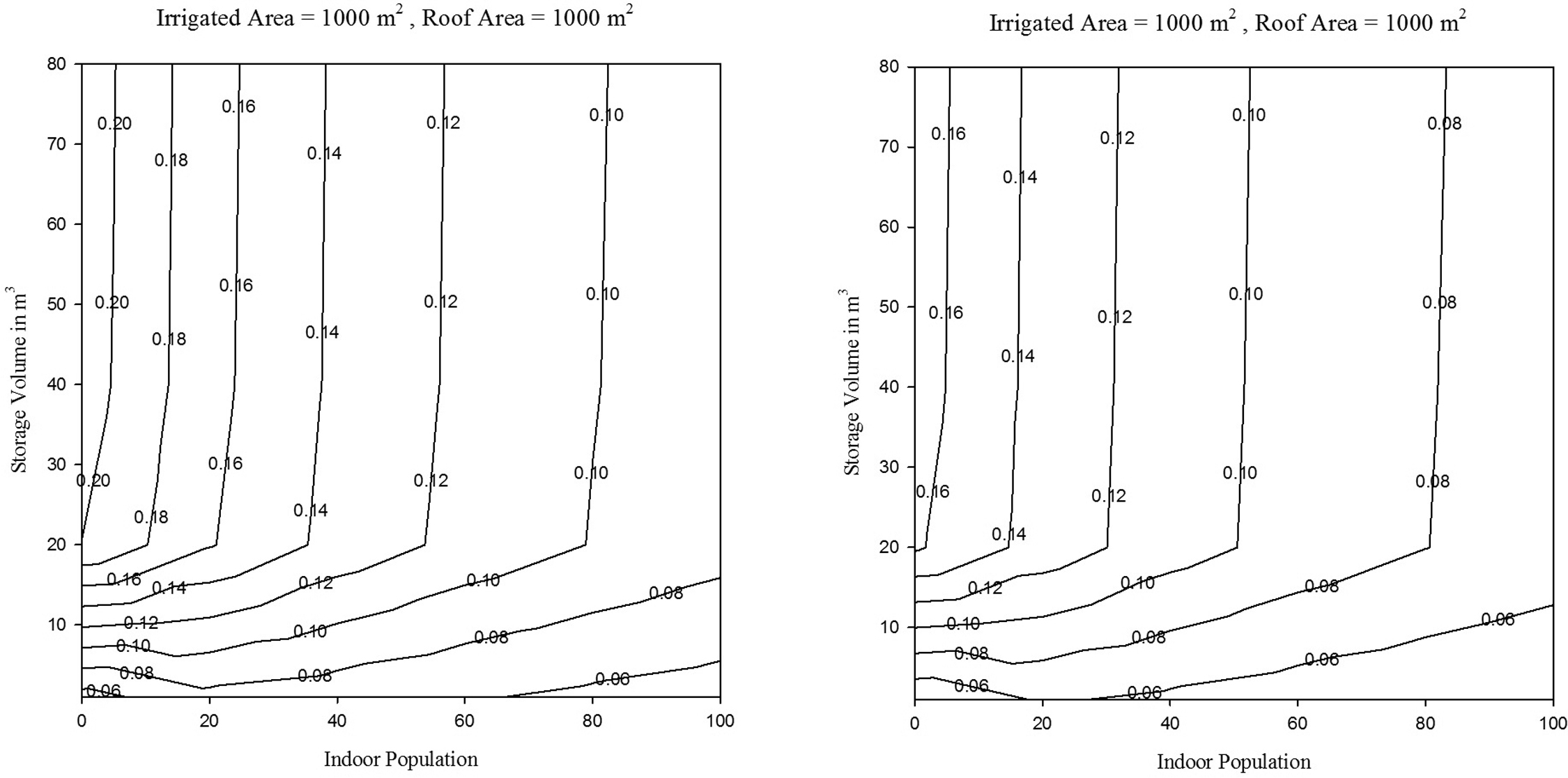

Supplement: Supplementary file 5 — Supplementary material [file mmc5.zip › D7.jpg]
