# Supplementary figures and images for: Water supply and runoff capture reliability curves for hypothetical rainwater harvesting systems for locations across the U.S. for historical and projected climate conditions (part 2 of 2)
Source: Data Brief. 2018 Mar 11;18:441–7. doi: 10.1016/j.dib.2018.03.024 (PMC5996225; doi:10.1016/j.dib.2018.03.024)

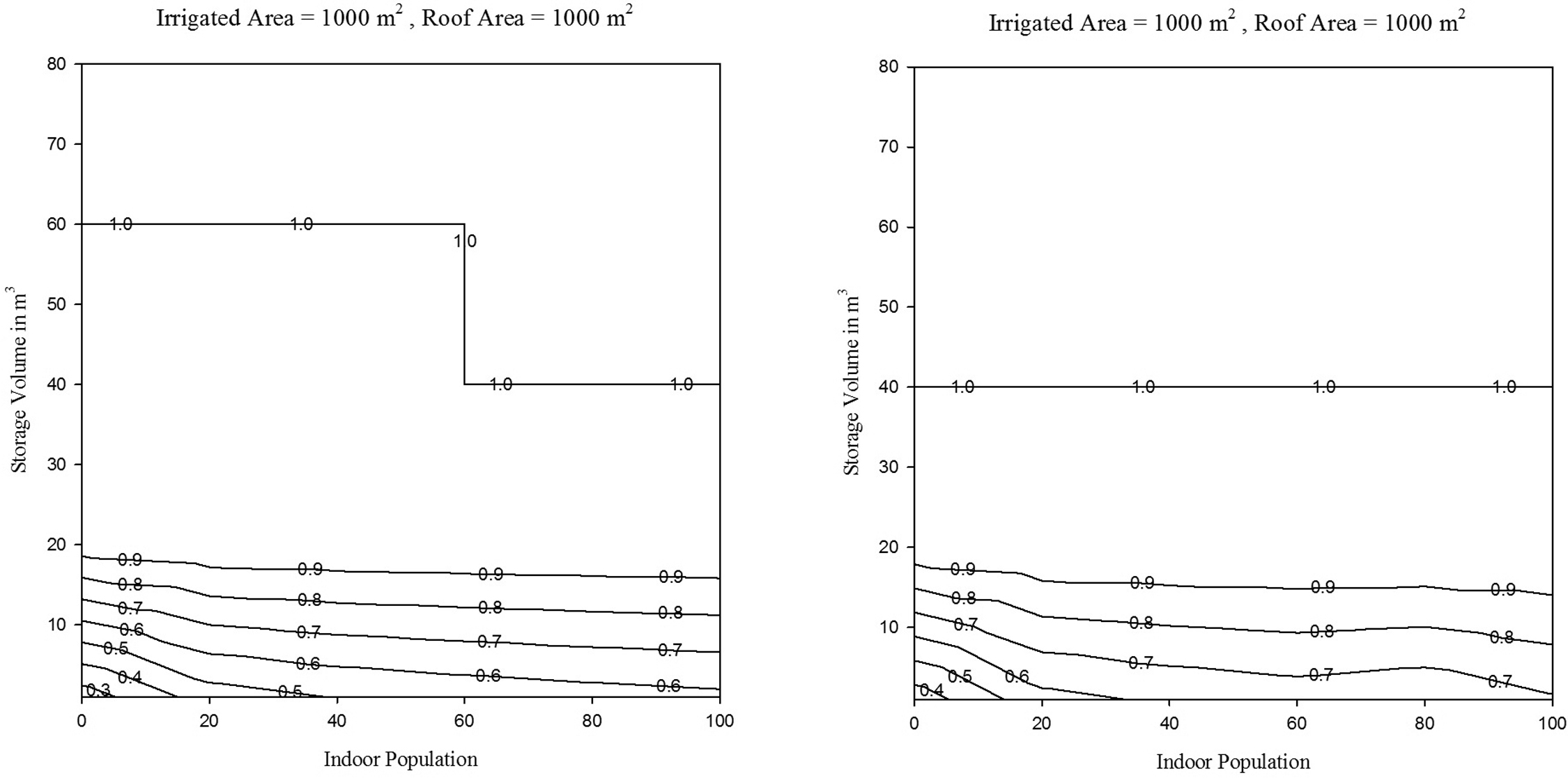

Supplement: Supplementary file 5 — Supplementary material [file mmc5.zip › D8.jpg]

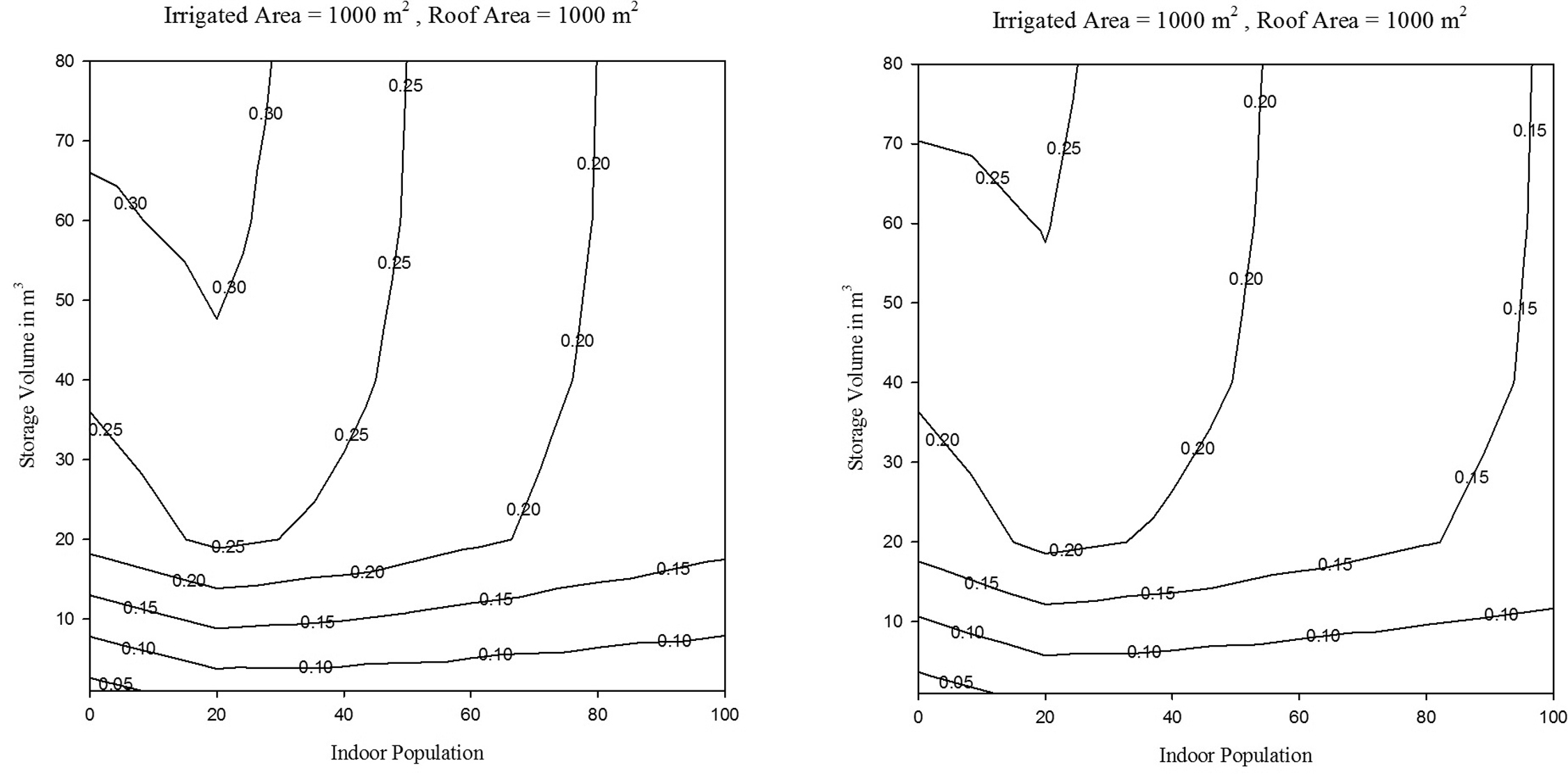

Supplement: Supplementary file 5 — Supplementary material [file mmc5.zip › D9.jpg]
